# Selective Functionalization of Graphene Oxide Unlocks Synergistic Catalysis for Organophosphate Degradation

**DOI:** 10.1021/acsomega.6c07874

**Published:** 2026-10-19

**Authors:** Bernardo M. Régnier, Tue Hassenkam, Maria Luiza M. Rocco, Aldo J. G. Zarbin, Elisa S. Orth

**Affiliations:** † Department of Chemistry, Universidade Federal do Paraná (UFPR), CP 19032, CEP, 81531-980 Curitiba, PR Brazil; ‡ Globe Institute, 552766 University of Copenhagen, Oester Voldgade 5-7, Copenhagen 1350, Denmark; § Instituto de Química, Universidade Federal do Rio de Janeiro (UFRJ), CEP, 21941-909 Rio de Janeiro, RJ Brazil

## Abstract

Site-selective functionalization of nanomaterials such as graphene oxide (GO) is a powerful strategy to tailor catalytic performance, with the degree and positioning of functional groups being critical for targeted applications. Herein, we present a systematic study on the functionalization of GO with ethylenediamine through two distinct pathways: (i) attachment at carboxylic acid groups located at the sheet edges and (ii) attachment at epoxide groups distributed across the basal plane. Comprehensive characterization, including XPS, AFM-IR, among others, was consistent with the site-specific functionalization. Importantly, the positioning effect was directly correlated with catalytic activity in the heterogeneous degradation of hazardous organophosphates—compounds widely used in agrochemicals and notorious for their extreme toxicity and persistence in the environment. Our results reveal that functionalization at the epoxide sites, despite yielding a lower degree of modification, produced superior catalytic efficiency, achieving a rate constant of 4.86 min^–1^ g^–1^, in contrast to 0.721 min^–1^ g^–1^ for the nanocatalyst functionalized via the carboxylic acid. The higher catalytic rate constant was obtained due to synergistic interactions with freely available carboxylic acids acting as intramolecular catalysts. Blocking these carboxylic acids with inert groups led to a marked decrease in activity, emphasizing their mechanistic role. Beyond advancing toward functionalization strategies, this study demonstrates how nanodesign can be leveraged to probe catalytic mechanisms and enhance detoxification of organophosphates. Understanding these synergistic effects in GO-based materials opens new avenues for designing nanocatalysts aimed at mitigating the dual-use hazards of organophosphates, with broad implications for environmental remediation and public health protection.

## Graphical abstract

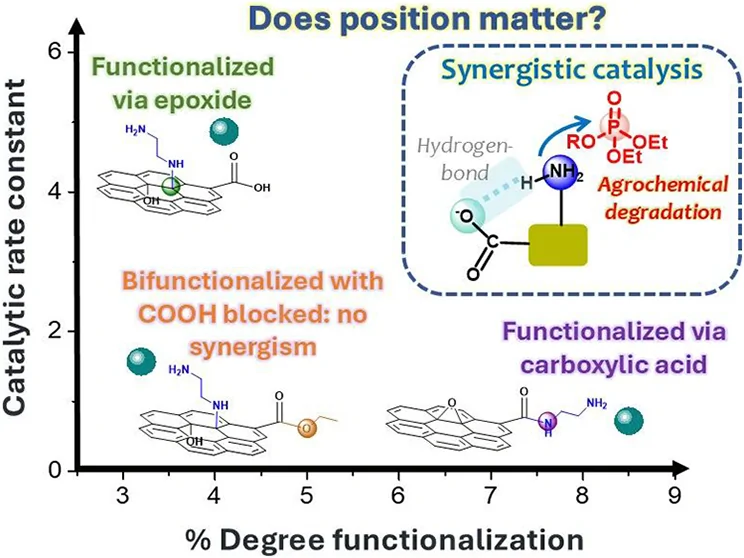

## Introduction

Nowadays, the detoxification of organophosphates (OPs), a class that contains agrochemicals and chemical warfare agents, is an emerging issue due to their high stability and toxicity.^1,2^ Nucleophilic catalysis is a good approach for the efficient neutralization of OPs, since they are usually able to react in a selective way that leads to less toxic products.^3,4^ Recent advancements have explored various functionalized platforms (containing imidazole, hydroxamate, or thiols, among others), including polymers, metal–organic frameworks, carbon-based nanomaterials (graphene oxide, reduced graphene oxide, and carbon nanotubes), and composites involving one or more of those showing selectivity under mild reaction conditions. These systems show enhanced stability, selectivity, and versatility in the degradation process of OPs under fairly mild reaction conditions.^5–13^

Graphene oxide (GO) is a great platform for grafting nucleophilic groups, since it has various oxygenated functional groups that can be used to place these nucleophiles, such as epoxides and carboxylic acids.^9,14,15^ These sites enable covalent bonding of functional groups through known selective reactions (Figure 1), making it possible to achieve targeted functionalization at specific positions on GO.^14^

**Figure 1:**
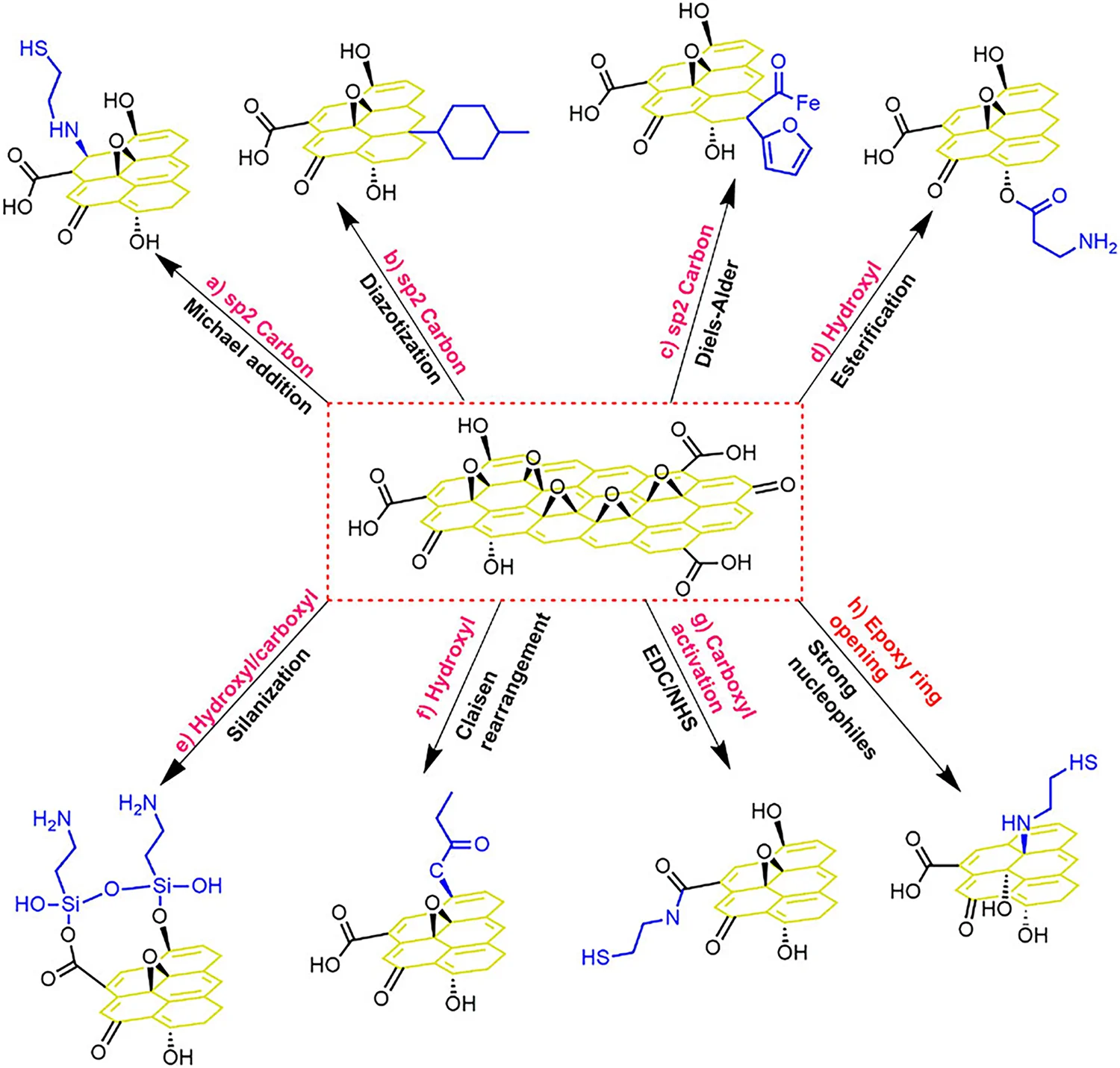
Examples of covalent functionalization through different routes in GO.^14^ Reproduced with permission under a Creative Commons CC-BY license (CC-BY 4.0) from ref 14. Copyright Frontiers Carbon.

Functionalization at the carboxylic acid sites has been broadly studied in many applications.^8,14,16^ On the other hand, epoxides have attracted attention more recently, since their functionalization is more challenging.^8,14^ Epoxides are predominantly located on the basal plane, while carboxylic acids are found at the sheet edges, making it possible to selectively functionalize either at the borders or the basal plane of the graphene oxide sheet.^17–19^ This selectivity can be achieved through synthetic routes designed for each of the two functional groups.^9^ While these observations are based on the assumed distribution of functional groups on GO, direct experimental evidence confirming this positional selectivity upon functionalization is challenging.^8,9,14^ The degree of functionalization is usually low, especially via the epoxide route, which makes it difficult to access positive signals related to the functionalized materials. In addition, the different targeted sites (e.g., acid or epoxide) share similar and/or close signals within different characterization techniques.^14^ More recently, complementary microscopic characterizations, coupled with spectroscopic techniques, have shown promising results for depicting the positioning of oxygenated groups on GO.^18,20^

It has already been reported that nucleophiles grafted on GO can have their catalytic activity enhanced by synergistic effects through neighboring oxygenated groups of GO.^8,9^ This effect occurs due to a general base catalysis mechanism by carboxylates, similar to the one found in enzymes, where a base can increase the nucleophilicity by cooperating via a hydrogen bond with a given nucleophile.^21^ Figure 2A shows three systems with this mechanism: (1) enzyme assisted by bivalent metal cations (α and β),^22^ (2) imine groups,^23^ and (3) thiol groups grafted in GO.^9^ This synergistic effect has been reported between thiol groups grafted in GO and carboxylates, as well as between these thiol groups and imidazole groups.^8,9^ Some evidence of this synergistic effect was found through XPS analysis, but more in-depth studies were lacking.

**Figure 2:**
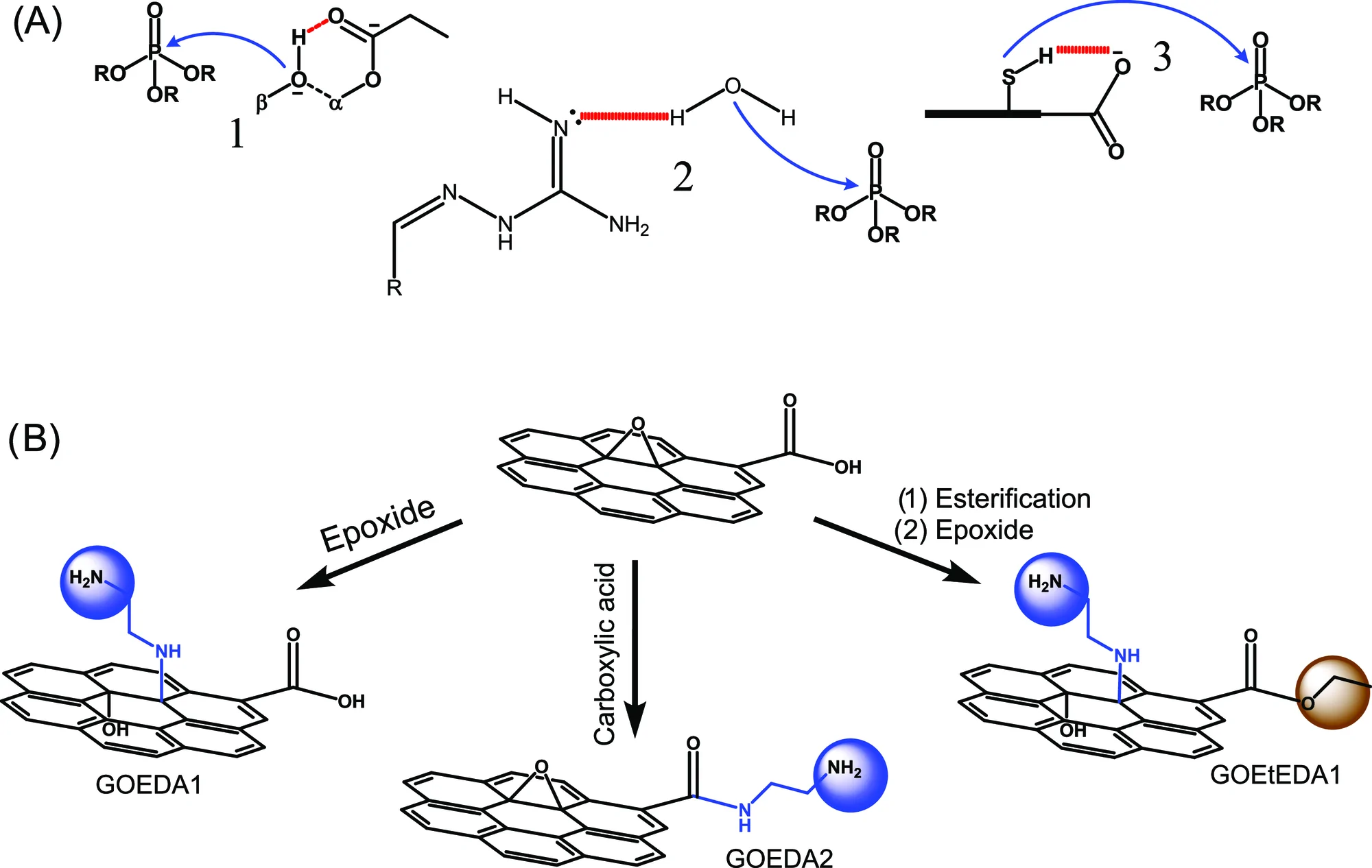
(A) Schemes showing the basic catalytic mechanism in (1) enzymes,^22^ (2) imines,^23^ and (3) thiol groups grafted in GO.^9^ (B) Representation of the synthetic approach herein, highlighting addition in the basal plane or the border and the blocked acid through esterification.

Wherein, we wanted to investigate how the selective positioning of nucleophiles on the basal plane and on the borders (Figure 2B) affects the performance of nanocatalysts in the heterogeneous catalytic neutralization reactions of OP. For that purpose, we selectively functionalized GO with ethylenediamine (EDA) at the epoxide (GOEDA1) and carboxylic acid (GOEDA2) sites. The carboxylic acid was blocked with an inert group by esterification with ethanol—leading to a bifunctionalized material, GOEtEDA—to corroborate the base catalysis mechanism by the freely available carboxylates. We aimed to visualize the selective positioning for the functionalization by atomic force microscopy-based infrared spectroscopy (AFM-IR), a technique that provides IR spectral data with nanometer resolution. This technique enabled us to map where the functionalization occurred, and for GO-functionalized materials, to the best of our knowledge, this is one of the first reports to differentiate the targeted functionalization at carboxylic acids or epoxides. Interestingly, the positioning effect of the functionalization agreed with the catalytic trend toward the neutralization of toxic OP, strongly suggesting cooperative intramolecular basic catalysis. Therefore, the nanocatalysts developed exhibit enzymatic-like behavior. Understanding the relation between function and structure for GO materials, and being able to control is in our view a breakthrough for multiple applications—and improving their performance through nanometer-scale design.

## Experimental Section

### Materials

EDA, 1-ethyl-3-(3-(dimethylamino)­propyl)­carbodiimide hydrochloride (EDC), *N*-hydroxysuccinimide (NHS), 1-chloro-2,4-dinitrobenzene (CDNB), and other reactants were obtained commercially. Graphite was obtained from National Graphite Company–Graflake 99580 99.84% and oxidized using a modified Hummers method to obtain the GO.^9^ The GO dispersion obtained was concentrated by rotary evaporation at 40 °C until a concentration of 4 mg/mL. The model organophosphate diethyl 2,4-dinitrophenylphosphate (DEDNPP) was synthesized as described in the literature.^24^

### Functionalization with EDA at the Epoxide Sites

The functionalization was based on the reaction of amines with epoxides under alkaline conditions that lead to the epoxide ring opening^14,25^ in a S_N_2-like reaction. In a 20 mL GO dispersion (4 mg/mL), 0.29 mL (4.4 mmol) of EDA (purified by distillation) was added, the pH was set to 10 using NaOH and HCl (1 mol/L), and the reaction was maintained under magnetic stirring at 60 °C for 6 h. At the end of the reaction, 50 mL of ethanol was added, and the solid was washed with ultrapure water and centrifuged—this process was repeated three times. The material was separated by filtration using a micropore 0.22 μm filter, and the solid was dried in an oven at 50 °C. This material was named GOEDA1.

### Functionalization with EDA in the Carboxylic Acid Sites

The grafting of nucleophilic functional groups at the carboxylic sites was achieved by using the coupling agents EDC and NHS, which selectively target carboxylic acids.^8^ To a 20 mL GO dispersion (4 mg/mL), 0.68 g of NHS (5.91 mmol) and 1.14 g of EDC (5.94 mmol) were added, and left under magnetic stirring for 1 h in an ice bath (∼4 °C) and then 1 h at room temperature (25 °C). After that, 0.29 mL of EDA (4.4 mmol) was added, the pH was adjusted to 10 and the reaction was left under magnetic stirring for 12 h. The same cleaning/separation process applied to GOEDA1 was applied here, and this material was denominated GOEDA2.

### Blocking the Carboxylic Acids

For blocking the carboxylic acids, first we carried out the esterification at the carboxylic acid sites with ethanol (EtOH) to produce GOEt (characterization shown in the SI). The procedure was similar to that described for GOEDA2, but instead of adding EDA, 4.4 mmol of EtOH (0.26 mL) was added. Then, in a 20 mL GOEt dispersion of 4 mg/mL, 0.29 mL (4.4 mmol) of EDA was added, the pH of the medium was set to 10 using NaOH and HCl (1 mol/L), and the reaction was maintained under magnetic stirring for 12 h. The cleaning/separation processes described in GOEDA1 were applied as well for this material and resulted in a material called GOEtEDA.

### Characterization

The FTIR measurements were carried out in transmission mode, using KBr pellets (KBr was dried in a muffle for 3 h at 300 °C previously to pellet preparation). A Bruker (Invenio R) device was used with a resolution of 2 cm^–1^ and 128 scans in the range of 4000–400 cm^–1^.

TGA analyses were performed using the TGA-DSC TA Instruments equipment, Model: SDT Q600. For the analysis, 3 mg of sample was used, and an isothermal step of 1 h at 70 °C was carried out to remove residual water. Subsequently, temperature increments of 5 °C/min were applied up to a maximum of 1000 °C; all analyses were made in a synthetic air atmosphere.

The equipment used for the CHNS analysis was the CHNS Analyzer–Vario Micro Cube (Elemental). The analyses were performed in duplicate, with 2 mg of sample each, and 70 s of O_2_ were used. Since the TGA residues at 1000 °C consist mainly of some metallic oxides, the amount of oxygen in the samples was calculated by the difference between the CHNS residue (which consists of oxygen and metallic oxides) and the TGA residue at 1000 °C.

The AFM-IR measurements were performed with a nanoIR2 instrument (AFM-IR) from Anasys Instruments, Inc., which combines an AFM and a pulsed tunable IR source, making it possible to obtain IR maps at one wavelength alongside topography maps. All the measurements of AFM-IR were conducted in contact mode, with a gold-coated tip and using a Si wafer or a Si wafer with a freshly stripped gold layer on top as the sample substrate. An IR background calibration and an alignment of the laser were made to achieve an optimal response. The IR spectra were acquired over the 1300–1800 cm^–1^ range, with a resolution of 4 cm^–1^/pt and 128 co-averages. To acquire IR mappings, the AFM scan rate was reduced to 0.1 Hz with a co-average of 16.

The SEM images were obtained using a TESCAN Mira FEG-SEM instrument equipped with an in-beam detector (10 kV voltage). The powdered material was deposited on a copper tape substrate. The energy-dispersive X-ray spectroscopy (EDS) spectra were acquired using an OXFORD Instruments detector coupled to the microscope.

The XPS spectra were acquired using a Thermo Fisher Scientific Escalab 250Xi spectrometer. Monochromatic Al Kα excitation energy (*h*ν = 1486.6 eV) with an X-ray spot size of 650 μm was used for the XPS measurements. The deconvolutions were performed using a linear combination of Gaussian and Lorentzian line shapes to fit the spectra, while the Shirley function was applied for background correction.

### Kinetics Studies—Catalytic Activity and Mechanism Validation

The catalytic activity was investigated using the OP model DEDNPP, by monitoring the appearance of the product 2,4-dinitrophenol (2,4-DNP) band at 400 nm over time^26^ in a heterogeneous medium. A solution (10 μL) of DEDNPP (10^–3^ mol/L in acetonitrile) was added to 2 mL of buffered KHCO_3_ solution (0.001 mol/L, pH 8.5). An initial 1.5 mL aliquot was transferred to a quartz cuvette and analyzed with an Agilent Cary60 UV-Vis, and the spectrum was recorded in the 200–800 nm range. After returning the solution to the reaction medium, 1.8 mg of catalyst was added. The mixture was stirred at 1000 rpm (21 °C) for 15 min using a thermoshaker and then centrifuged at 7200 rpm for 30 min. Then, 1.5 mL of it was transferred to the cuvette for another spectrophotometric scan. This process was repeated until the reaction was complete. The catalytic activity was also studied at different pH values, using the buffers NaH_2_PO_4_ or KHCO_3_ (0.001 mol/L).^27^

To verify the selectivity of the nanocatalysts, another reaction using the material functionalized via epoxide (GOEDA1) was conducted. In this case, the reaction of GOEDA1 was studied with 1-chloro-2,4-dinitrobenzene (CDNB) (10^–3^ mol/L in acetonitrile) to analyze the aromatic carbon reactivity. Reactions were carried out under the same conditions and method as the reaction with DEDNPP.

Absorbance versus time data for all reactions were used to calculate observed first-order rate constants (*k*_obs_)^12^ using Origin software. Nonlinear fits were carried out using the Levenberg–Marquardt algorithm for at least 90% of the reaction. The rate constant obtained was normalized by the total mass (thus given in min^–1^ g^–1^) of the catalysts to facilitate comparison.^9^

The recycling was carried out by using the same material in 5 different reactions with DEDNPP, in the same conditions as described earlier. Between each reaction, the material was washed five times with ultrapure water. After each washing step, the sample was dried overnight at 40 °C, before it was used again.

## Results and Discussion

The overall aim of this study was to functionalize GO with EDA in two different positions, basal plane or borders, toward the epoxide (GOEDA1) or carboxylic acid (GOEDA2), respectively. Then, we sought to elucidate how the anchoring functional group affects its catalytic activity due to shifts in synergistic behavior. The methodologies adopted were specific for each target site, and previous evidence shows that they do not lead to promiscuous side reactions.^9^ A bioinspired basic catalytic mechanism is proposed to account for the synergistic effects, where carboxylate groups already present on GO can enhance the nucleophilicity of specific groups anchored on it through a hydrogen bond (Figure 2A). To illustrate this, we also obtained a bifunctionalized material in which the carboxylic acid sites were blocked with an inert group (GOEtEDA). In this case, an esterification of GO was conducted with EtOH on the carboxylic acid sites, followed by the functionalization with EDA at the epoxide sites. Therefore, 3 different nanomaterials were obtained and employed as catalysts in the nucleophilic degradation reaction of a toxic organophosphate.

First, FTIR experiments were performed. Figure 3 shows the spectra obtained for GO, GOEDA1, GOEDA2, and GOEtEDA. For GO, the bands at 3738 and 3434 cm^–1^ correspond to hydroxyl groups stretching without and with hydrogen bonding, respectively.^28,29^ The bands at 2925 and 2851 cm^–1^ are attributed to asymmetric and symmetric CH_2_ stretching. In the region below 2000 cm^–1^, it is possible to identify a small band around 1700 cm^–1^ that might correspond to carboxylic acids (C=O stretching), and a band at 1577 cm^–1^ is attributed to aromatic C=C bonds. The band at 1632 cm^–1^ can be attributed to adsorbed water.^30–32^ A few bands attributed to epoxide groups were found at 1166 cm^–1^ (C–O stretching) and 1062 cm^–1^ (C–O–C bending). Additionally, shoulders on either side of the band at 1113 cm^–1^ can also be assigned to epoxide ring stretching.^9,28^

**Figure 3:**
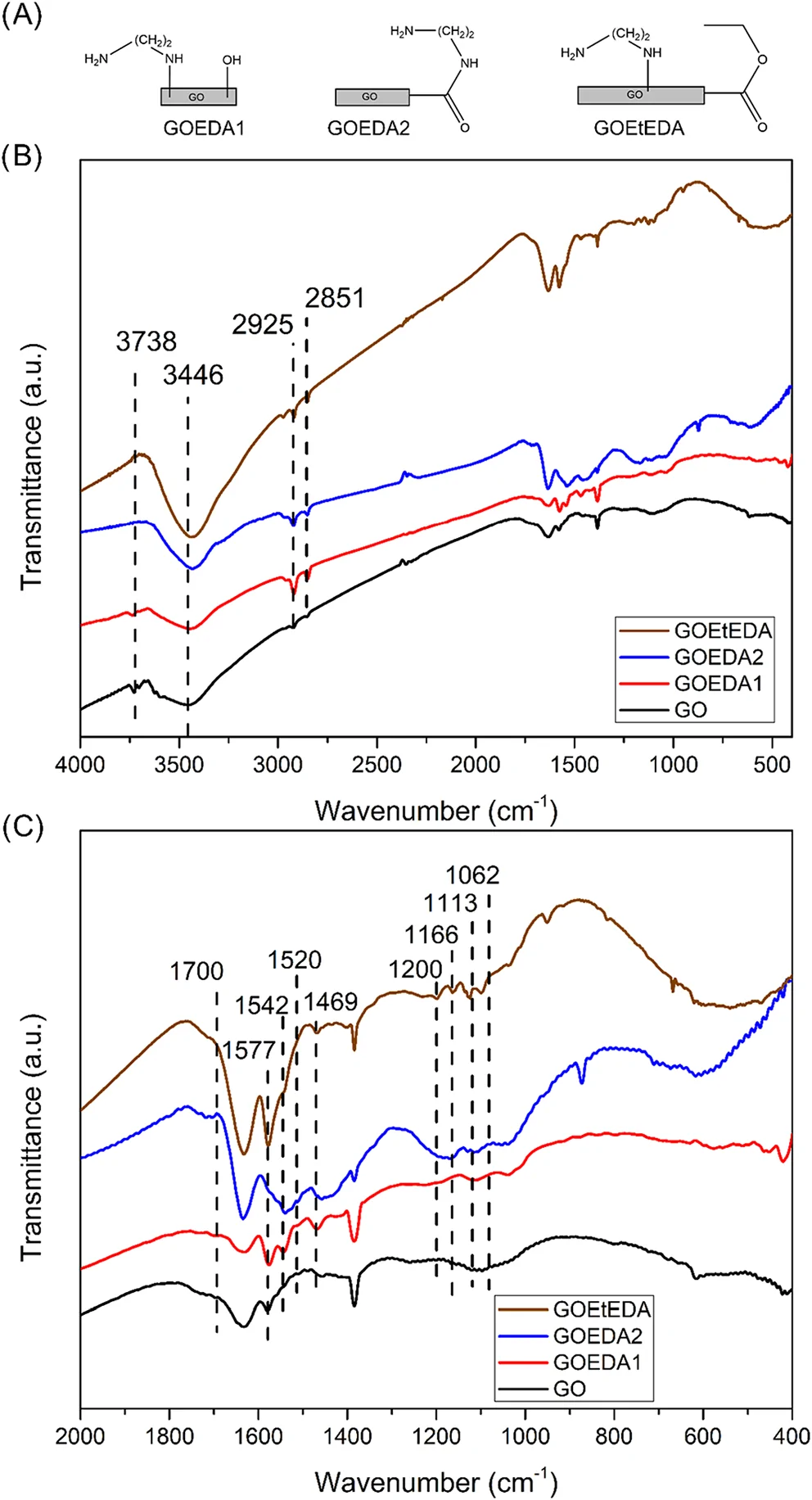
(A) Simplified structure of GOEDA1, GOEDA2, and GOEtEDA. (B) FTIR spectra of GO, GOEDA1, GOEDA2, and GOEtEDA1 in the range of 4000 to 400 cm^–1^ and (C) 2000 to 800 cm^–1^.

The typical bands of GO were preserved after functionalization, indicating that the GO structure remained without significant changes throughout the grafting process. The spectra for GOEDA1 (epoxide route) and GOEDA2 (carboxylic acid route) suggest covalent bonding between EDA and GO. The intensity of the isolated OH band at 3738 cm^–1^ decreased after both functionalization routes, while the CH_2_ bands at 2925 and 2851 cm^–1^ increased. New bands related to amines were observed: at 1542 and 1469 cm^–1^ (N–H and C–N bending, respectively) for GOEDA1, and at 1542 and 1520 cm^–1^ for GOEDA2, indicating the incorporation of EDA on the carbonaceous structure.^8,9,28^ The decrease in the intensity of the epoxide band at 1166 cm^–1^ for GOEDA1 and of the carboxylic acid band at 1700 cm^–1^ for GOEDA2 agrees with the selective reactions carried out at these sites.^9^ For GOEtEDA, the ester bands were found at 1200 cm^–1^.^29^ The region between 1200 and 1060 is heavily populated by C–O vibrations,^29^ making an assertive assignment hard to make, but when the same region of GOEtEDA is compared to GO and GOEDA, it is possible to observe more intense and well-defined signals, which might indicate a greater contribution from C=O of esters, that can differ from native carboxyl or carbonyls and from the functionalized epoxide groups. In addition, bands at 1577 and 1469 cm^–1^ are related to C–N bonds, suggesting multifunctionalization with EDA.^28,29^

The XPS survey spectra of GOEDA1 and GOEDA2 (available in the SI) present the main photoemission lines related to C 1s, N 1s, and O 1s, indicating functionalization, as the survey spectra of GO consist only of C 1s and O 1s photoemission lines,^9^ and N 1s photoemission lines were found for the functionalized ones. In the C 1s high-resolution spectra obtained for GO (Figure 4A), it is possible to observe peaks at 283.3 and 284.5 eV, related to C=C and C–C bonds, respectively, which are equivalent to 34.6% atomic percentage of carbon. Oxygenated groups in the C 1s spectrum represent 65.4%, of which 1.6% are carboxylic acids (289.9 eV), 17.3% are carbonyls (288.2 eV), and 46.5% are epoxides and hydroxyl groups (286.4 eV). The O 1s spectrum presents peaks related to C­(=O*)­OC, C=O, C–OH/C–O–C, and O*-(C=O)-C at 530.8, 531.9, 532.6, and 533.8 eV, respectively.^8,9,33,34^ All tables compiling XPS data are available in the SI.

**Figure 4:**
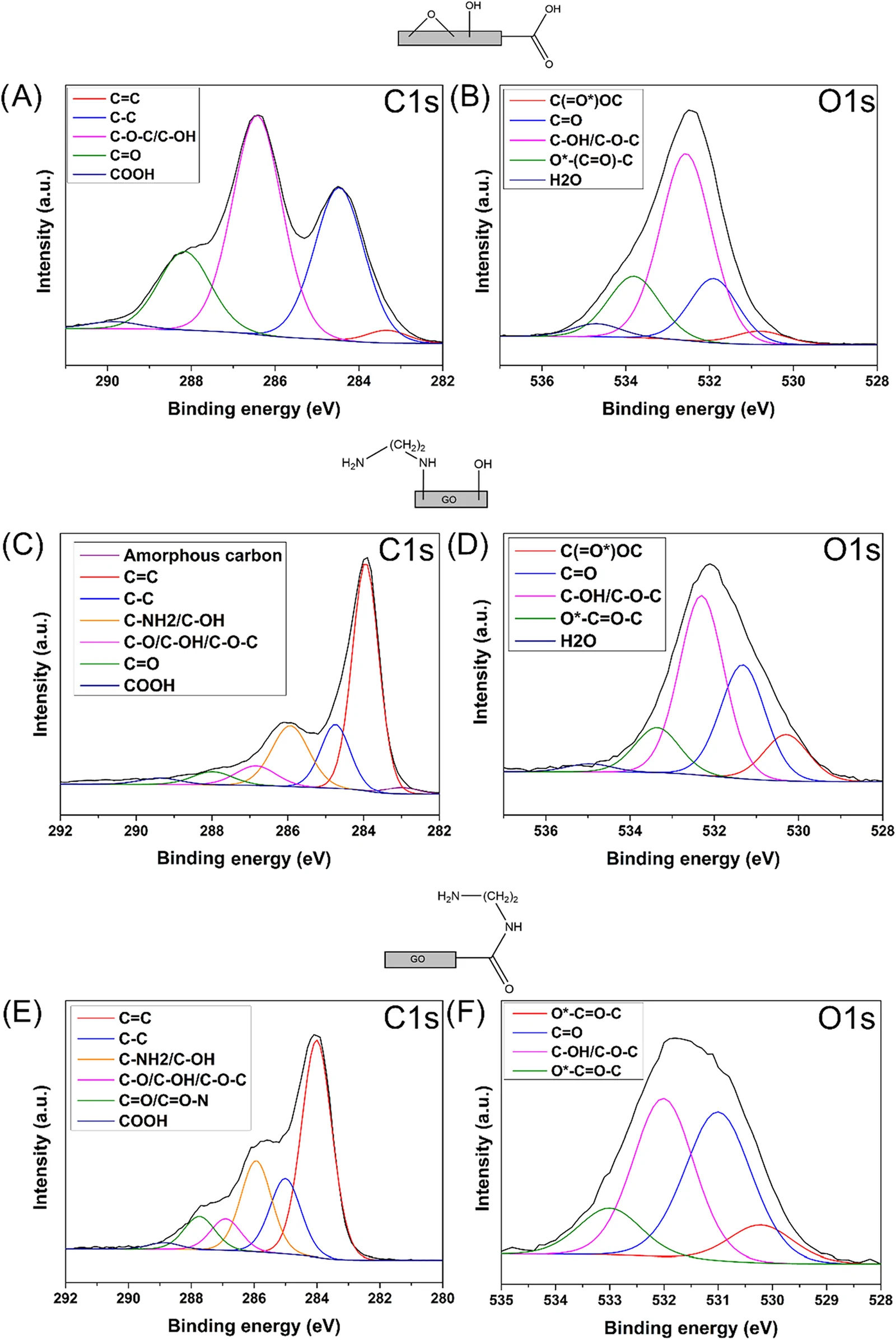
High-resolution XPS spectra for GO: (A) C 1s and (B) O 1s; GOEDA1: (C) C 1s and (D) O 1s; and GOEDA2: (E) C 1s and (F) O 1s.

Figure 4 also shows the representative high-resolution XPS spectra of C 1s and O 1s for GOEDA1 and GOEDA2, while N 1s spectra are available in the SI. The C 1s spectrum for the sample functionalized via epoxide (GOEDA1) presents peaks related to C=C (283.9 eV), C–C (284.7 eV), C–NH_2_ (285.9 eV), hydroxyls and epoxides (286.8 eV), C=O (288.0 eV), and COOH (289.4 eV).^9,33,34^ The epoxide peaks became less intense after functionalization, which suggests functionalization with EDA. Even though the epoxide peak in XPS has a contribution from hydroxyl peak in the same region, the N 1s spectra confirm C–N bonds of primary (398.7 eV) and secondary (399.7 eV) amines, without any amide formation. The deconvoluted spectrum of GOEDA2, which was functionalized via acid sites, presented nitrogen bond peaks at 285.9 eV. In the GOEDA2 O 1s spectrum, the C=O peak was found at 531.3 eV, hydroxyls and epoxides at 532.3 eV, and the COOH peak at 533.4 eV. The N 1s spectrum presents NH_2_, NH, and N^+^ peaks at 398.7, 399.7, and 401.4 eV, respectively.^9,33,34^ This indicates that an amide bond was formed in the reaction, suggesting that the EDA was added to the acid sites of GO.

The thermogravimetry data obtained for the samples are shown in detail in the SI (figures and comparative tables). The thermogravimetry of GO had two weight loss events related to oxygenated groups,^9,35^ as seen in the dTg: the first at 217 °C with a weight loss of 29.9% and the other at 295 °C with a loss of 6.1%. One last weight loss event found for GO is related to the oxidation of the carbonaceous framework, which occurred at 487 °C (55.4%).^35^ The functionalized samples, GOEDA1 and GOEDA2, showed that the first weight loss occurred at 175 °C for both materials, with GOEDA1 losing 21.6% and GOEDA2 with a loss of 9.5%. The decrease related to the oxygenated groups suggests the successful functionalization.^8,14^ The second weight loss is associated with nitrogenated and remaining oxygenated groups^8,9^ —at 314 °C for GOEDA1 (8.5%) and at 345 °C for GOEDA2 (26.5%)—indicating a greater functionalization for GOEDA2. This result is expected, since functionalization via carboxylic acid usually leads to a higher degree of functionalization than via the epoxide route.^14^ A third weight loss event was found, related to the carbonaceous framework. In this event, GOEDA1 lost 63.2% of its weight at 485 °C and GOEDA2 lost 62.7% at 623 °C. Unlike GOEDA2, which had only three losses, GOEDA1 exhibited a fourth weight loss, losing 7.4% of its weight at 623 °C that can be related to the sp^2^ network.^35^ The material with blocked acid sites (GOEtEDA) presented two events related to oxygenated groups. These events happened at lower temperatures and with lower weight losses when compared to GO. In the first, it lost 13% of its weight at 183 °C, and in the second weight loss event at 108 °C, it lost 11%; this may be related to the added ethyl groups. There was also a weight loss of 5.1% related to grafted nitrogen groups, found at 326 °C for GOEtEDA, which suggests success in the bifunctionalization. The event related to the carbonaceous structure of GOEtEDA was found in two separate weight loss events (as can be seen in dTg)—the first at 490 °C and the other at 563 °C (22.1 and 12.2%, respectively), suggesting modifications in the structure that may originate from multifunctionalization.^9^ The last event of GOEtEDA, around 650 °C, can be attributed to the sp^2^ network, with a weight loss of 12.2%.

The TGA analysis agrees with the functionalization of nitrogenated groups in GO, at both the carboxylic acids and epoxide sites. All samples demonstrated improved thermal stability upon functionalization and low residue levels, indicating high sample purity.

Elemental analysis was performed to calculate the percentage of each element in the samples, where the oxygen degree was calculated considering the TGA residue. Table 1 shows the carbon, oxygen, and nitrogen percentages obtained from the CHNS analysis.^36,37^ First, the presence of nitrogen confirms the overall functionalization. The degree of functionalization was considered the percentage of nitrogen for comparison purposes. GOEDA2 (via carboxylic acid) had a functionalization of 8.5%, GOEDA1 (via epoxide site) had 4.1%, and GOEtEDA (bifunctionalized blocked acids) had 3.2%. This agrees with the TGA analysis and indicates that the route via carboxylic acid is more grafted with EDA than that through the epoxide route. Comparing the oxygen content of the functionalized materials with GO, GOEDA1 had no significant change, since the epoxide route forms a hydroxyl with the ring opening.^14^ In comparison, for GOEDA2, the total oxygen significantly decreased compared to that of GO, due to the conversion of carboxylic acid to amides. For GOEtEDA, the total quantity of oxygen decreased slightly compared to GO and GOEDA1, but at the same time, it was the material with the highest percentage of carbon, suggesting that both esterification and grafting of EDA were successful.

**Table 1:** Elemental Percentage of Carbon, Oxygen, and Nitrogen from CHNS Analysis

sample	% C	% O	% N
GO	45.4 ± 2	42.0 ± 2	0.4 ± 0.1
GOEDA1	49.9 ± 0.3	42.8 ± 1	4.1 ± 0.2
GOEDA2	55.8 ± 0.2	28.2 ± 1	8.5 ± 0.2
GOEtEDA	57.0 ± 0.2	31.5 ± 0.1	3.2 ± 0.02

The SEM images of GO, GOEDA1, and GOEDA2 (Figure 5) revealed a continuous surface with a slight roughness and some stacked layers. No significant changes in the surface morphology were observed after functionalization. The EDS data for GOEDA1 and GOEDA2 showed a distribution of carbon and oxygen signals that correlate with the nitrogen signal, suggesting the successful incorporation of EDA groups across the GO sheets.

**Figure 5:**
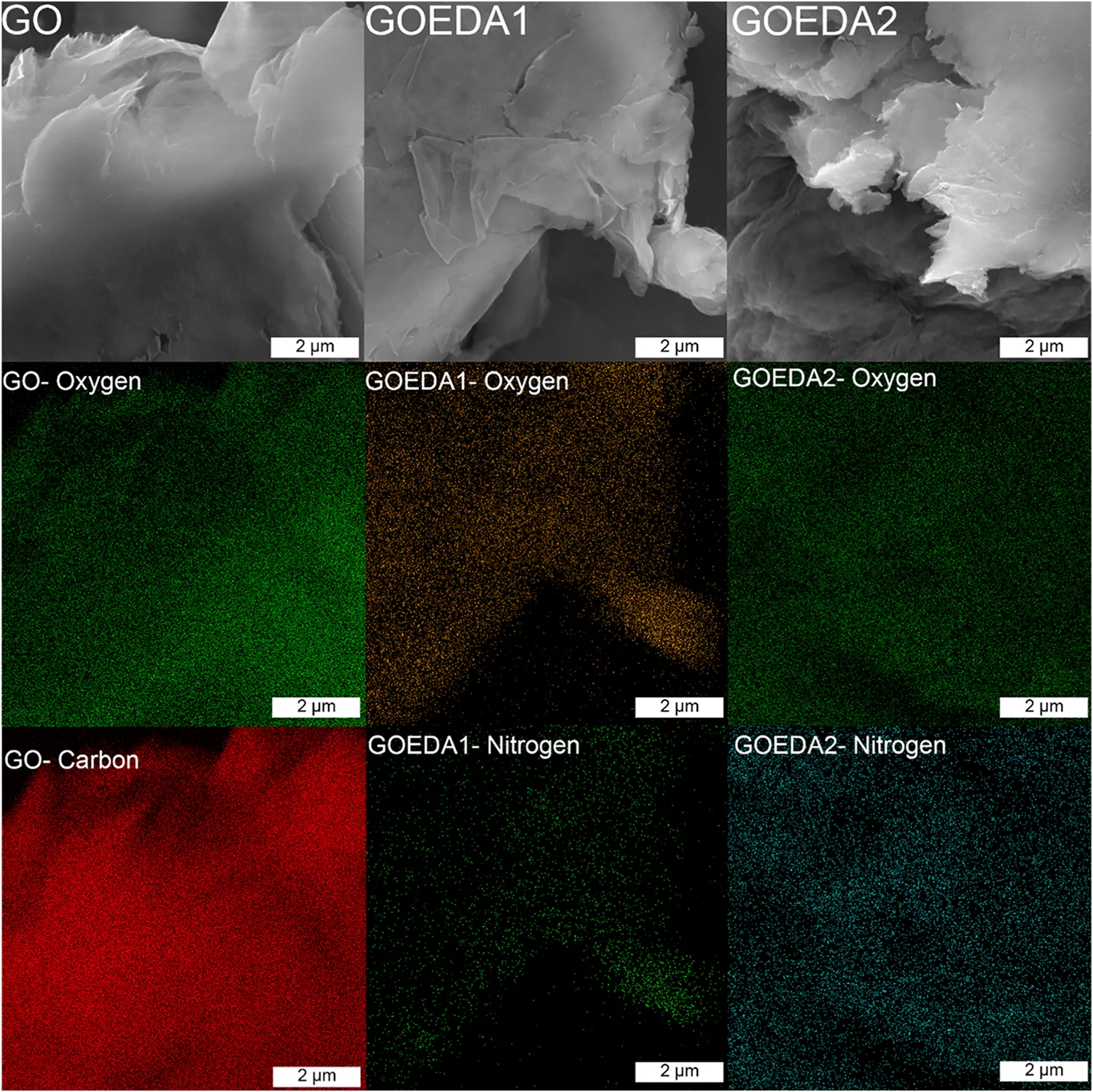
SEM and EDS images in 25xk amplification of GO, GOEDA1, and GOEDA2.

Further evidence of the site-selective functionalization was supported by the AFM-IR analysis, presented in Figure 6. The topography images of GOEDA1 and GOEDA2 are shown in Figure 6A,B, respectively, while Figure 6C,D show the IR spectrum obtained on specific spots on these samples. The topography images and the topography analysis, available in the SI, show functionalized GO sheets with 2 to 5 nm thickness and about 200 nm across for both analyzed samples, representing mainly single to a few layers. In addition, the analysis agrees with previous reports for bare GO.^18,20^

**Figure 6:**
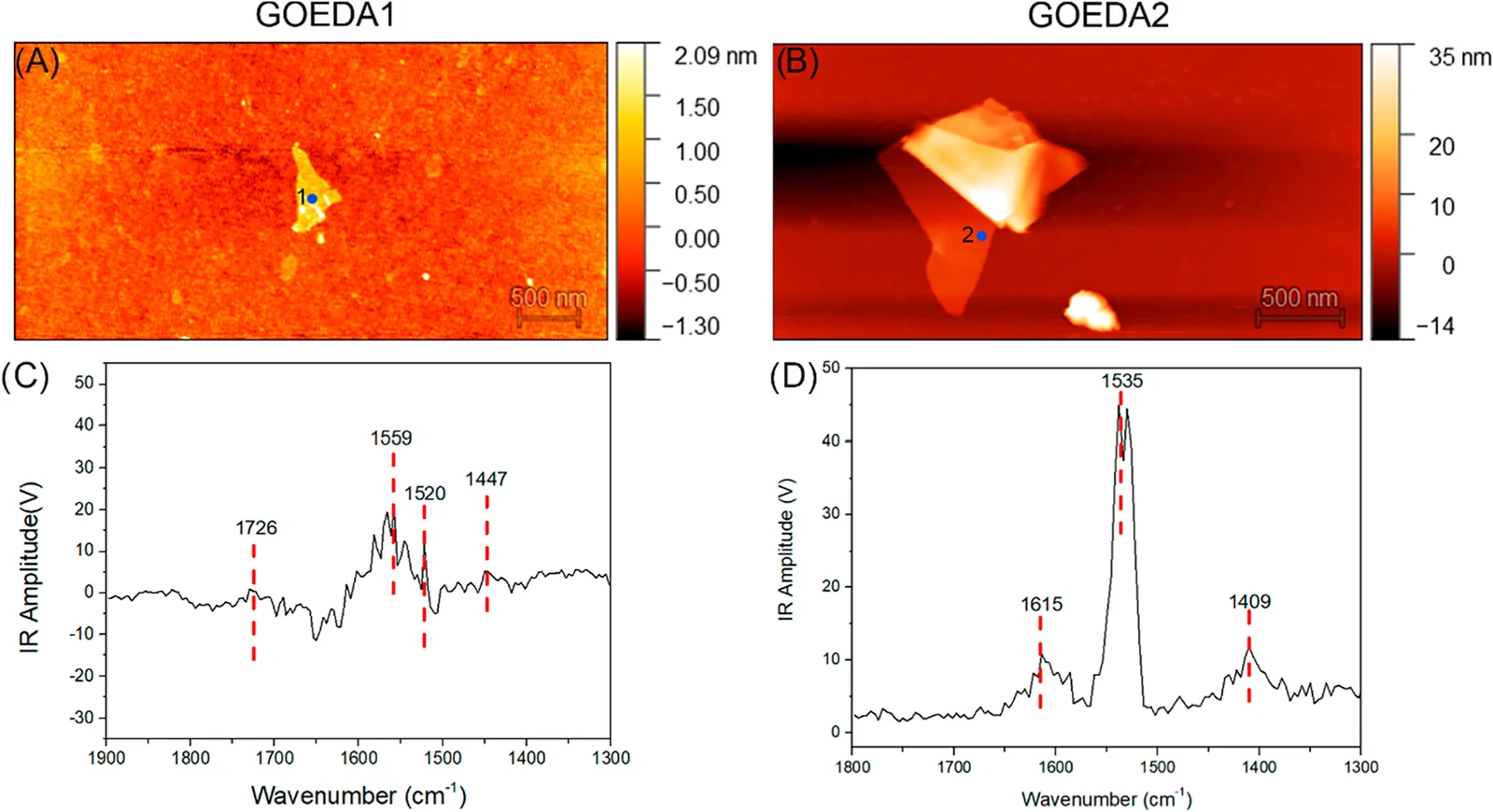
Topography maps obtained in SiO substrate for (A) GOEDA1 and (B) GOEDA2. (C) Single-point IR spectrum taken at point 1 for GOEDA1 and (D) point 2 for GOEDA2.

The topographic results obtained for the GOEDA1 sample (functionalized via epoxides) revealed smooth sheets with few folds, approximately 1 nm thick, characteristic of a two-dimensional layer of GOEDA1. The IR spectrum obtained at the center of the sheet (point 1) is presented in Figure 6C and in the SI. Bands at 3304 cm^–1^ (OH stretching), 3274 and 3256 (N–H stretching), and 2960 cm^–1^ (CH_2_ stretching) were observed (see SI). Additionally, bands at 1559 and 1520 cm^–1^, related to N–H bending, were identified (Figure 6C).^28,29^ The different positions of these bands suggest they may be associated with different types of amines. The band at 1559 cm^–1^ could correspond to the primary amine of anchored EDA, while the band at 1520 cm^–1^ may be attributed to a secondary amine.^28,29^ These results are consistent with those obtained by FTIR.

For the GOEDA2, large sheets with multiple folds were identified. Topographic analysis revealed a thickness around 5 nm, consistent with a few layers of GOEDA2. The infrared spectroscopy of GOEDA2 at point 2 identified three characteristic bands for the material (Figure 6D). The first band, at 1615 cm^–1^, can be attributed to the NC=O stretching of amides, which was not discernible in the bulk material’s FTIR results. A band at 1535 cm^–1^ was assigned to N–H bending, and another at 1409 cm^–1^ was attributed to C–N bending.^9,29^ These results align with those obtained by FTIR, despite slight wavelength shifts.

The AFM-IR maps obtained for GOEDA1 and GOEDA2 are shown in Figure 7. The mapping was performed at the wavenumber corresponding to the N–H bending vibration for each sample (see SI), which showed the strongest IR amplitude. For GOEDA1, the scan was conducted at 1512 cm^–1^, while for GOEDA2, it was at 1556 cm^–1^. This difference may be because the N–H bending falls within a range depending on the binding environment. The N–H bending for GOEDA2 on the sheets was 1535 cm^–1^, but at the edges it appeared to have shifted to 1556 cm^–1^. The edges absorbed strongly at this value. It is not possible to do a full spectrum at the very edge of the sheet to verify the shift in the peak. But we interpret this strong absorption at this particular value as stemming from a shift in the N–H bending absorption. Figure 7A,B displays the topographic maps, and Figure 7C,D shows the IR maps, with blue regions indicating no IR signal and red regions indicating the presence of a signal at the given wavenumber. For the GOEDA1 sample, the signal is distributed across the GO sheet, suggesting that the functionalization route via epoxides anchors the groups on the basal plane of GO. In contrast, for the GOEDA2 sample, the absorption agreeing with N–H bending was observed only at the edges of the sheets, consistent with the interpretation that the functionalization via carboxylic acids occurred mainly at the GO sheet edges.

**Figure 7:**
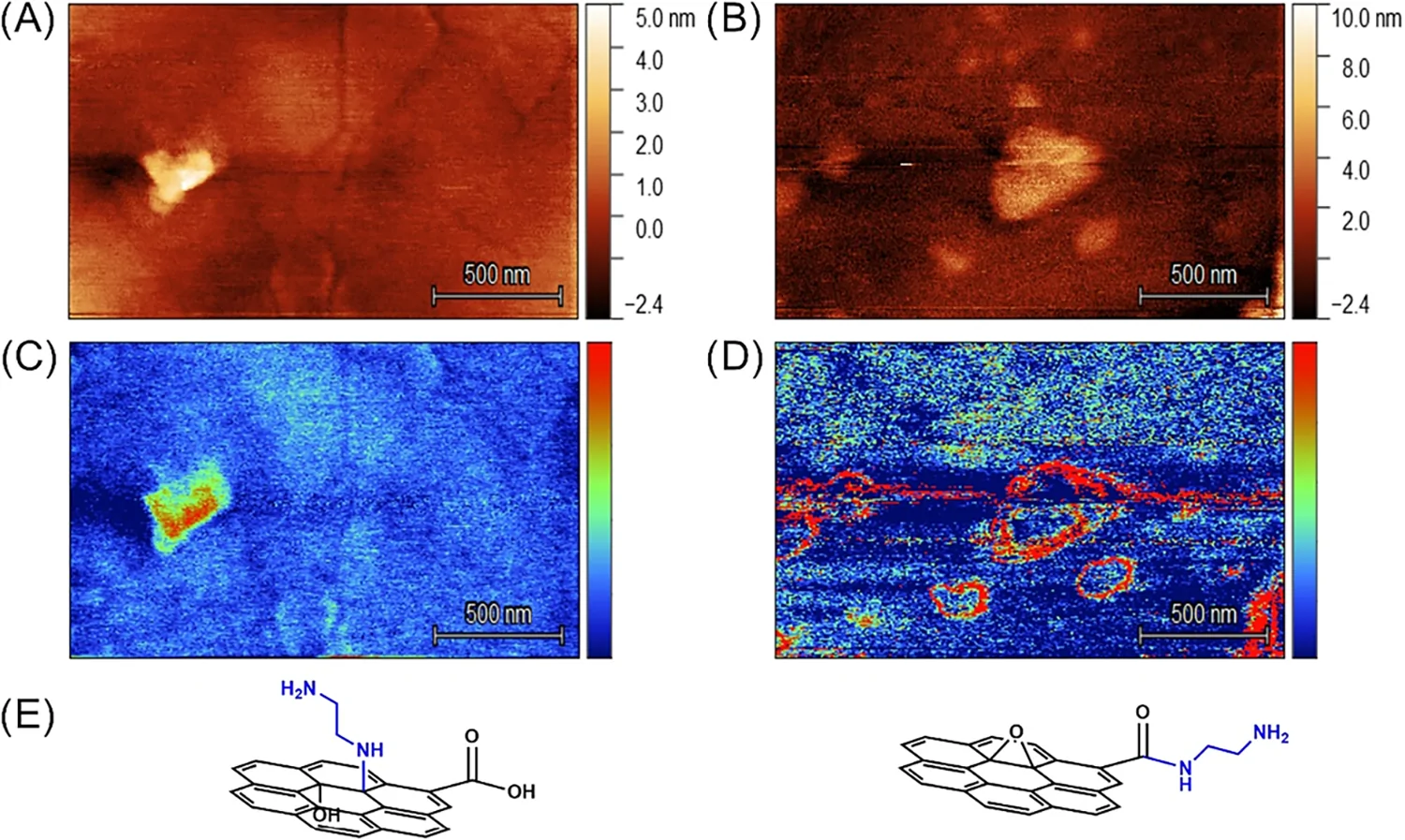
Topography maps obtained on freshly exposed gold of (A) GOEDA1 and (B) GOEDA2. AFM-IR absorption maps of (C) GOEDA1 at 1512 cm^–1^ and (D) GOEDA2 at 1556 cm^–1^. (E) Representative structures of GOEDA1 and GOEDA2, showing the different positioning of the groups.

### Catalytic Studies

The functionalized nanomaterials obtained were evaluated as nanocatalysts in the detoxification reaction of the organophosphate DEDNPP (Figure 8A). Following the formation of product 2,4-DNP, consecutive spectra were obtained (a typical one is shown in the inset in Figure 8B), and the kinetic profile at 400 nm for the product formation was used to calculate the pseudo-first-order rate constant (Figure 8B).^12^

**Figure 8:**
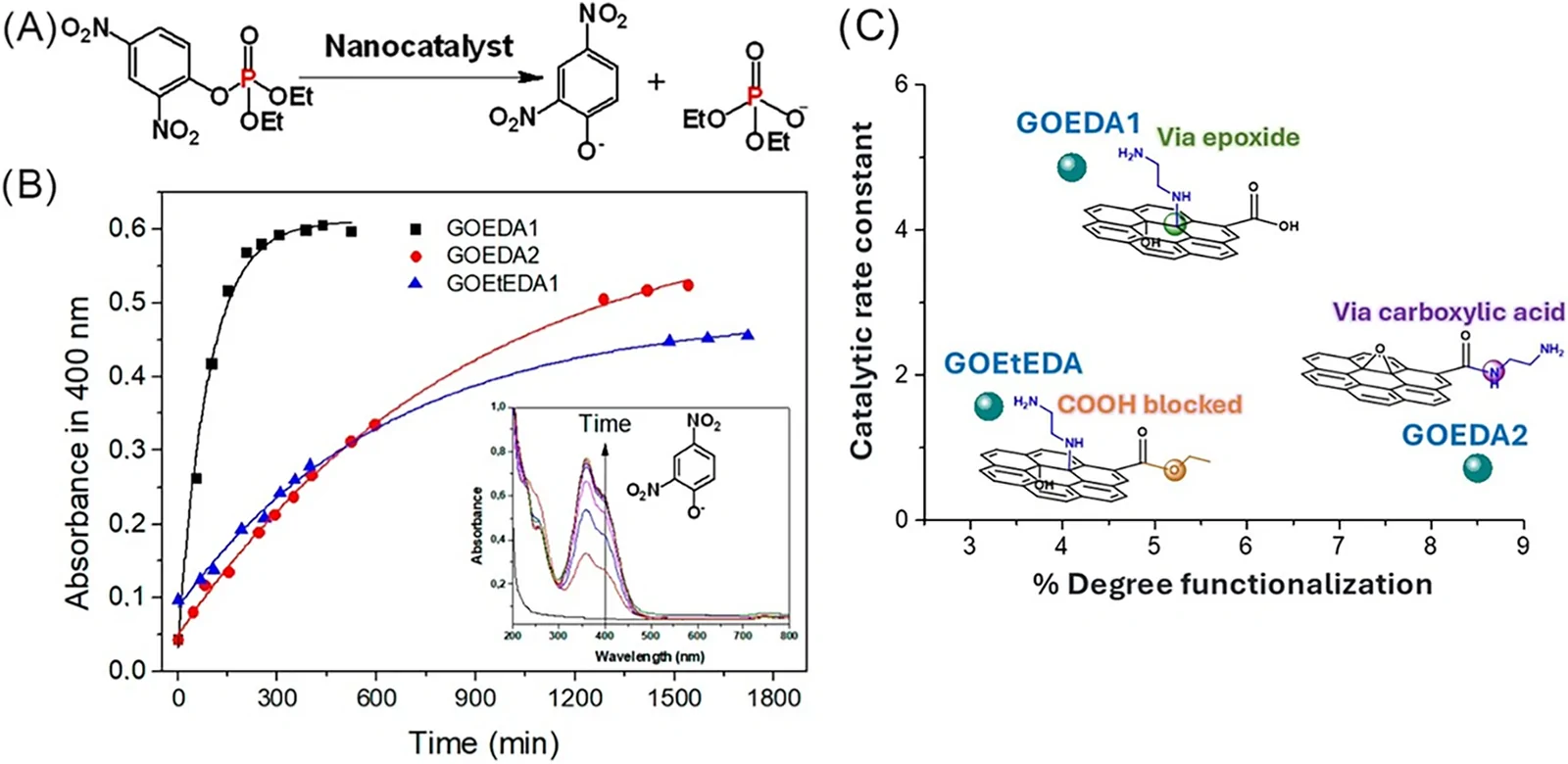
(A) Schematic representation for the neutralization reaction of DENDPP in the presence of the nanocatalyst. (B) Kinetic profiles at 400 nm for the neutralization reaction of DEDNPP with GOEDA1, GOEDA2, and GOEtEDA, at pH 8.5 and 25 °C. The solid lines are the pseudo-first-order fitting.^12^ The inset graph represents typical consecutive spectra obtained for the reactions. (C) Correlation between the catalytic rate constant (*k*_cat_, min^–1^ g^–1^) and the degree of functionalization estimated from CHNS analysis.

The rate constants obtained were 4.86 min^–1^ g^–1^ for GOEDA1, 0.721 min^–1^ g^–1^ for GOEDA2 and 1.57 min^–1^ g^–1^ for GOEtEDA. The value for GOEDA1 is one of the highest reported for this reaction under heterogeneous conditions (see Table 2) and shows that functionalizing via the epoxide site (GOEDA1), in contrast to the carboxylic acid (GOEDA2), is more efficient (nearly 7-fold higher) for catalytic applications. This cannot be attributed to a higher degree of functionalization, since GOEDA1 is less functionalized. It should be noted that many functionalization strategies usually focus on increasing the degree of functionalization.^38^ Figure 8C correlates the rate constant with the degree of functionalization estimated from CHNS (given as the total amount of nitrogen incorporated). Surprisingly, the least effective catalyst is GOEDA2, with the highest degree of functionalization. So, for the pursued catalytic application, this degree is not a key parameter. The hypothesis, based on previous studies,^8,9^ is that nonfunctionalized neighboring carboxylate groups can improve the catalytic behavior via a base catalysis. Therefore, the GO support of the nanocatalysts is not inert and can influence the catalytic outcome, justifying the synergistic effects observed for these materials. Since the reaction is conducted at pH 8.5 and given the p*K*_a_ values of the carboxylic acids ∼4–6 for GO, carboxylate is the predominant species^39^ in the reactions followed. The reactive species of the amines from EDA, anchored on the nanocatalysts, should be the neutral (NH -NH_2_) form,^40,41^ as can also be seen in the pH profile shown later in this study. Potentiometric titration, shown in the SI for GOEDA1, shows p*K*_a_’s of 6.88 and 8.87 for the amine sites (their conjugated acids). Therefore, at pH 8.5, a significant amount of the reactive neutral species is present. Actually, the study within the full pH range^7–11^ carried out for GOEDA1 agrees with this (discussed later and shown in the SI). Regarding the presence of carboxylates, in the case of GOEDA2, which was functionalized on the carboxylic acid sites, most of these species are not available, but for GOEDA1, where the functionalization was carried out on the epoxide sites, the carboxylates are still available. Carboxylates can act as general base catalysts via a hydrogen bond with the neighboring functionalized amine (EDA), increasing its nucleophilicity, thus improving its catalytic efficiency^42^ as shown in Figure 9. These carboxylates were blocked by an esterification with EtOH, which disables this base catalysis mechanism, although comprising the anchored amine groups on the epoxide sites: GOEtEDA (Figure 9). The rate constant of this blocked material was 3-fold lower that it́s analogue GOEDA1, despite them having similar nitrogen content (Figure 8C). Indeed, the nitrogen content is related to the reactive nucleophile that was anchored.

**Figure 9:**
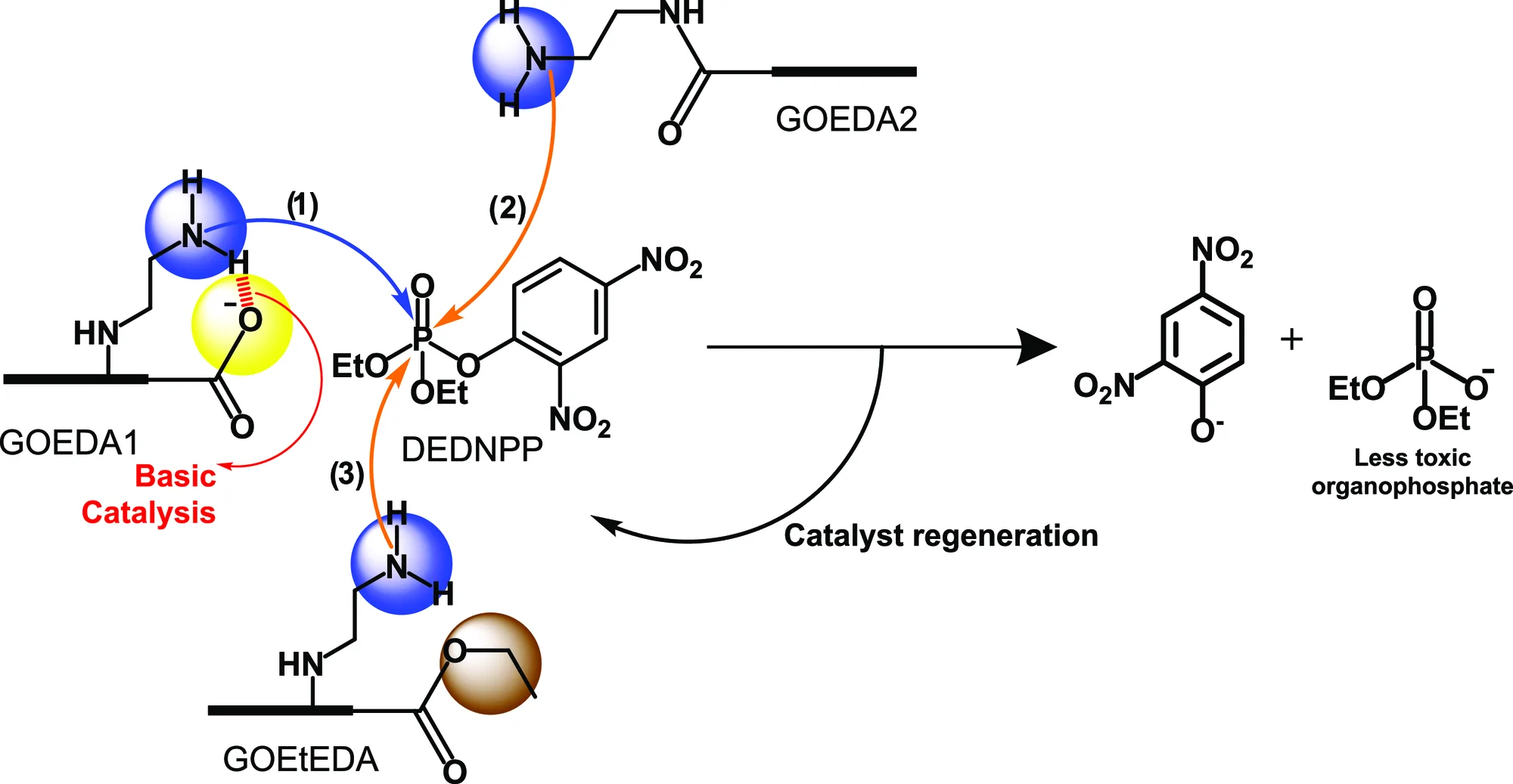
Representation of the proposed mechanisms for (1) the nucleophilic attack assisted by the basic catalysis in GOEDA1, (2) the nucleophilic attack of GOEDA2, and (3) the nucleophilic attack of GOEtEDA without the basic catalysis.

**Table 2:** Catalytic Rate Constants of Different Catalysts (from the Present Study and Compared with Others from the Literature) in the Neutralization Reaction of DEDNPP

	functionalization site of GO (when applicable)	temperature and pH	catalytic rate constant, min^–1^ g^–1^
GOEDA1 (this work)	Epoxide	pH 8.5 and 21 °C	4.86
GOEDA2 (this work)	Carboxylic acid	pH 8.5 and 21 °C	0.72
GOEtEDA (this work)	Epoxide and blocked acids	pH 8.5 and 21 °C	1.57
GO functionalized with cysteamine^9^	Epoxide	pH 8.5 and 21 °C	2.68
GO functionalized with cysteamine^8^	Carboxylic acids	pH 8.5 and 21 °C	0.53
GO functionalized with imidazole^9^	Carboxylic acid	pH 8.5 and 21 °C	0.54
GO bifunctionalized with cysteamine and API^9^	Epoxides and carboxylic acids	pH 8.5 and 21 °C	0.84
Poly(acrylicacid)-imidazole^26^	-	pH 9.0 and 25 °C	0.98
Poly(ethylene-*alt*-maleicanhydride)- imidazole^43^	-	pH 9.5 and 25 °C	4.59

Figure 9 represents the proposed mechanisms for the nanocatalysts evaluated in the reaction with DENDPP, all of which showed prominent catalytic behaviors. For the best catalyst, GOEDA1, a nucleophilic attack by the amine is assisted by general base catalysis of a neighboring carboxylate. For the analogue GOEtEDA (with blocked carboxylates), only the nucleophilic attack can occur. This is also the case for GOEDA2, which was functionalized with carboxylic acid. Overall, the nucleophilic attack is proposed to be responsible for the degradation of DEDNPP.^9^

Given the pronounced activity of GOEDA1, the pH effect was evaluated, and the pH rate profile (pH 7–11) is shown in the SI. The rate constant increases with the pH until reaching a plateau at ∼pH 8.5–10, which indicates that in this region the most reactive species of the amine anchored is the neutral reactive species. Then, the rate constant increases with the pH due to the contribution of alkaline hydrolysis and other hydroxyl species of GO. This is why a milder pH, such as 8.5, is preferable, which still has a significant contribution of the neutral species of amines, but is not greatly influenced by the alkaline hydrolysis.

Table 2 compares the results from the nanocatalysts designed herein with other catalysts from the literature for the same reaction (neutralization of DEDNPP^8,9,26,43^ ). For the GO-derived materials, it details at what site the functionalization occurred. Overall, analysis of Table 2 confirms that GOEDA1 is among the best catalysts and that functionalizing via the epoxide sites is more efficient, even when functionalizing different groups—such as amine (EDA) or cysteamine.^9^ For the first time, we were able to block the carboxylates, which agrees with the proposed intramolecular mechanism. Comparing the amine used herein (EDA) with other amines, for example, imidazole (both functionalizations targeted at the carboxylic acid sites), GOEDA2 shows a better performance.

Recycling studies were made with GOEDA1 for 5 consecutive reaction cycles with DEDNPP (see SI), and the results show no significant decay in the catalytic activity during those cycles, proving the material to be an efficient and reusable catalyst. Correlating this information with the mechanism proposed in Figure 9, we suggest that after the nucleophilic attack by the amine at the phosphorus center, a phosphorylated intermediate is formed, which is degraded and leads to the final degradation products and regenerates the initial catalyst.^9^ FTIR spectra and SEM images were taken before the first reaction and after the fifth one (shown in SI). The FTIR spectra show that the characteristic bands remained after all cycles, suggesting that no leaching occurred as well as that no new bands were observed. The SEM images show no significant difference between before and after the recycling study. In addition, SEM-EDS analysis did not find any evidence of phosphorus in the samples, indicating that no bonding or adsorption of DEDNPP or 2,4-DNP has occurred.

To address the selectivity in the reaction, usually the most common path occurs via the phosphorus atom, as shown in Figure 9. This is the path that is also preferable since it leads to less toxic products (effectively neutralizes DEDNPP). Another possible pathway is a nucleophilic attack at the aromatic carbon atom of DEDNPP, which can lead to several byproducts.^9^ To investigate this, we reacted GOEDA1 with CDNB, which allows nucleophilic attack only on the aromatic carbon (this is a known probe for this kind of reaction). The results are shown in SI and dońt evidence any reaction occurring, dismissing the unwanted pathway and confirming the selectivity of the nanocatalyst toward the phosphorus atom. A possible contribution of the adsorption of the fenol formed (2,4-DNP) was also investigated (see SI), but no significant adsorption occurred under 72 h, the maximum reaction time followed.

## Conclusion

This work establishes that the catalytic efficiency of GO is dictated more by the site of functionalization than by the overall degree of modification. EDA attachment at the epoxide sites preserved free carboxylic acids, enabling intramolecular catalysis and synergistic interactions that enhanced the degradation of the organophosphates. In contrast, blocking these acids diminished the activity, justifying their mechanistic importance. Overall, 3 different materials were obtained. Thorough characterization analyses were carried out by several techniques that agree with the selective positioning of the functionalization, which was confirmed by AFM-IR, where maps showed IR absorptions consistent with this picture.

Beyond demonstrating a practical route to improved nanocatalysts, our results highlight site-selective functionalization as a powerful tool for probing catalytic pathways. This dual role–functional diversification and mechanistic elucidation–provides a framework for rational nanodesign, where the interplay between functionalized and nonfunctionalized domains can be deliberately exploited.

By uncovering how positioning effects and synergistic interactions govern catalytic outcomes, this study advances the fundamental understanding of GO chemistry and points toward broader applications in environmental remediation, sensing, and beyond. The insights presented here suggest that the control over functionalization sites is not merely a synthetic choice but a decisive factor in unlocking the full potential of GO-based materials.

## Supplementary Material



## Notes

Complementary FTIR, TGA, CHNS, SEM, AFIM-IR, XPS, UV spectra, and kinetic studies of the pH profile and recycling studies, selectivity studies (PDF)
