# Supplementary material for: Selective Functionalization of Graphene Oxide Unlocks Synergistic Catalysis for Organophosphate Degradation
Source: ACS Omega. 2026 Oct 19;11(43):67196–207. doi: 10.1021/acsomega.6c07874 (PMC13737022; doi:10.1021/acsomega.6c07874)
Supplement: Supplementary file 1 [file ao6c07874_si_001.pdf]

Selective Functionalization of Graphene Oxide Unlocks Synergistic Catalysis for Organophosphate Degradation

Bernardo M. Régnier[a], Tue Hassenkam[b], Maria Luiza M. Rocco[c], Aldo J. G. Zarbin[a] and Elisa S. Orth*[a]

[a]Department of Chemistry, Universidade Federal do Paraná (UFPR), CP 19032, CEP 81531-980, Curitiba, PR, Brazil.

[b] *Globe Institute, University of Copenhagen, Oester Voldgade 5-7, Copenhagen, Denmark*

[c] *Instituto de Química, Universidade Federal do Rio de Janeiro (UFRJ), CEP 21941-909, Rio de Janeiro, RJ, Brazil*.

**Corresponding author.*

E-mail: elisaorth@ufpr.br; Tel: +55 41-33613184

SUPPLEMENTARY INFORMATION

**1. FTIR**	S3
**2. TGA**	S4
**3. CHNS elemental analysis**	S7
**4. SEM**	S8
**5. AFM-IR**	S11
**6. XPS**	S15
**7. Potentiometric Titration of GOEDA1**	S19
**8. UV spectra and kinetic studies of the pH profile**	S21
**9. UV spectra and kinetic studies of the Recycling studies**	S27
**10. Selectivity studies**	S33
**REFERENCES**	S36

1. FTIR of GOEt

Figure S1 shows the infrared spectrum of GO and GOEt. For GOEt, the CH_2 and hydroxyl bands are preserved, while the free OH bands decrease, likely due to the synthetic route[1–4]. In GOEt, ester formation is confirmed by the decrease of the carboxylic acid band at 1700 cm^{-1} and the new ester bands at 1200, 1098, and 1033 cm^{-1}, attributed to (C–O)OC and C–O vibrations[1,4], indicating successful acid blocking, thus esterification.

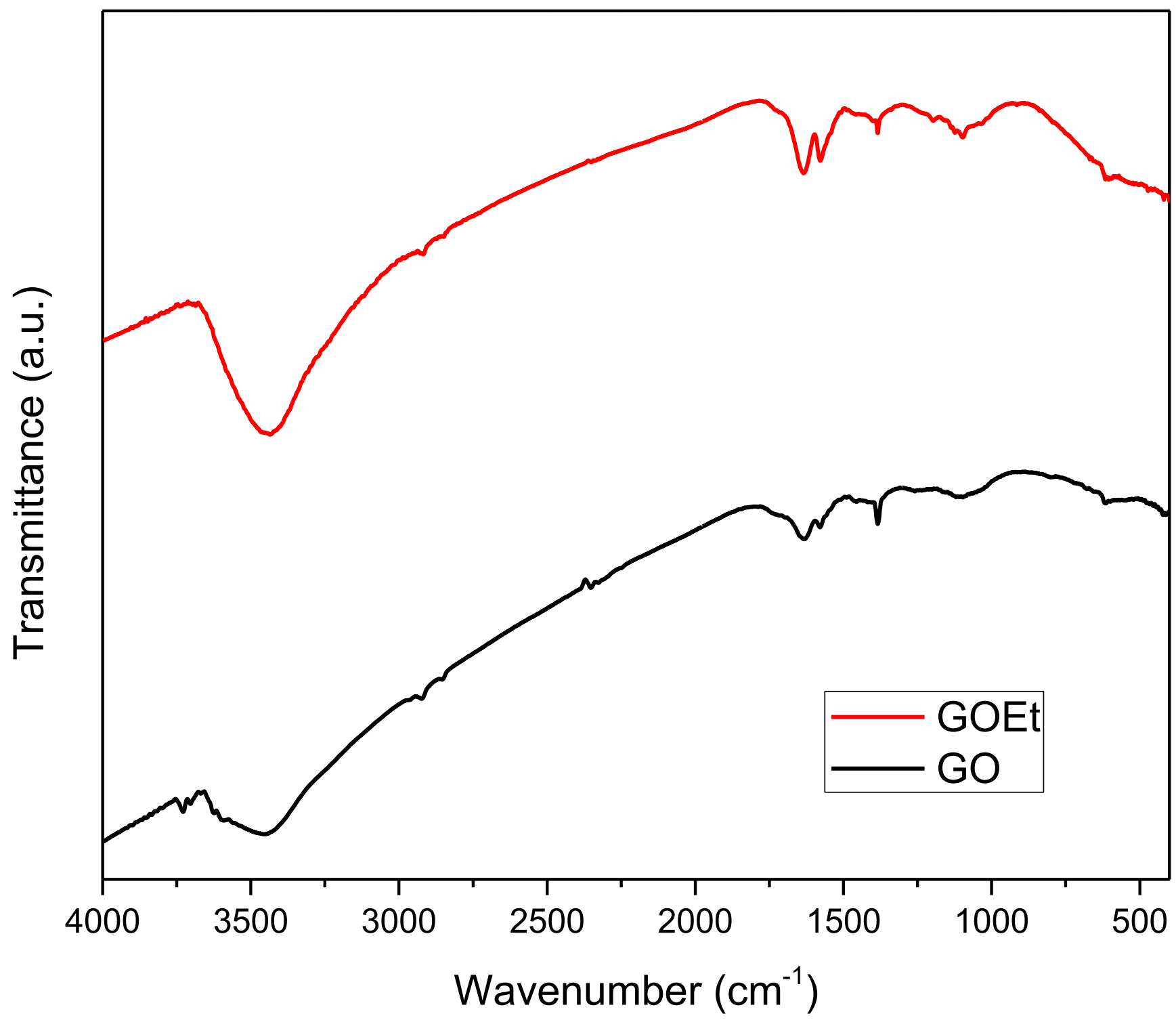

FIGURE S1. Infrared spectrum obtained for GO and GOEt.

2. TGA

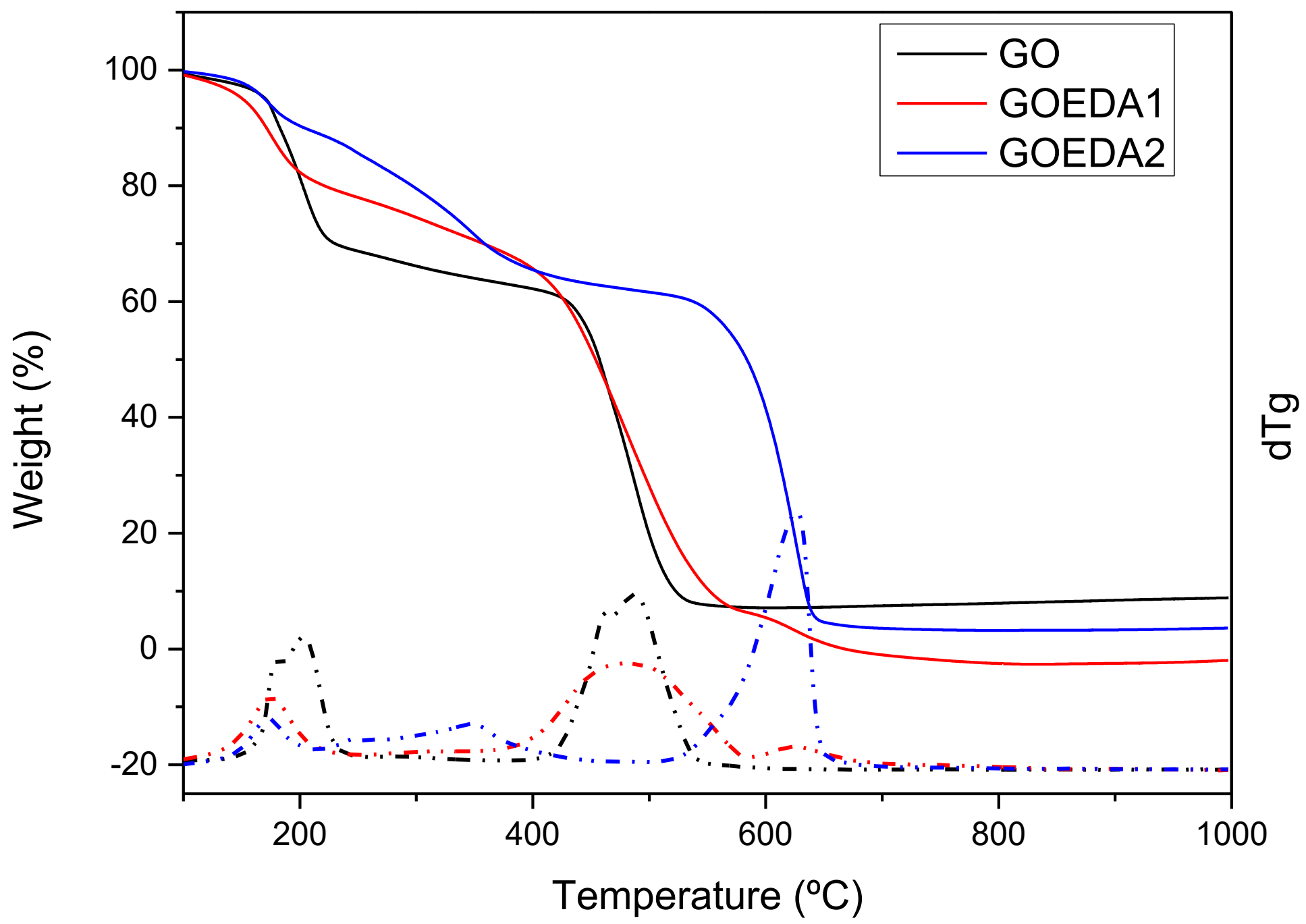

FIGURE S2. TGA and dTg obtained for GO, GOEDA1 and GOEDA2.

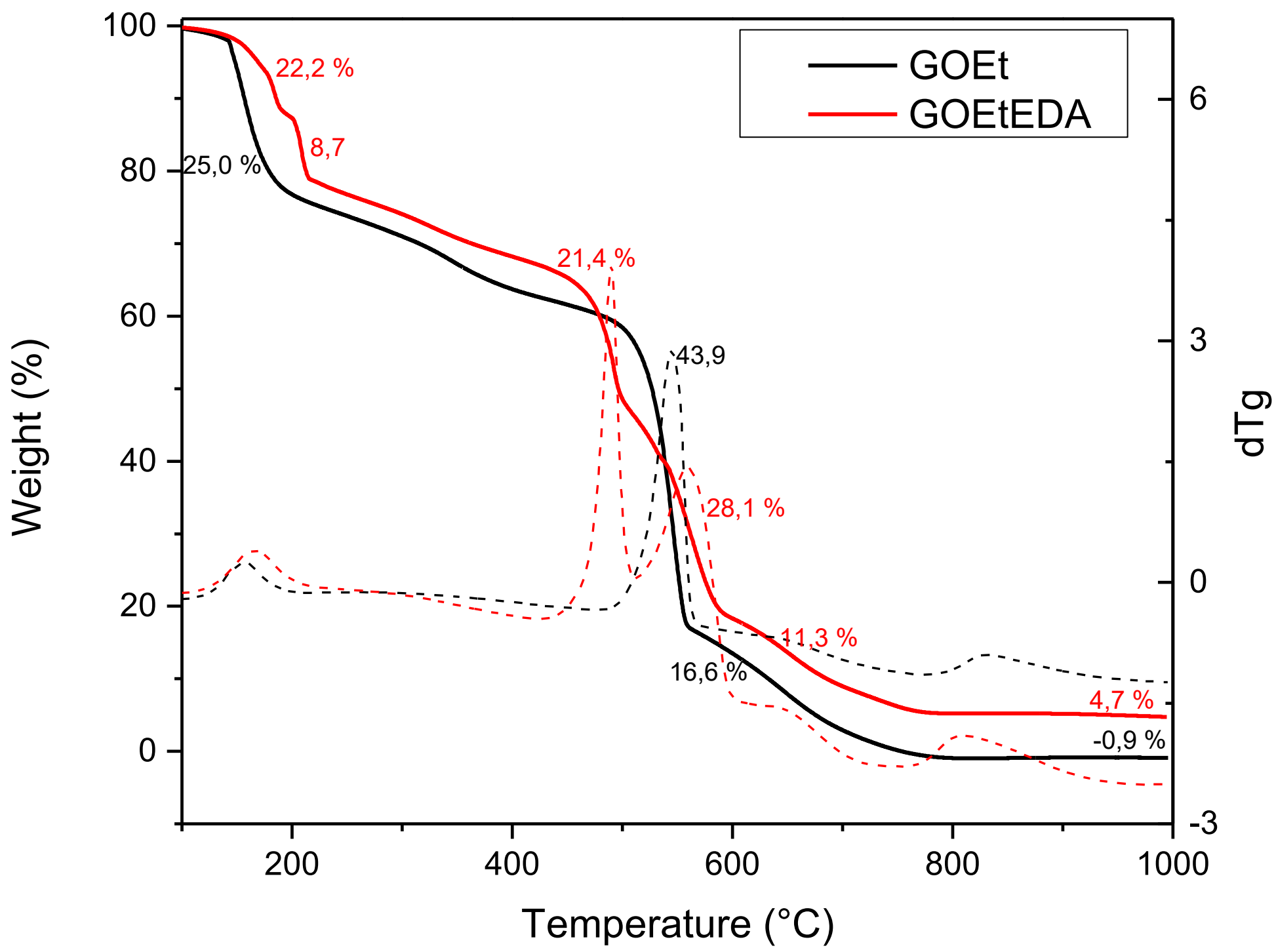

FIGURE S3. TGA and dTg obtained for GOEt and GOEtEDA.

For the sample GOEt, the mass loss event attributed to oxygenated groups at 165 °C showed a significant loss of 25.0%. The next mass loss event occurred at 344 °C with a 13.8% loss[2,3]. In the region corresponding to the carbonaceous blackbone degradation, GOEt exhibited similar behavior, with a weight loss near 538 °C, indicating similar thermal stability compared to GOEDA1Et, which start to degrade around 490 °C.

TABLE S1 – Comparison of temperature ranges of weight loss events and percentage lost for GO and monofunctionalized materials.

	**Material**			**Assignment**
	**GO**	**GOEDA1**	**GOEDA2**	
**T (°C) / Perda de massa (%)**	**129-242 / 29.9**	**92-246 / 21.8**	**92-211 / 9.5**	Oxygenated group
	**-**	**-**	**273-480 / 26.5**	Functionalization and others oxygenated groups
	**390-603 / 54.4**	**362-582 / 63.2**	**480-699 / 62.7**	GO blackbone
	**-**	**582-700 / 7.4**	**-**	Csp^2 network

TABLE S2 - Comparison of temperature ranges of weight loss events and percentage lost for blocked acid materials.

	**Material**		**Assignment**
	**GOEt**	**GOEtEDA**	
	**108-202 / 25.0**	**102-211 / 21.5**	Oxygenated group

T (°C) / Weight loss (%)	215-474 / 13.8	211-421 / 11.34	Functionalization and others oxygenated groups
	474-574 / 43.9	421-608 / 22.1	GO blackbone
	574-774 / 16.6	608-733 / 12.2	Csp^2 network

3. CHNS analysis

Table 3 presents the elementary analysis of CHNS in each material-the residue values were obtained through TGA.

Table S3. Percentage of C, H, N, S and O obtained in each material

Sample	C (%)	H (%)	N (%)	S (%)	Residue	O (%)
GO	45±2.0	2.6±0.2	0.42±0.04	0.97±0.05	8.6	42±2
GOEDA1	49.9±0.3	2.9±0.2	4.1±0.2	0.27±0.04	0	42±1
GOEDA2	55.7±0.2	3.9±0.1	8.5±0.2	0.34±0.6	3.4	28±1
GOEtEDA1	57±0.2	2.7±0.1	3.2±0.02	0.48±0.04	4.7	31.5±0.1
GOEDA1Et	56.3±0.1	2.5±0.2	4.0±0.01	0.42±0.02	18.9	18.4±0.2

4. SEM images

Figures S4 to S6 combine the MEV imagens obtained for GO (Figure S4), GOEDA1 (Figure S5) and GOEDA2 (Figure S6), in different amplifications.

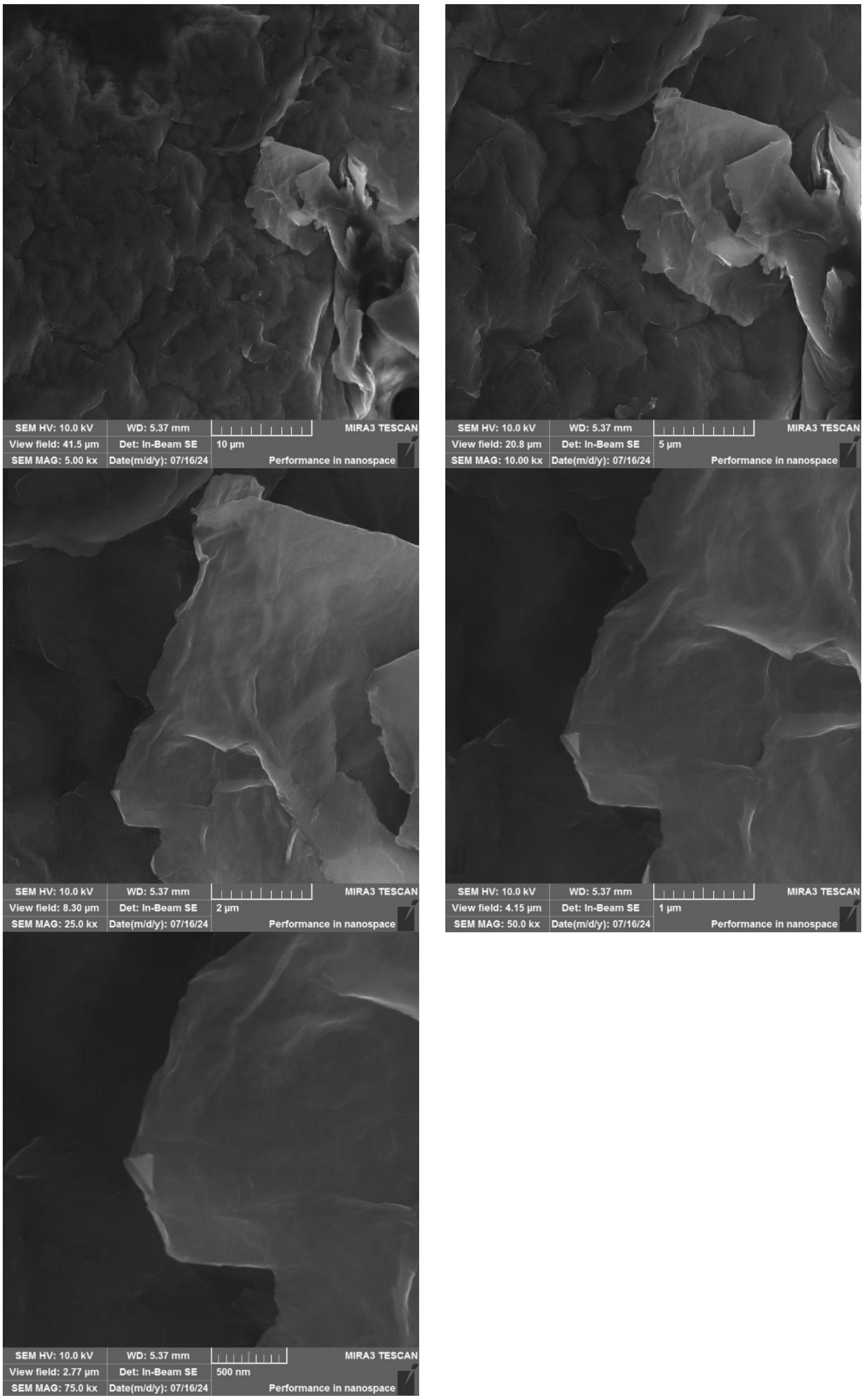

FIGURE S4 - SEM IMAGES OF GO AT VARIOUS AMPLIFICATIONS.

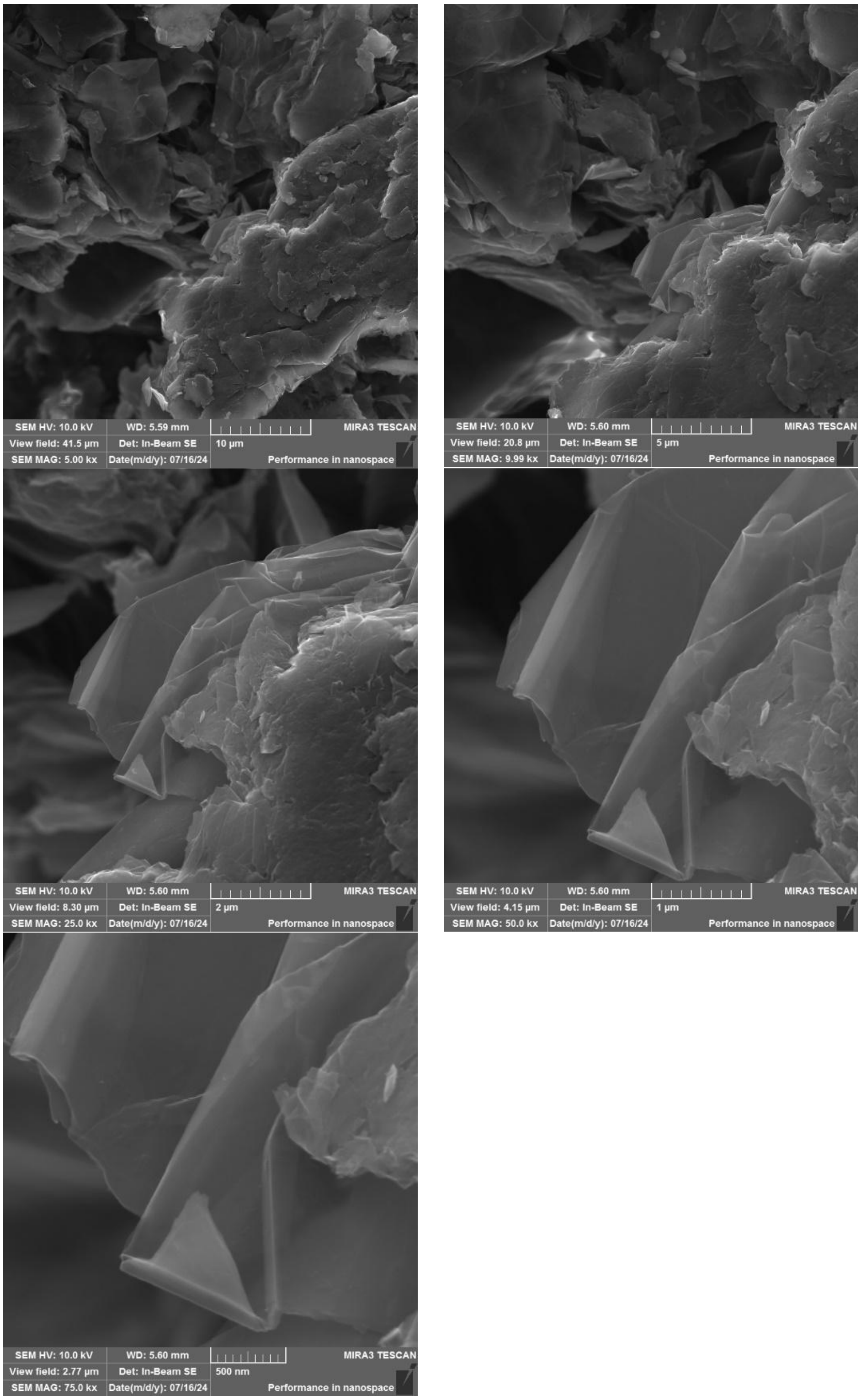

FIGURE S5 - SEM IMAGES OF GOEDA1 AT VARIOUS AMPLIFICATIONS.

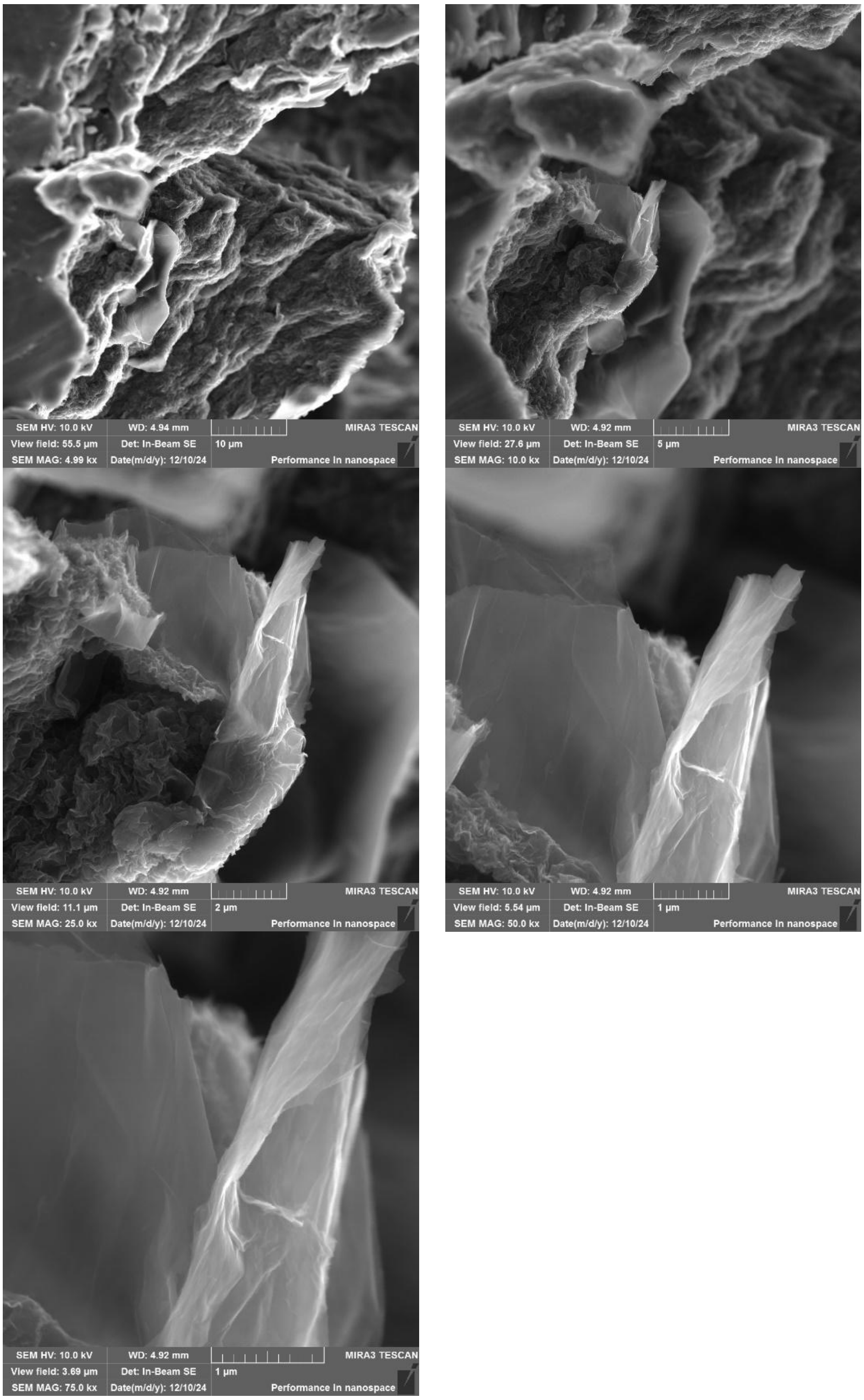

FIGURE S6 - SEM IMAGES OF GOEDA2 AT VARIOUS AMPLIFICATIONS.

5. AFM-IR

Figures S7 and S8 show the topography maps obtained for GOEDA1 and GOEDA2, alongside the topography profile and punctual IR spectra. Figure S9 shows the topography spectra from the IR-map analysis and the specific spots IR spectra used to determine the wavelength we used to produce the IR map. Figure S10 captures maps of the same GOEDA1 sheet in different wavelengths, showing signal only in an amine related wavelength.

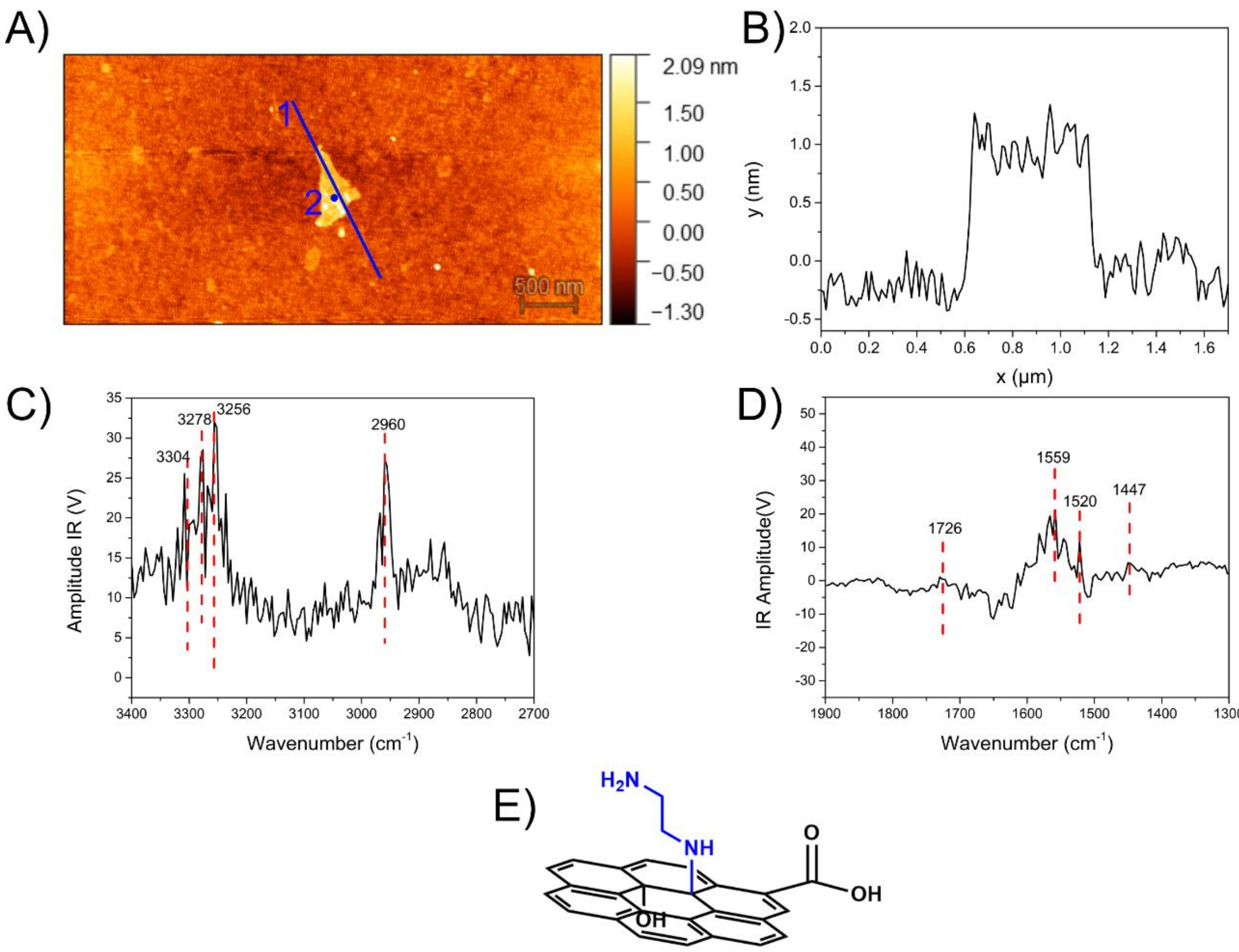

FIGURE S7 – A) topography map of GOEDA1 in SiO_2 substrate. B) Topography profile corresponding to line 1. Specific spot IR spectra taken in point 2, C) from 3400 to 2700 cm^{-1} and D) from 1900 to 1300 cm^{-1}. E) GOEDA1 simplified structure.

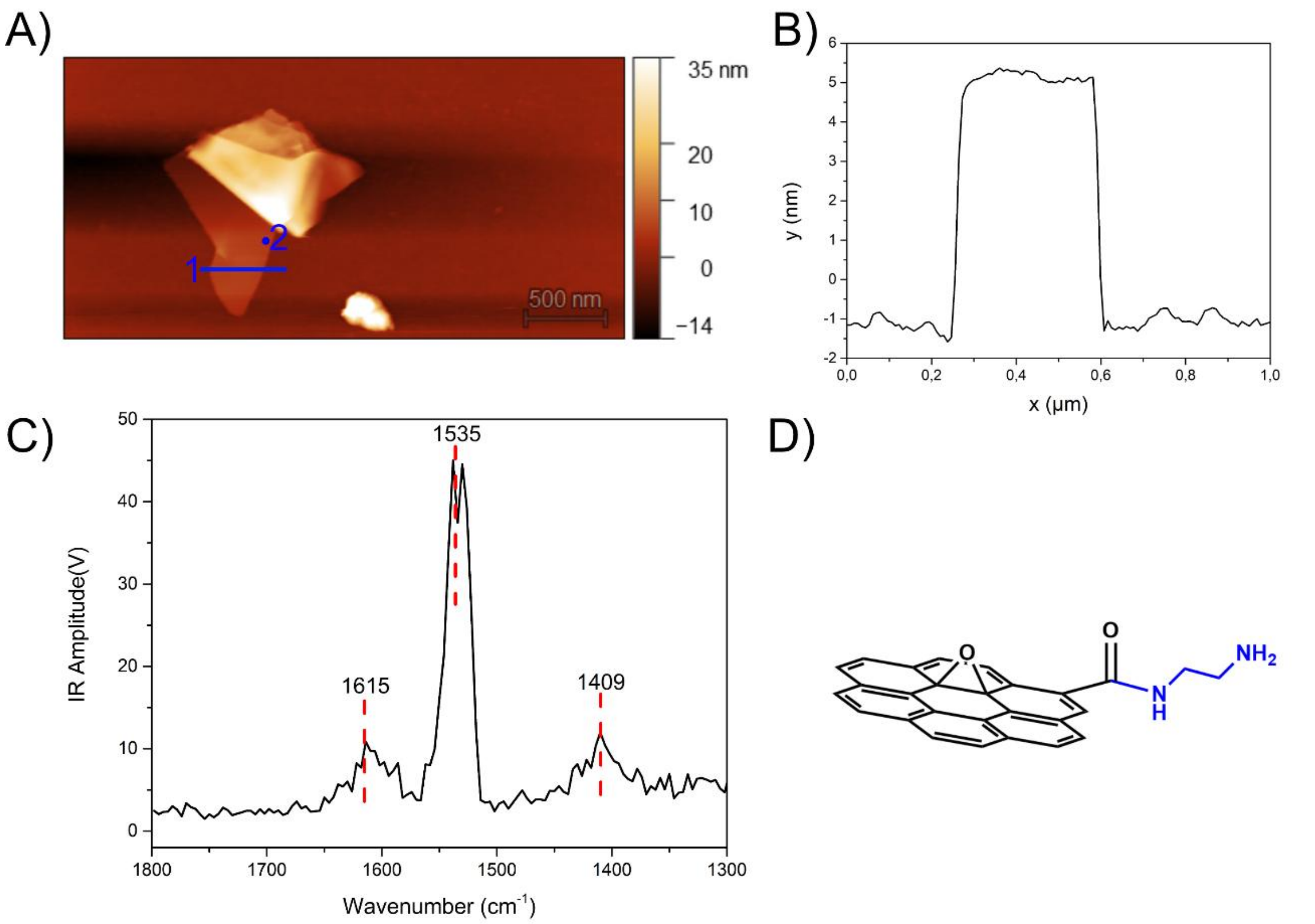

FIGURE S8 - A) Topography map of GOEDA2 in SiO_2 substrate. B) Topography profile corresponding to line 1. Specific spot IR spectra taken in point 2, C) from 1800 to 1300 cm^{-1}. D) GOEDA2 simplified structure.

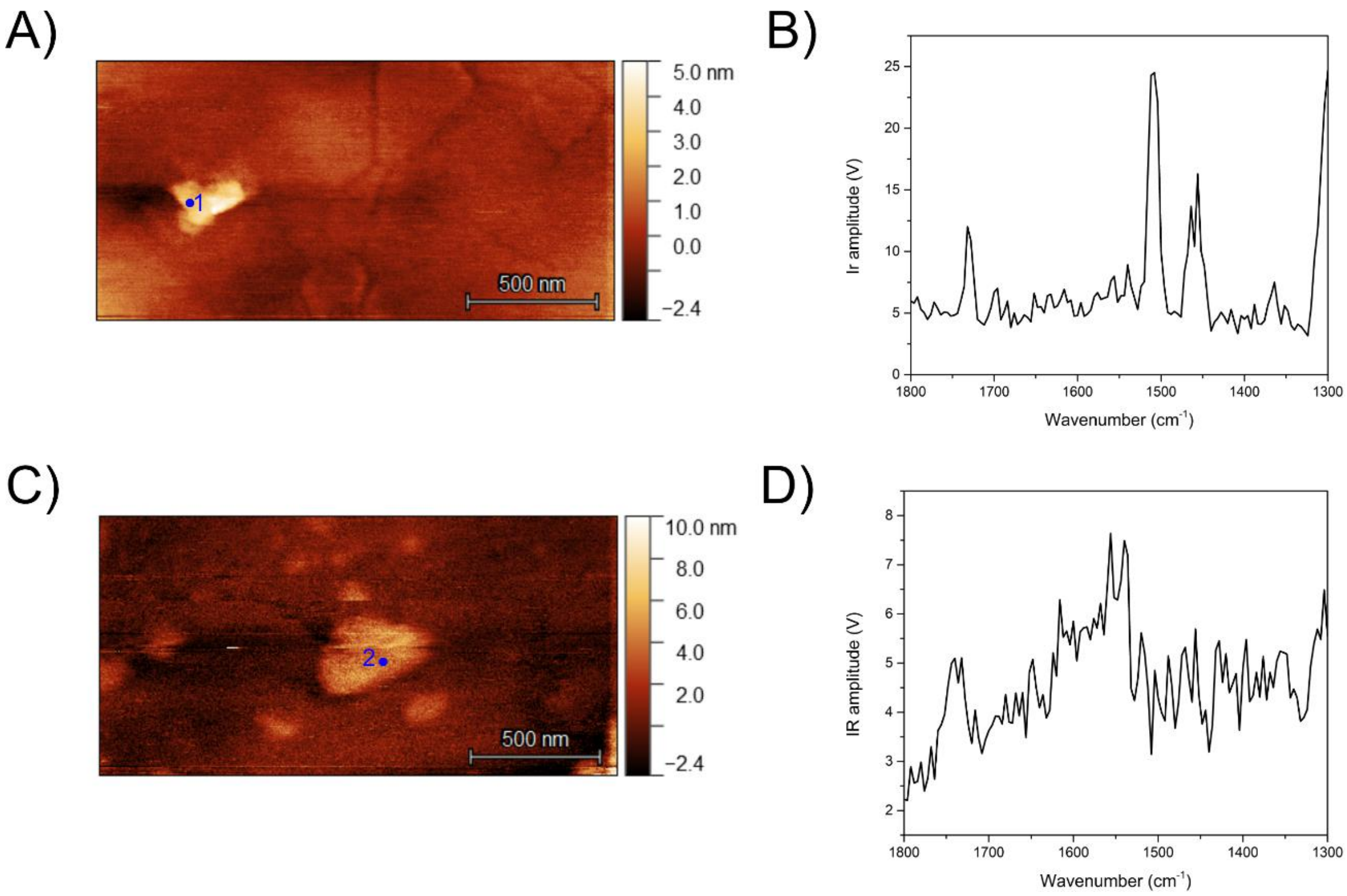

FIGURE S9 – A) Topography map of GOEDA1 in gold substrate B) IR spectra taken in point 1. C) Topography map of GOEDA2 in gold substrate D) IR spectra taken in point 2.

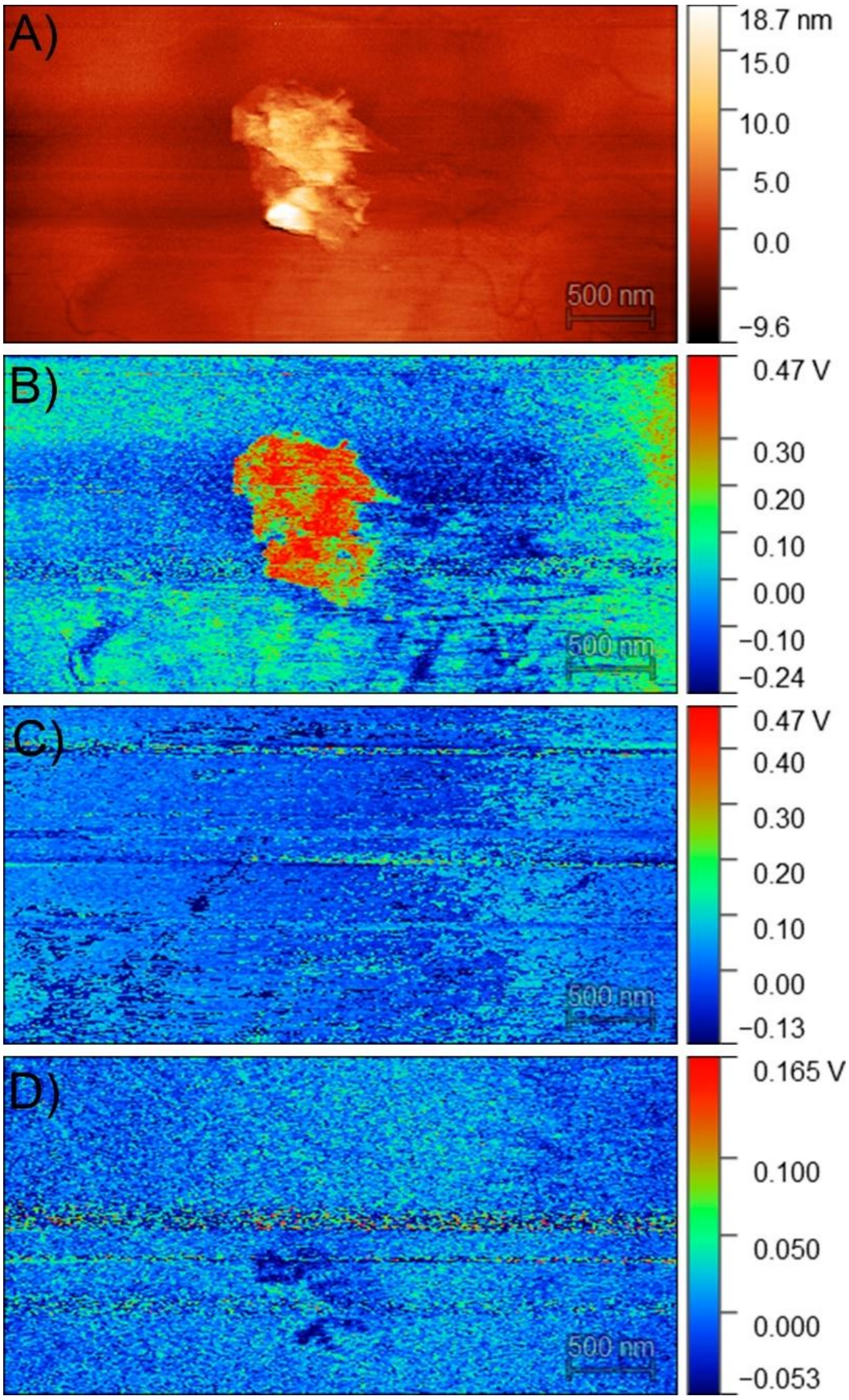

FIGURE S10 – A) Topographic map of goeda1. map obtained at the wavenumbers b) 1512, c) 1590 and d) 2000 cm^{-1}.

6. XPS

Here are presented the full XPS survey spectra of GO, GOEDA1 and GOEDA2 (FIGURE S11), alongside the N1s high resolution spectra of GOEDA1 (FIGURE S12) and GOEDA2 (FIGURE S13). Tables S4 to S5 present the assignment made for the bands in each material based on their spectra[3,5].

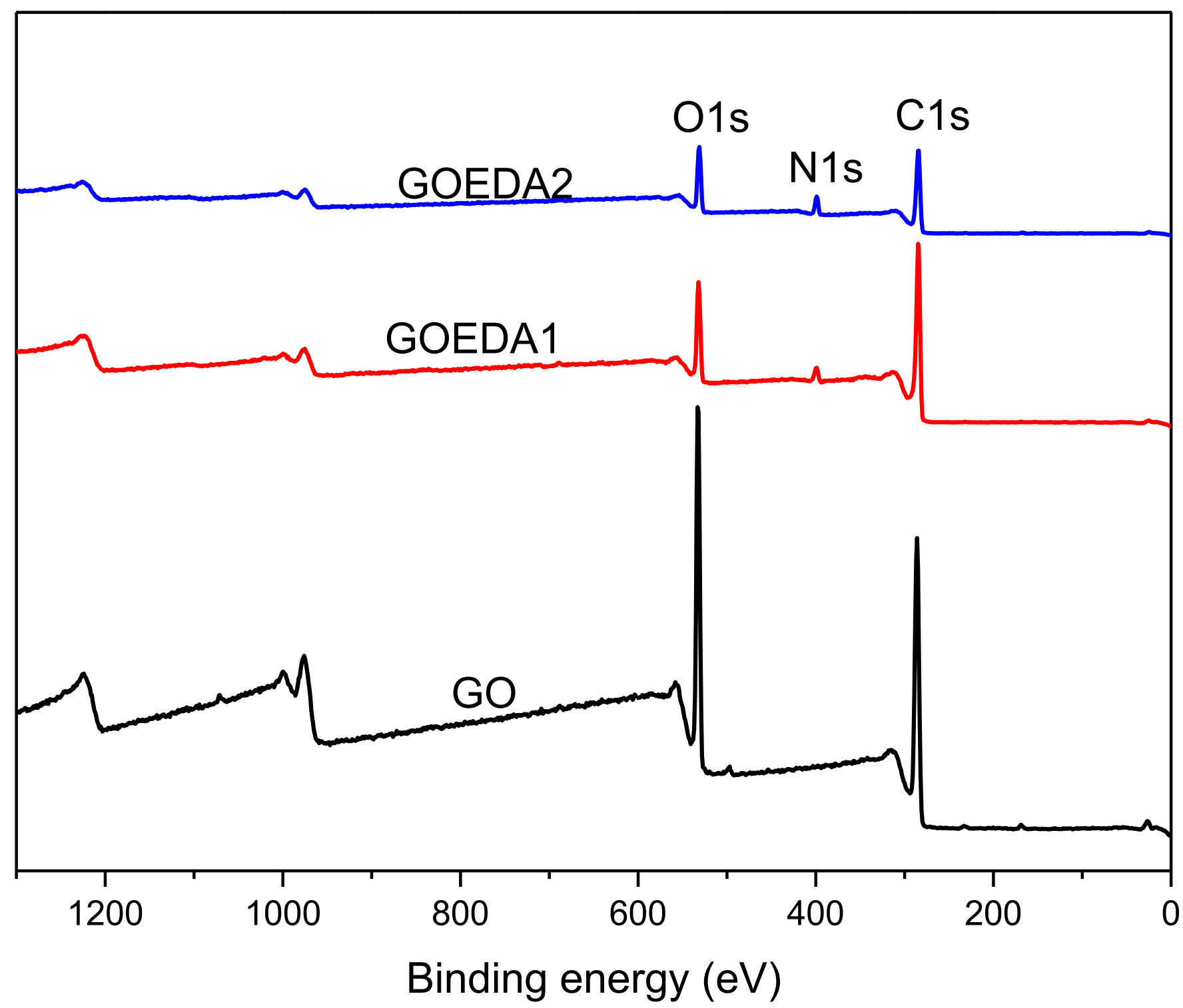

FIGURE S11 – Survey XPS spectra of GO, GOEDA1 and GOEDA2.

Table S4. Assignments of GO from high resolution XPS[3,5].

	Functional groups	Organic function	eV	%
Carbon	C=C	Hydrocarbon	283.3	2.2
	C-C		284.5	32.4
	C-O	Epoxide/hydroxyl	286.4	46.5
	C=O	Ketone/Quinone	288.2	17.3
	COOH	Carboxylic acid	289.9	1.6
Oxygen	C(=O*)OC	Quinone	530.8	3.9
	C=O	ketone	531.9	18.0
	C-OH/C-O-C	Epoxide/hydroxyl	532.6	56.0
	O*-(C=O)-C	Carboxylic acid	533.8	18.3
	H_2O (adsorbed)	-	534.7	3.7

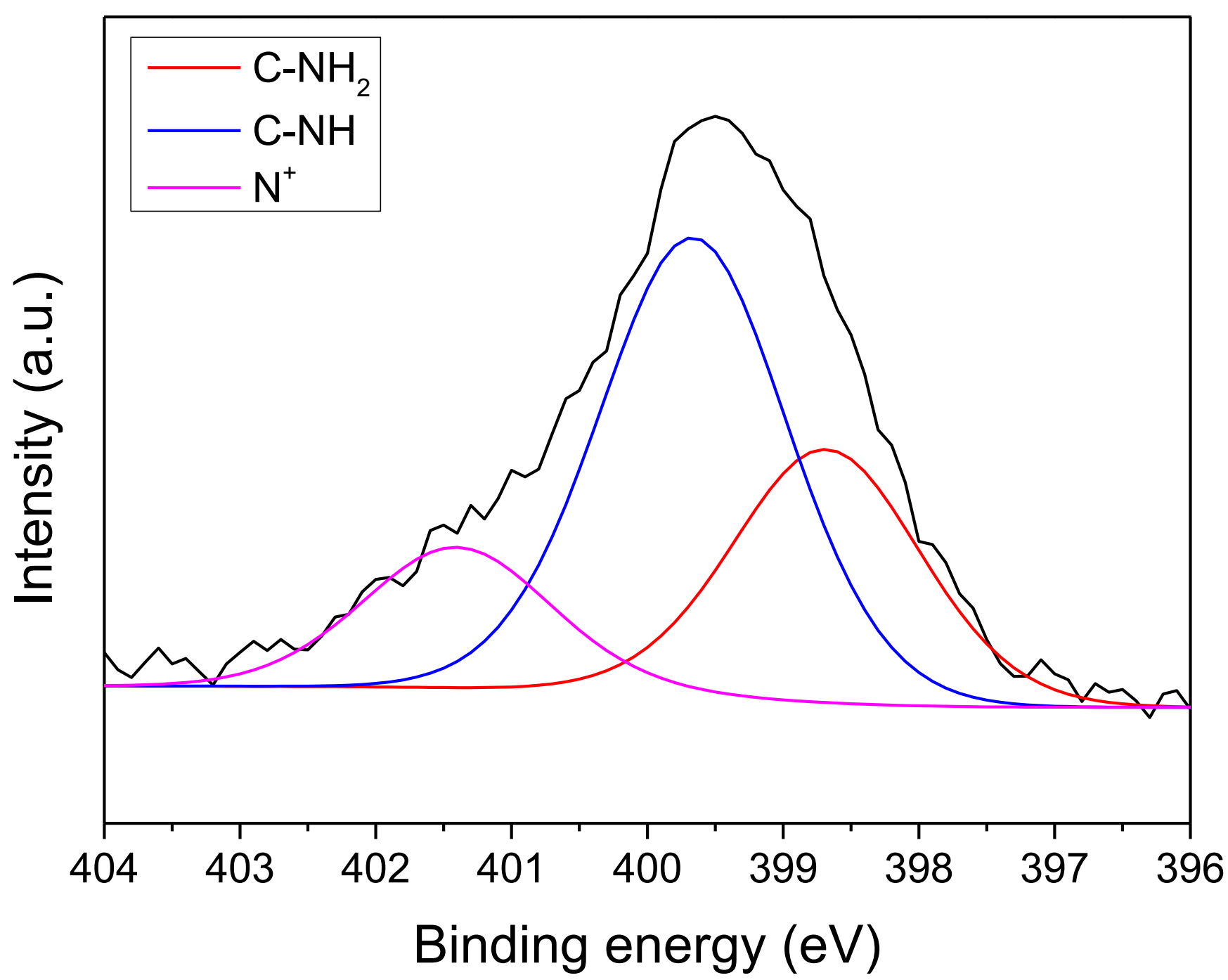

FIGURE S12 - High resolution N1s XPS spectrum of GOEDA1.

Table S5. Assignments of GOEDA1 from high resolution XPS[3,5].

	Functional groups	Organic function	eV	%
Carbon	Amorphous carbon	Hydrocarbon	283,0	1,73
	C=C		284,0	49,71
	C-C		284,7	15.5
	C-NH2	Amine	285,9	18.6
	C-O	Epoxide/hydroxyl	286,8	6.5
	C=O	Ketone/Quinone	288,0	4.3
	COOH	Carboxylic acid	289,4	2.1
Oxygen	C(=O*)OC	Quinone	530,3	11.8
	C=O	Ketone	531,3	29.0
	C-OH/C-O-C	Epoxide/hydroxyl	532,3	45.7
	O*-(C=O)-C	Carboxylic acid	533,4	11.5
	H_2O adsorbed	-	535,0	2.1
Nitrogen	C-NH2	Primary amine	398,7	29.7
	C-NH	Secondary amine	399,7	53.7
	N+	Deprotonate amine	401,4	16.5

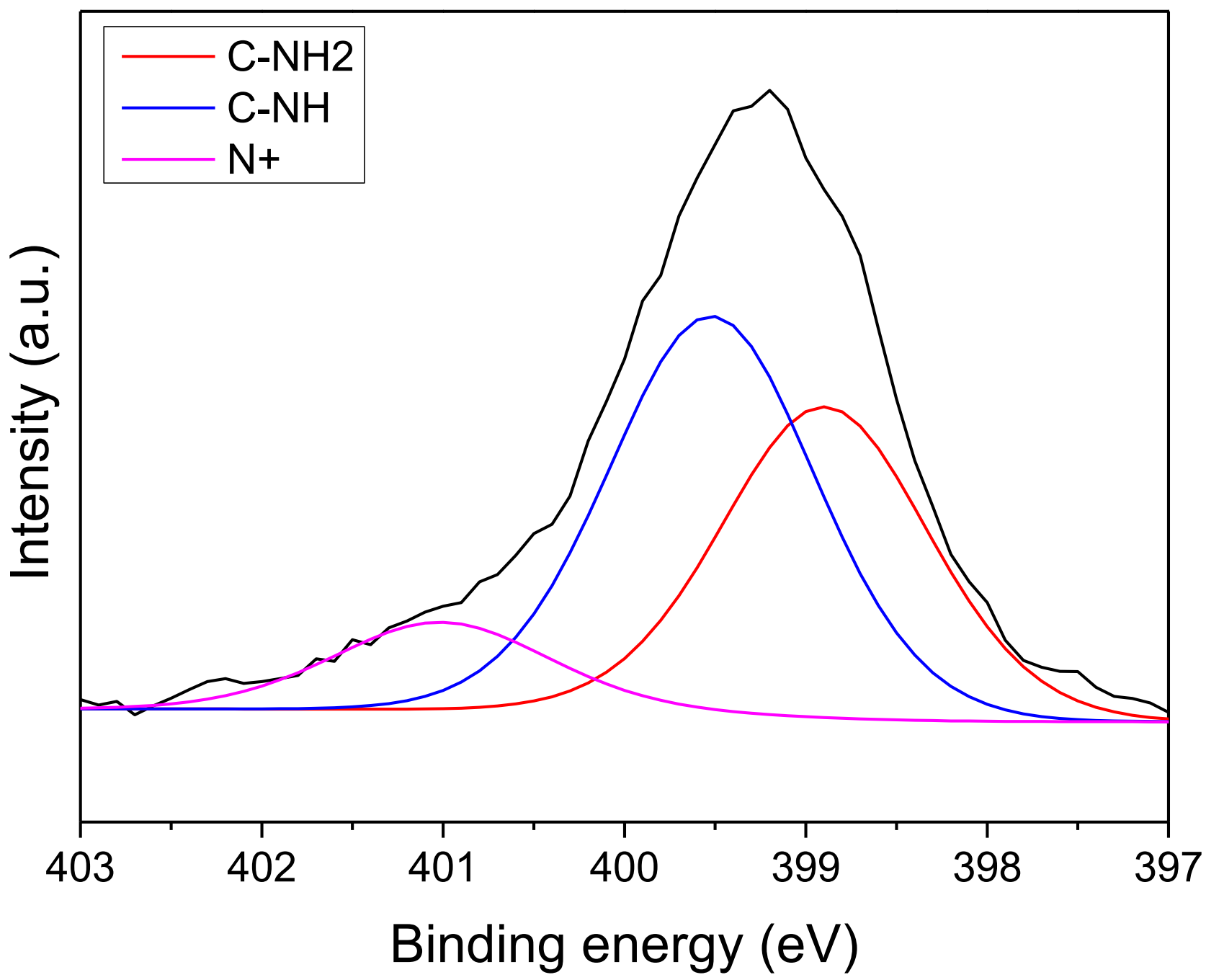

FIGURE S13 – High resolution N1s XPS spectrum of GOEDA2.

Table S6 - Assignments of GOEDA2 from high resolution XPS[3,5].

	Functional Group	Organic function	eV	%
Carbon	C=C	Hydrocarbon	284,0	47.5
	C-C		285,0	16.8
	C-NH2	Amine	285,9	20.1
	C-O	Epoxide/hydroxyl	286,9	6.9
	C=O/C=O-N	Ketone/Amide	287,7	7.4
	COOH	Carboxylic acid	288,9	1.4
Oxygen	C(=O*)OC	Quinone	530,2	9.7
	C=O/N-C=O	Ketone/amide	531,0	39.3
	C-OH/C-O-C	Epoxide/hydroxyl	532,0	38.5
	O*-(C=O)-C	Carboxylic acid	533,0	11.5
Nitrogen	N-C=O	Amide	398,9	38.9
	NH2	Amine	399,5	49.8
	N+	Deprotonate amine	401,0	11.3

7. Potentiometric Titration of GOEDA1

The potentiometric titrations were performed using approximately 3 mg of the material in powder in 25 mL of ultrapure water. The medium was maintained at 25 °C using a thermostatic bath. The pH was initially adjusted to approximately 3.9 using HCl (1 mol/L), and pH increments were achieved and measured under stirring, by controlled additions of standardized KOH. The potentiometric titration of GOEDA1 is presented on figure S14, with the proposed equilibrium in Figure S15 and the calculated pK_a values on table S6. The pK_a was calculated using the BEST7 software[6,7].

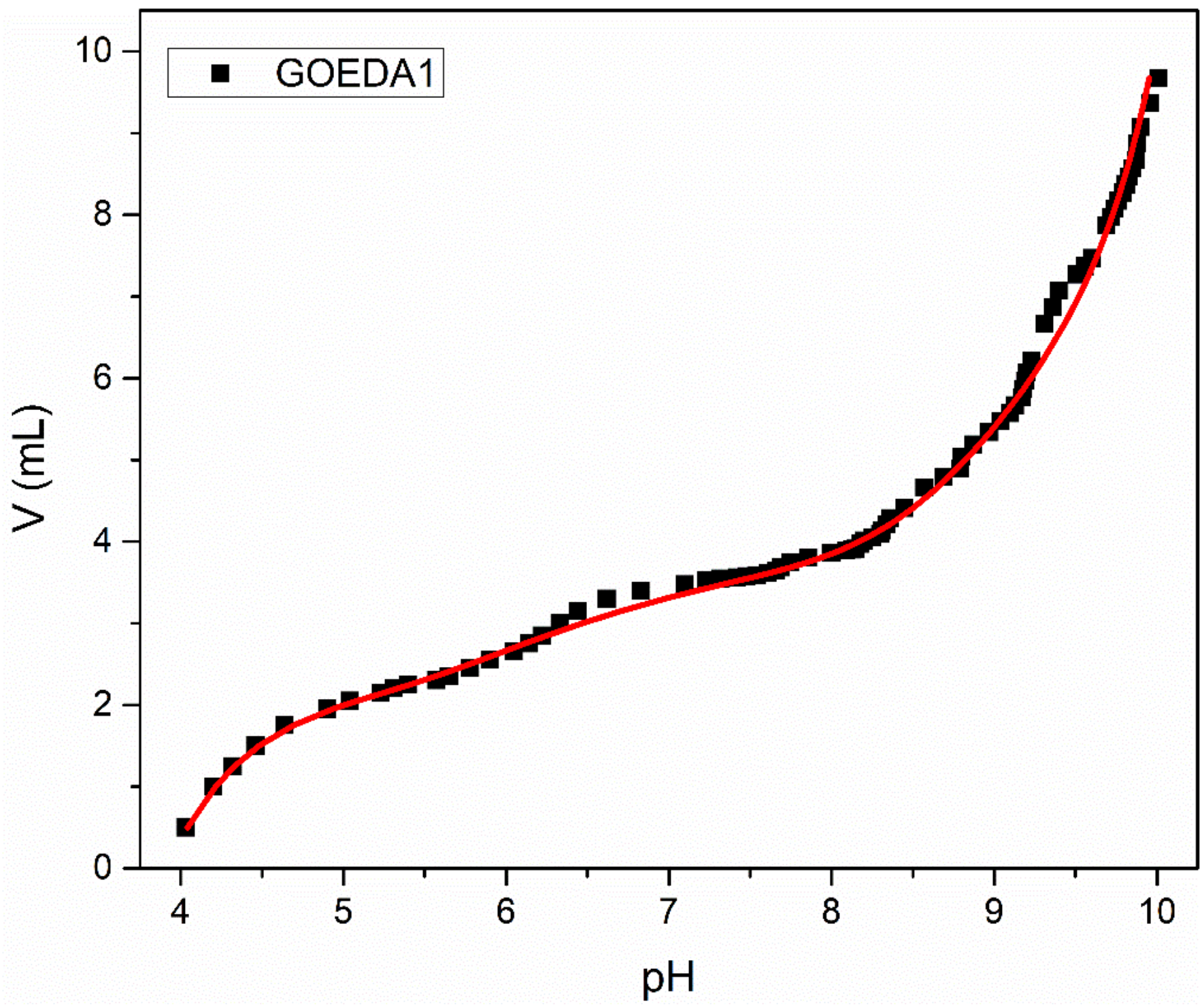

FIGURE S14 – Potentiometric titration of GOEDA1 at °25C. The red line corresponds to the calculated curve.

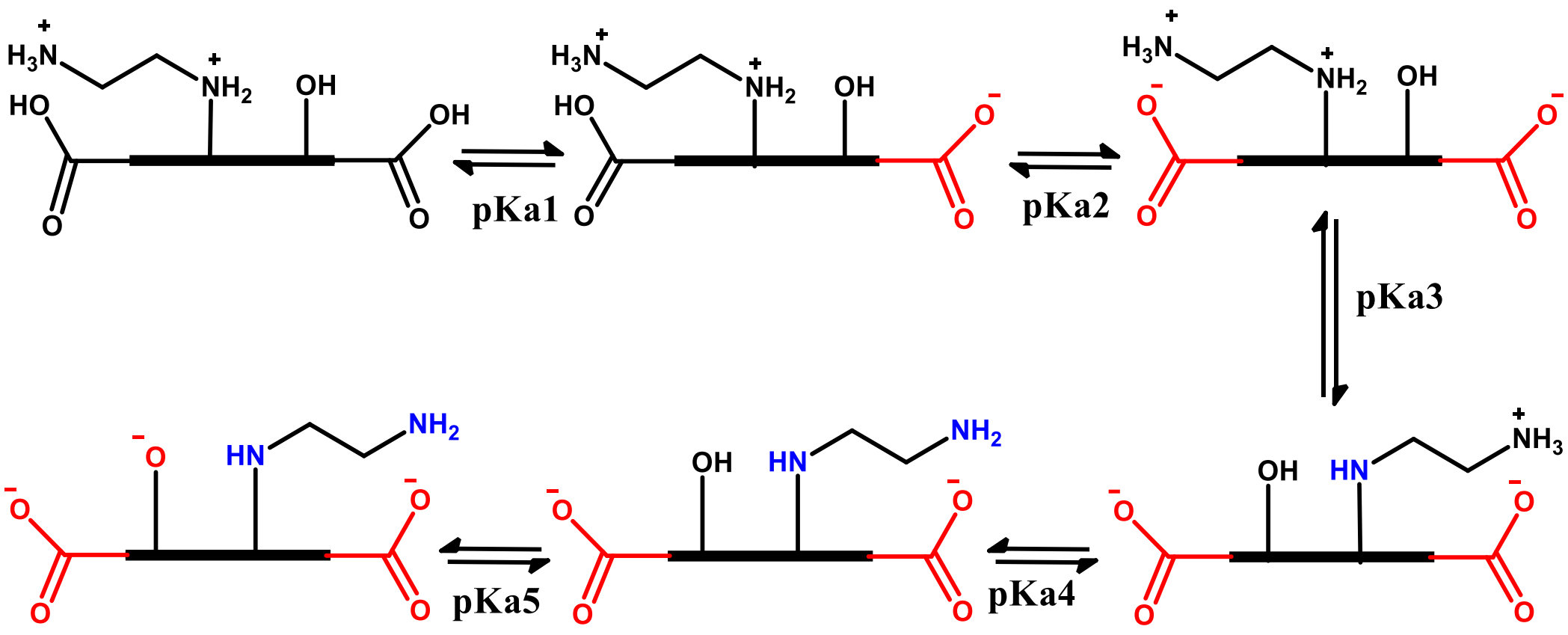

FIGURE S15 - Acid-basic equilibria proposed for GOEDA1.

Table S7 – pK_a obtained for each acid-base equilibrium.

	pK_{a1}	pK_{a2}	pK_{a3}	pK_{a4}	pK_{a5}
**GOEDA1**	4.02	6.02	6.88	8.87	9.53

8. UV spectra and kinetic studies of the pH profile

Figures S16 and S17 represent the kinetic profile of GOEDA2 and GOEtEDA. Figures S18 to 25 show the kinetic profile, alongside the pseudo-first order fitting of the reactions of GOEDA1 and DEDNPP forming the 4-DNP product (400 nm band).

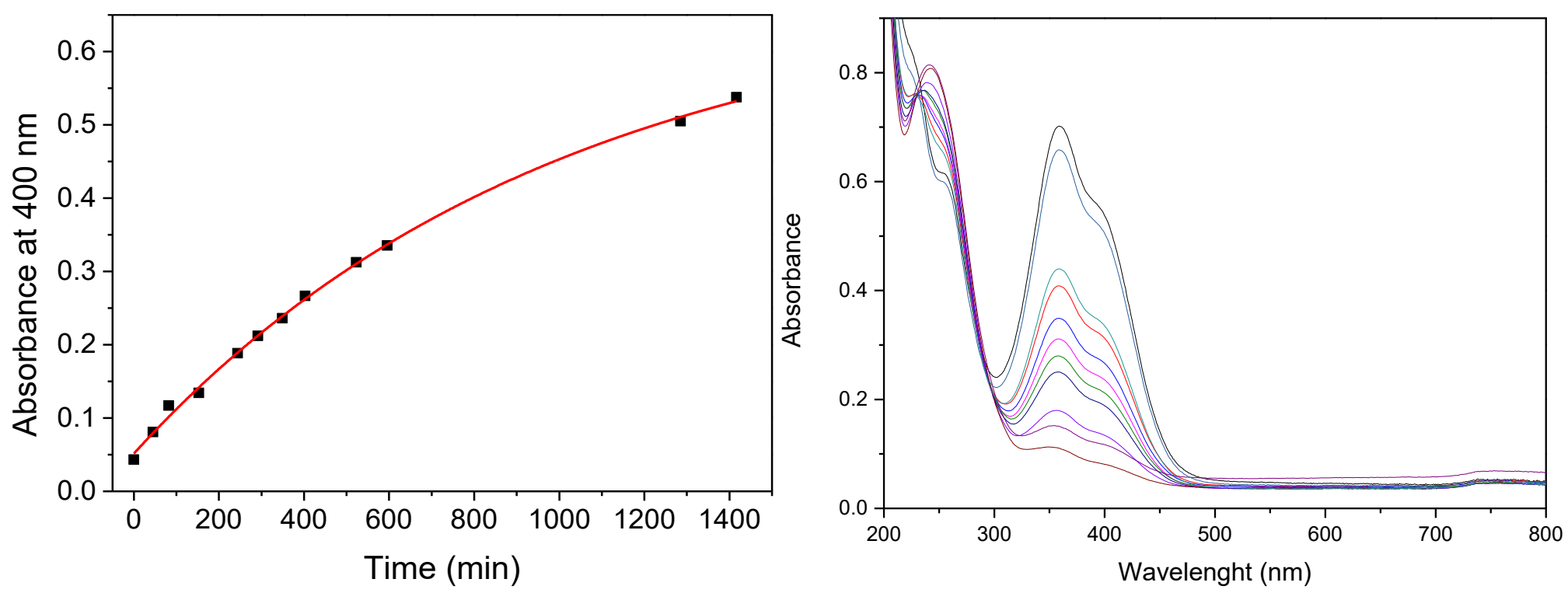

FIGURA S16-Kinetic profile and UV-Vis spectra obtained for the reaction with GOEDA2 at pH 8.5.

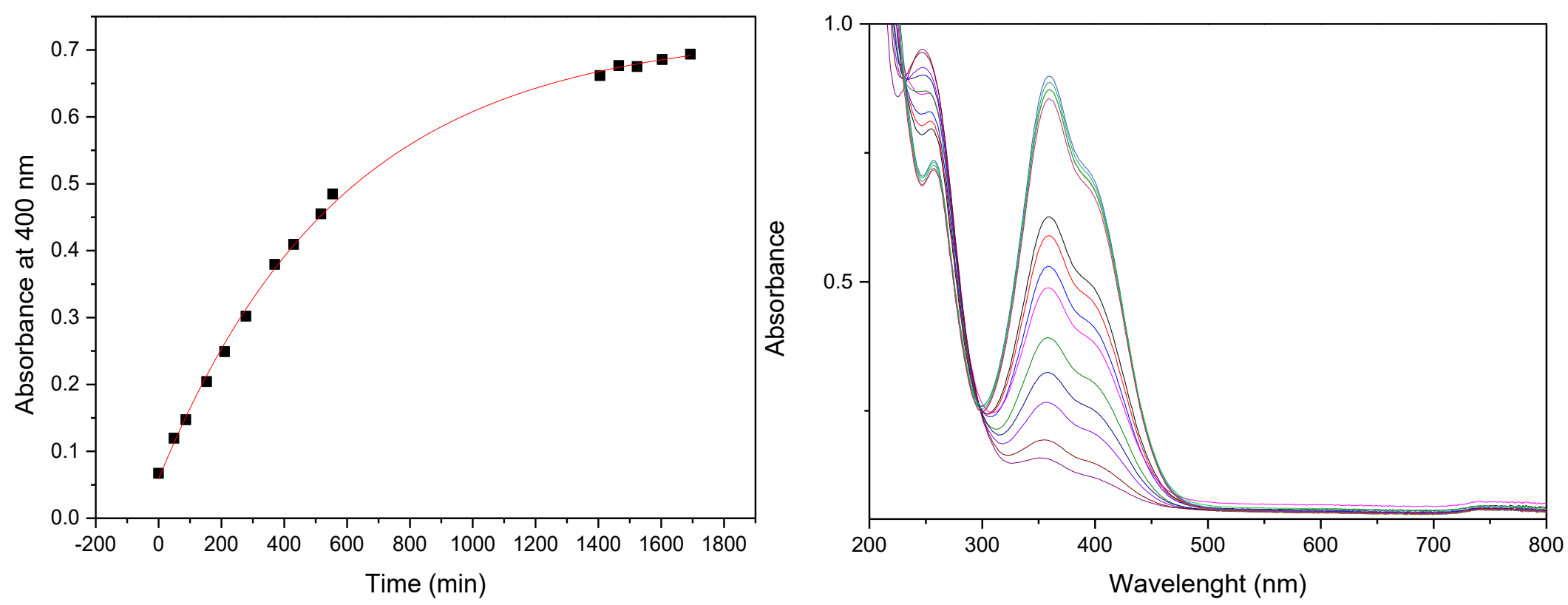

FIGURA S17 -Kinetic profile and UV-Vis spectra obtained for the reaction with GOEtEDA at pH 8.5.

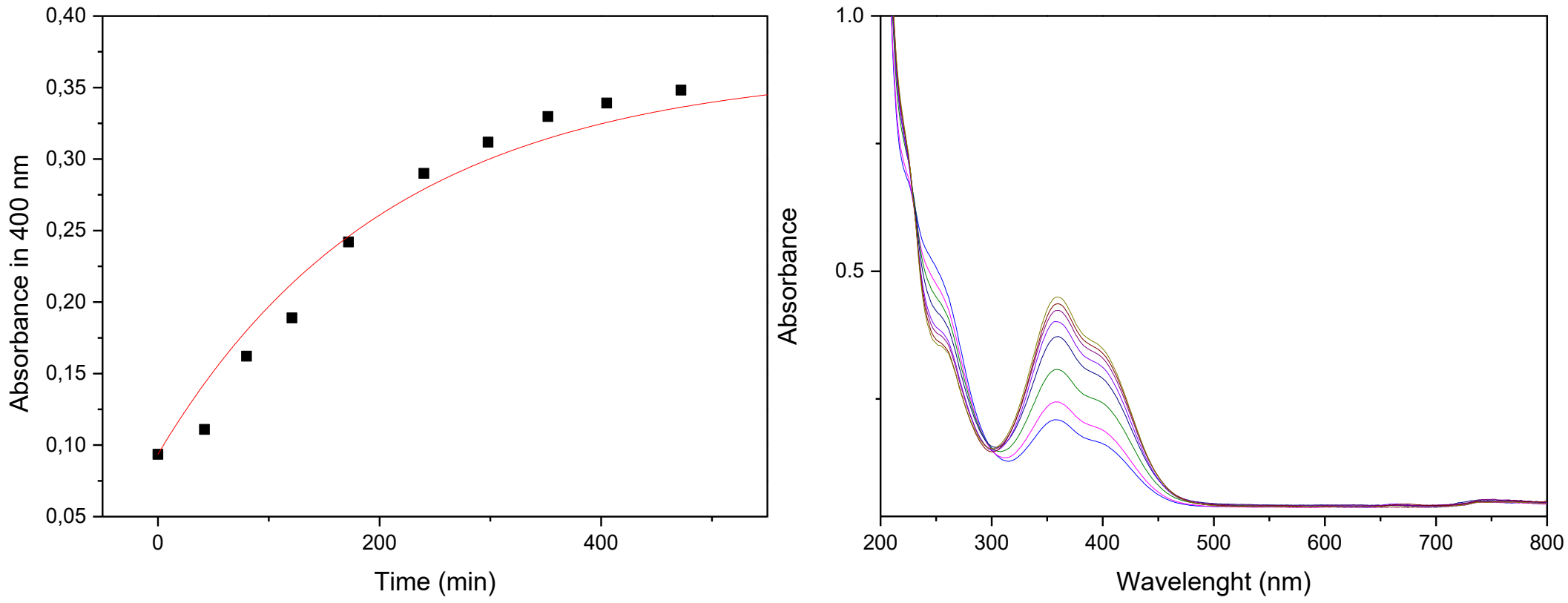

FIGURA S18-Kinetic profile and UV-Vis spectra obtained for the reaction of GOEDA1 and DEDNPP at pH 7.0

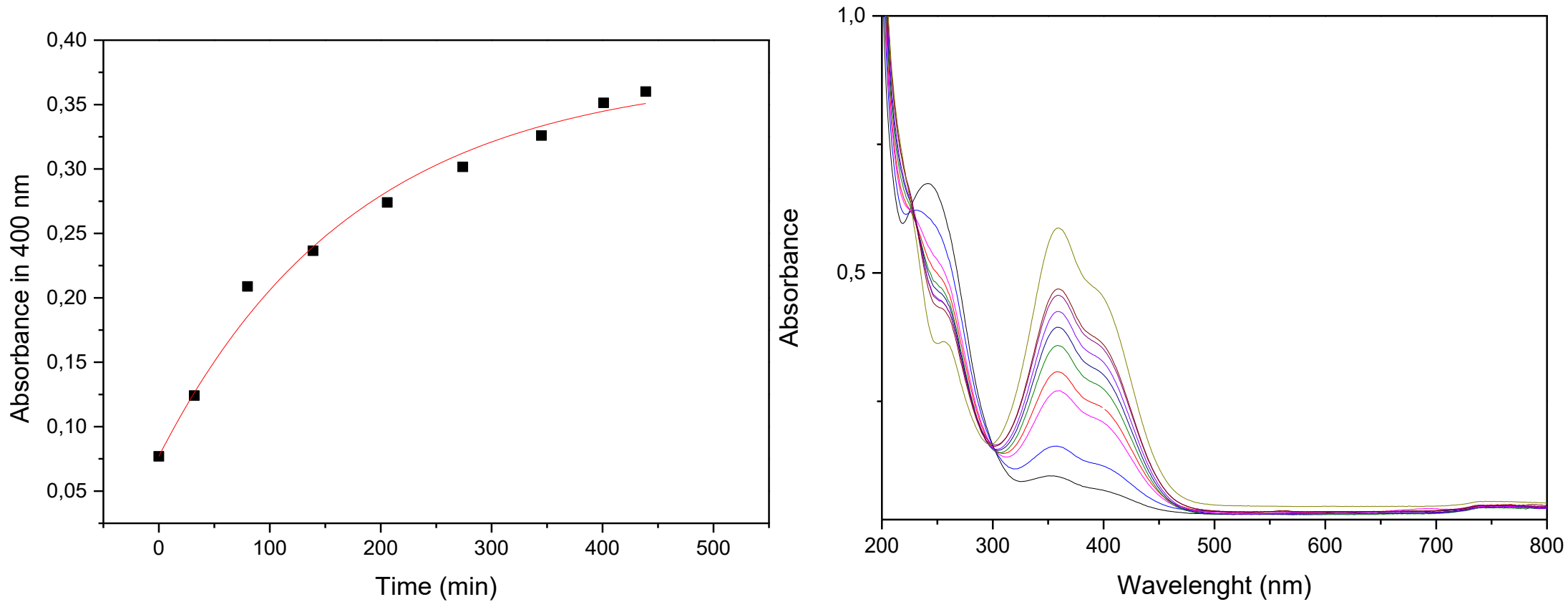

FIGURA S19- Kinetic profile and UV-Vis spectra obtained for the reaction of GOEDA1 and DEDNPP at pH 7.5

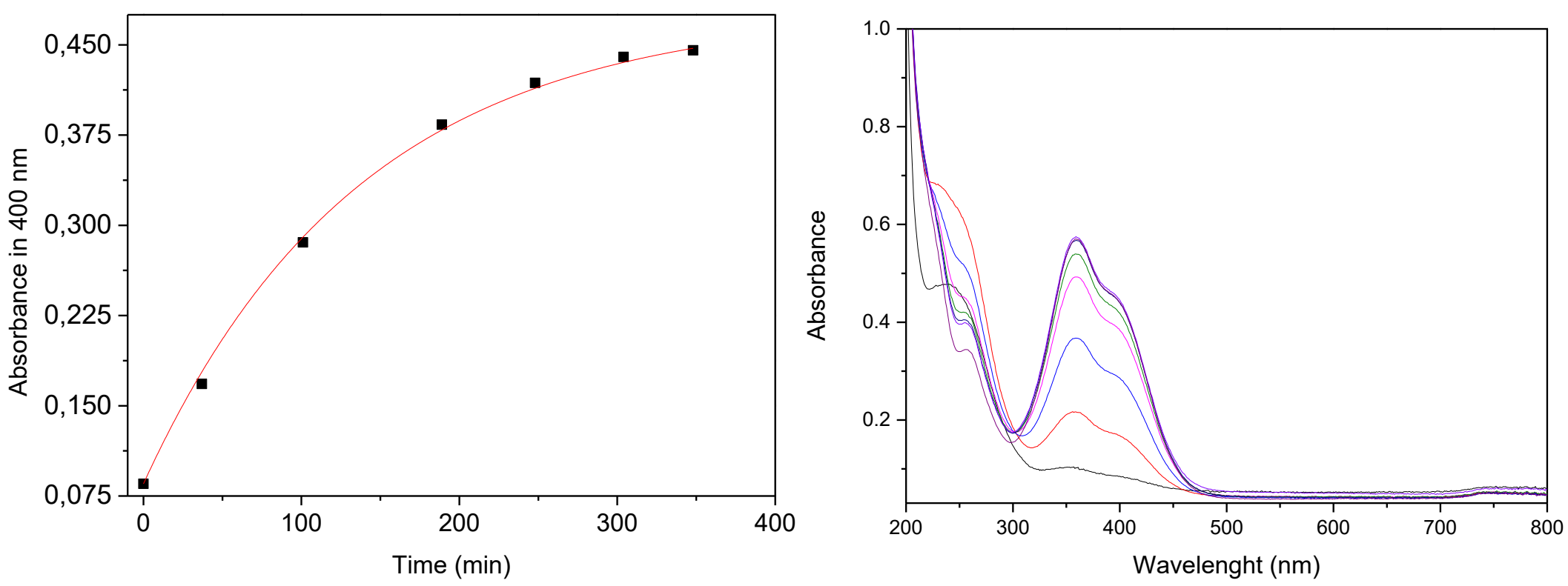

FIGURA S20- Kinetic profile and UV-Vis spectra obtained for the reaction of GOEDA1 and DEDNPP at pH 8.0

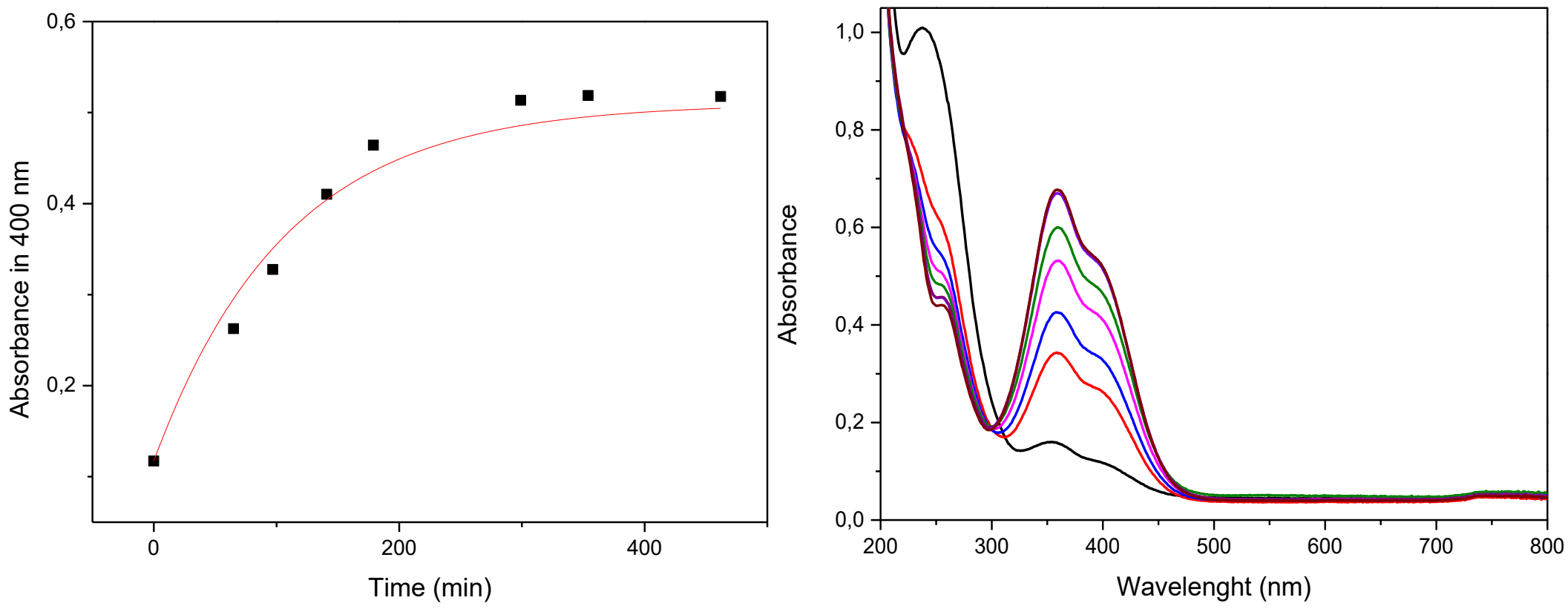

FIGURA S21- Kinetic profile and UV-Vis spectra obtained for the reaction of GOEDA1 and DEDNPP at pH 9.0

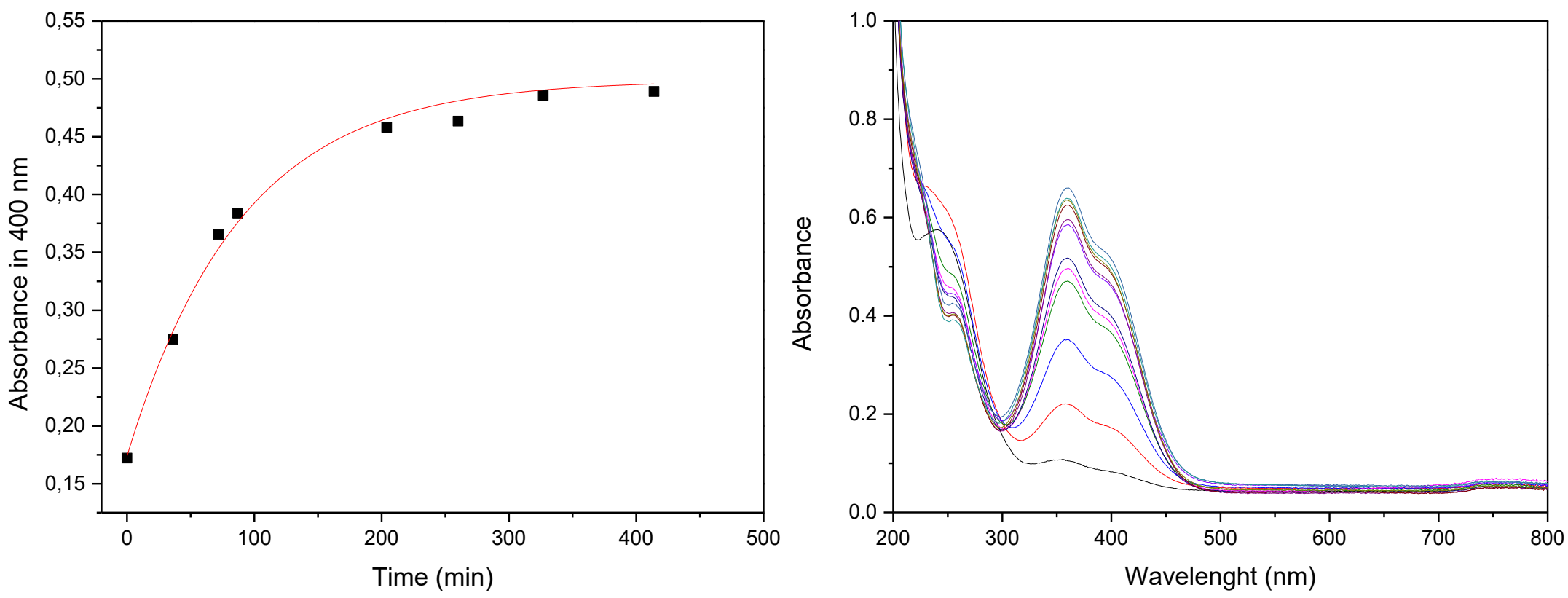

FIGURA S22- Kinetic profile and UV-Vis spectra obtained for the reaction of GOEDA1 and DEDNPP at pH 9.5

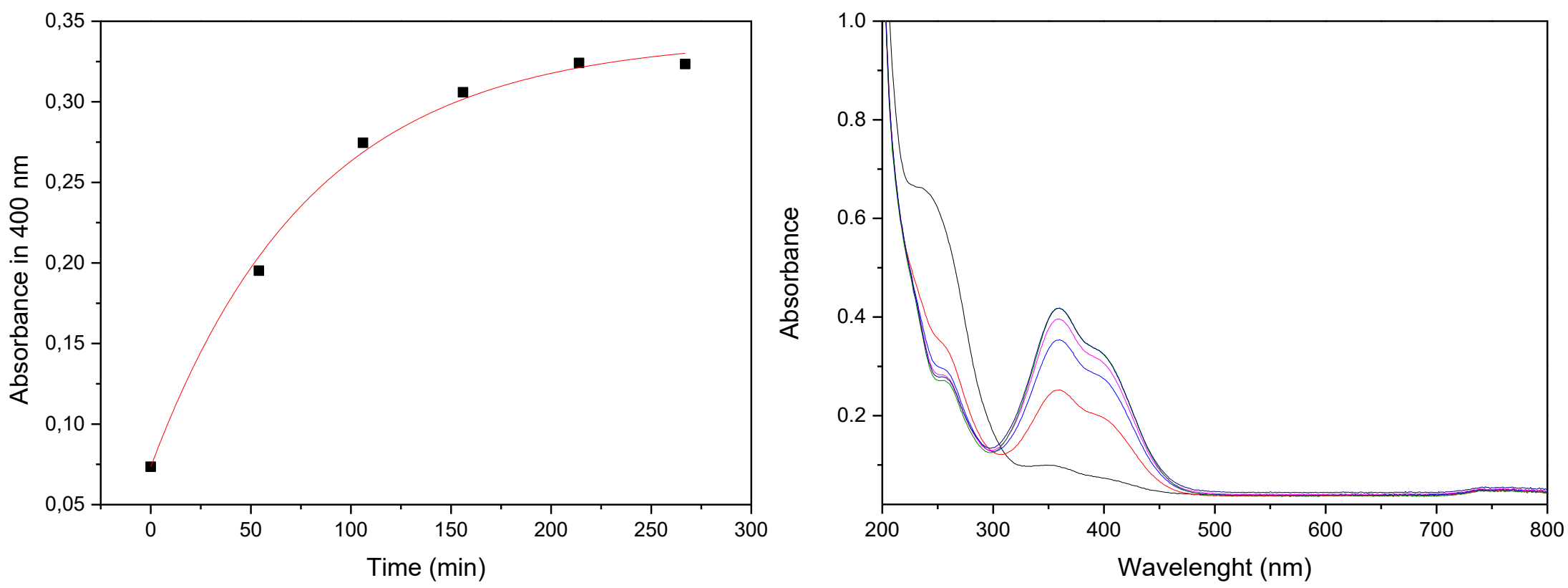

FIGURA S23- Kinetic profile and UV-Vis spectra obtained for the reaction of GOEDA1 and DEDNPP at pH 10.0

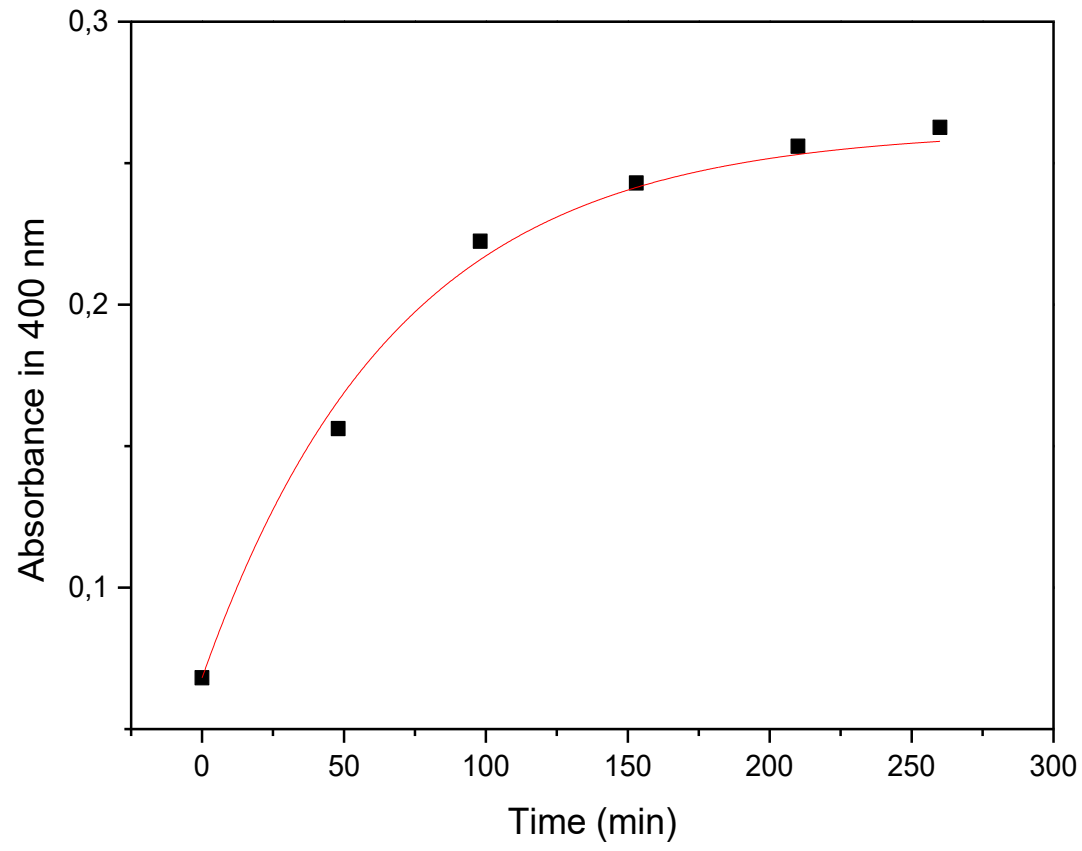

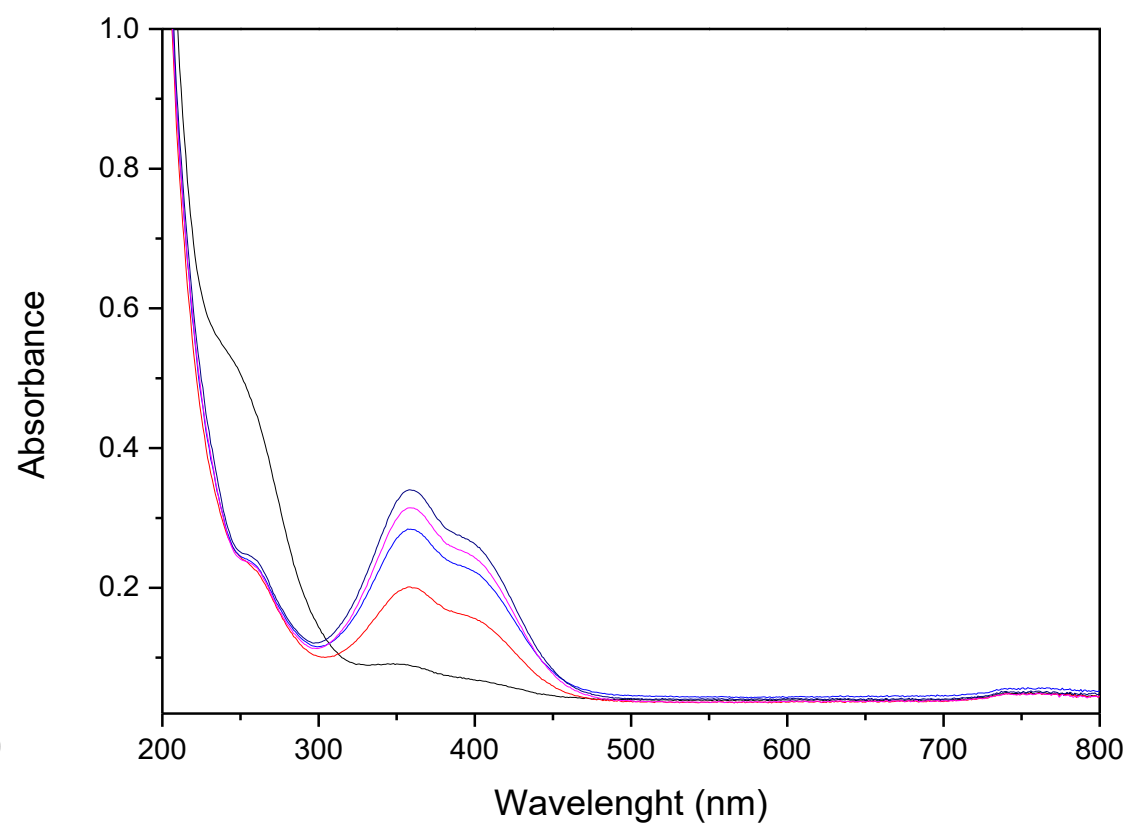

FIGURA S24- Kinetic profile and UV-Vis spectra obtained for the reaction of GOEDA1 and DEDNPP at pH 10.5

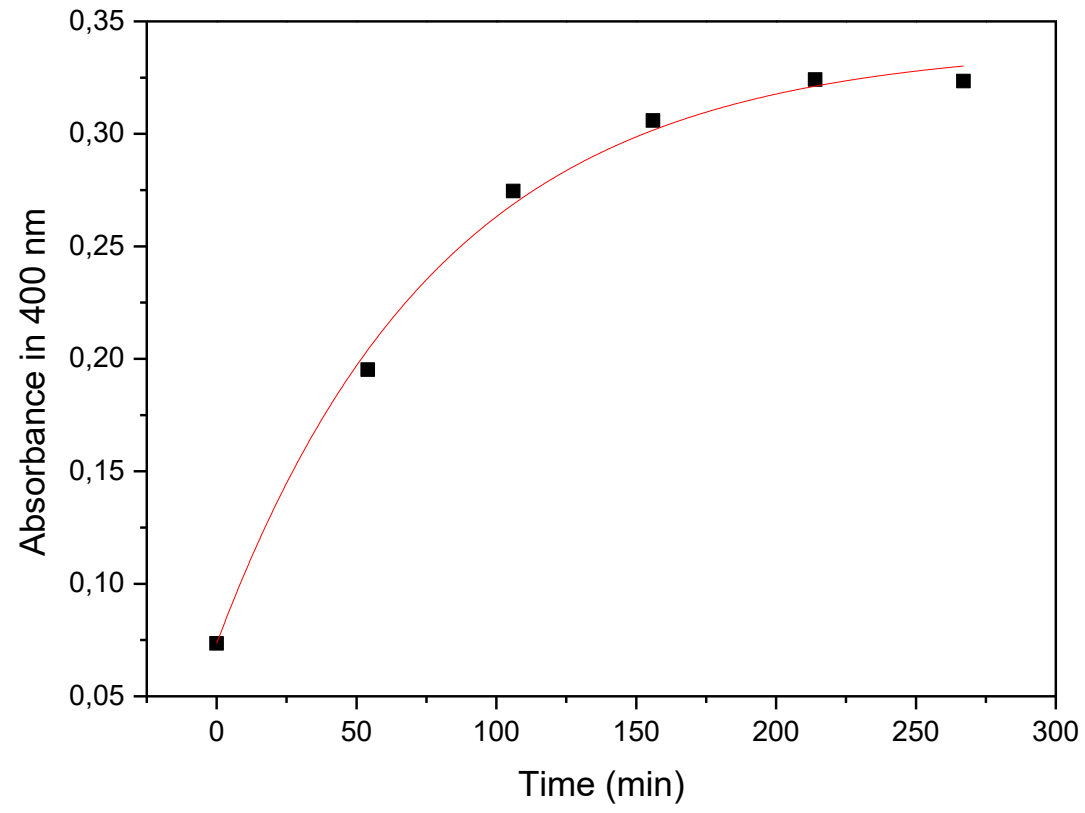

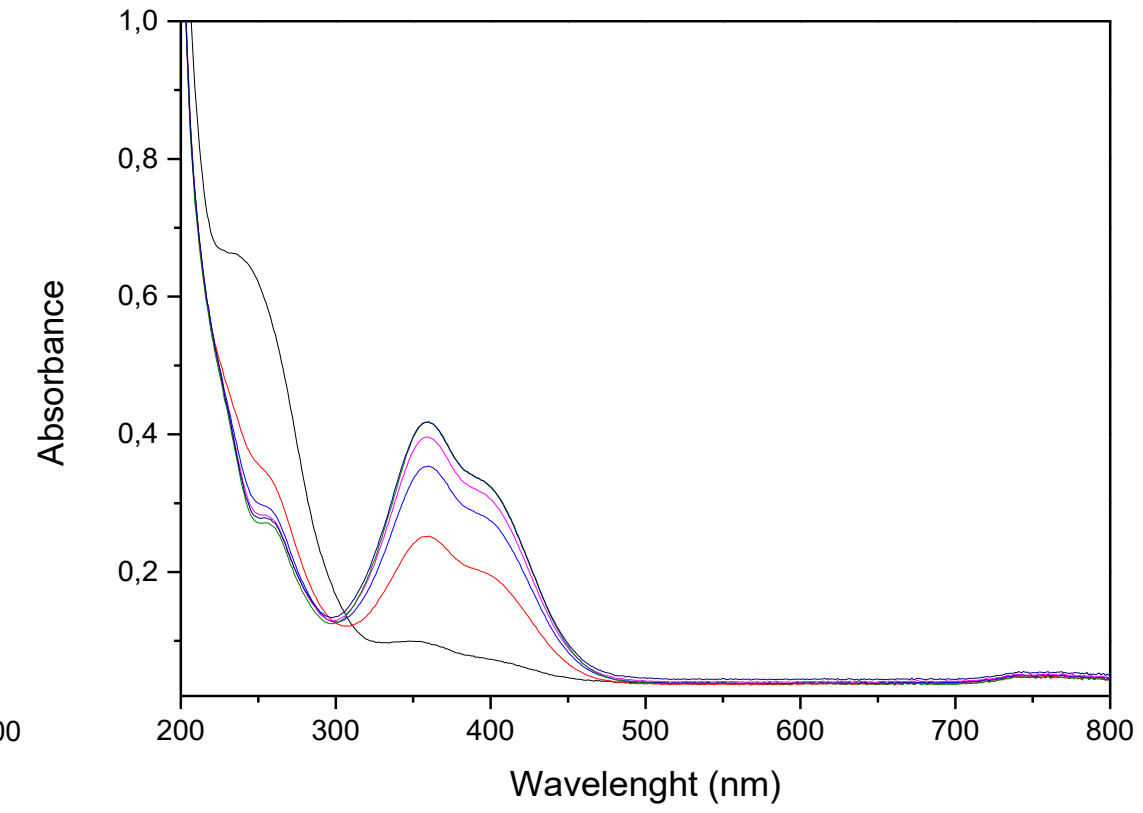

FIGURA S25- Kinetic profile and UV-Vis spectra obtained for the reaction of GOEDA1 and DEDNPP at pH 11.0

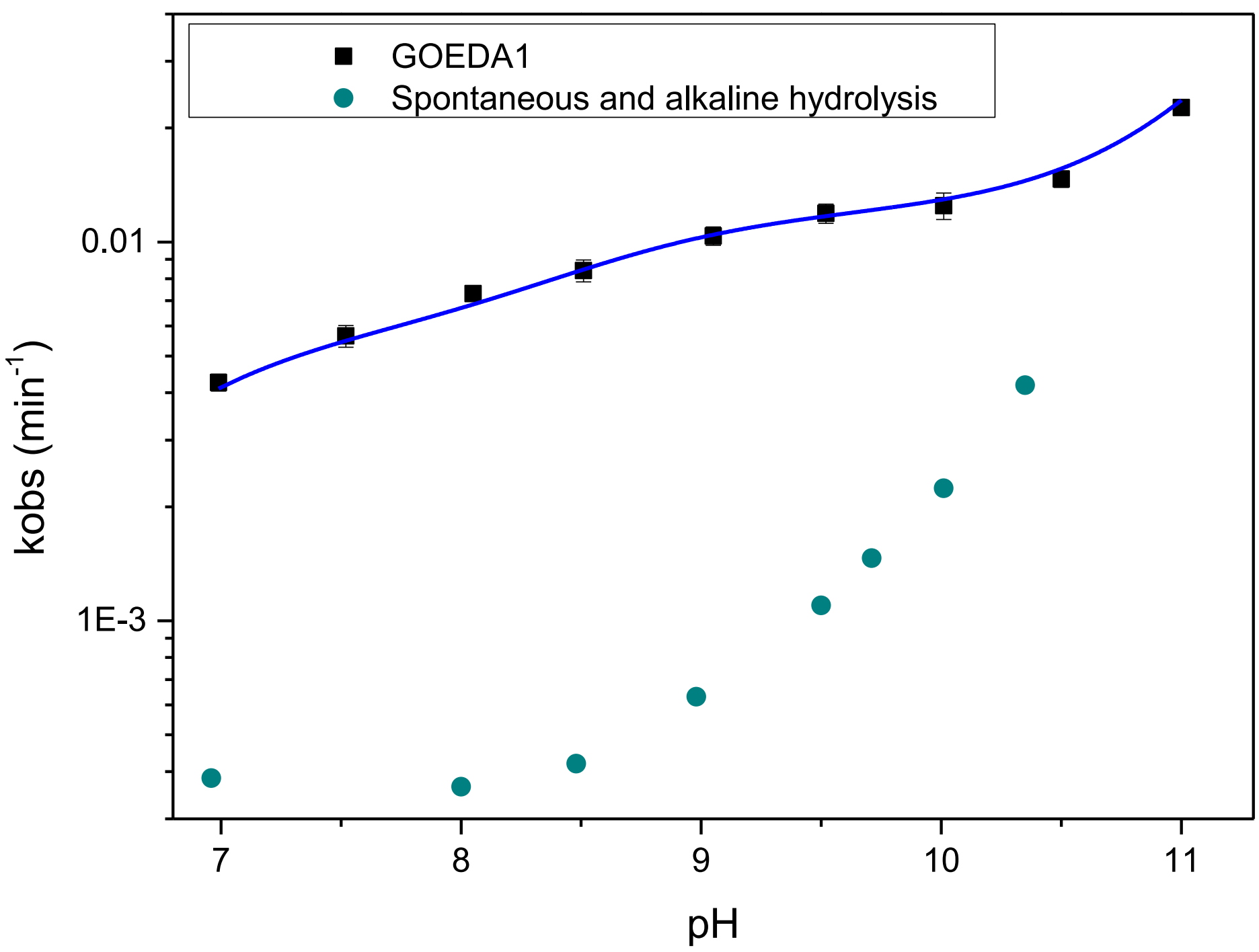

FIGURE S26– pH profile of the reaction of GOEDA1 with DEDNPP and pH profile of the spontaneous and alkaline hydrolysis. The blue line is relative to the adjustment made with equation 1.

$$\boldsymbol{k_{obs} = k_0 + k_{OH}[OH^-] + \frac{k_1 K_{a1}}{K_{a1}+K_{a1}[H^+]} + \frac{k_2 K_{a2}}{K_{a2}+K_{a2}[H^+]}} \quad \textbf{(S1)}$$

Where k_{obs} is the observed rate constant, k_0 is the spontaneous hydrolysis rate constant, k_{OH} is the alkaline hydrolysis rate constant, k_1 is r the primary amine rate constant, K_{a1} is the primary amine dissociation constant, k_2 is the secondary amine rate constant and K_{a2} is its dissociation constant.

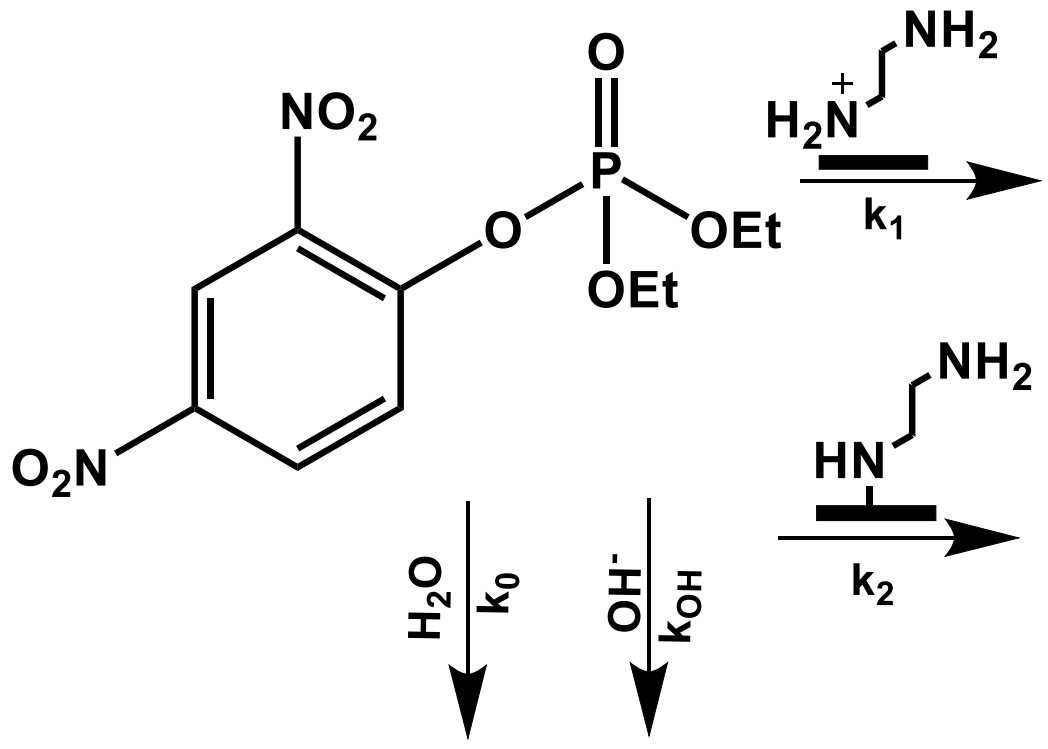

FIGURE S27– Schematic representation of the simultaneous reactions with GOEDA1 and DEDNPP.

Table S8. Kinetic parameters obtained from fitting data in Figure S24 with equation S1.

Constant	Value	Reference
k_0	3.74×10^{-3}	[8]
k_{OH}	11.7	[8]
k_1	0.00526	This work
k_2	0.00637	This work
K_{a1}	2.16×10^{-7}	This work
K_{a2}	2.52×10^{-9}	This work

The data was fitted considering the spontaneous and alkaline hydrolysis and the contribution of each different amine and can be described as the EQUATION S1[9]. Fitting to the data, made it possible to obtain the specific rate constant of each individual amine, being the secondary the most reactive one, with a k of 3,59 $g^{-1}min^{-1}$, the primary amine had a rate constant of 2,92 $g^{-1}min^{-1}$.

9. UV spectra and kinetic studies of the Recycling studies

These studies followed the same sample of GOEDA1 through 5 different reactions with DEDNPP at pH 9. Figure S28 presents the results, that there was almost no change in the k_{obs} obtained through the five reaction cycles; the error bars were obtained through the fitting equation. Figure S29 to 33 shows the kinetic profile, the pseudo-first order fitting and the UV-Vs spectra of each reaction conducted with the GOEDA1 sample. Figures 34-36 show the FTIR and SEM/EDS analysis before and after the cyles.

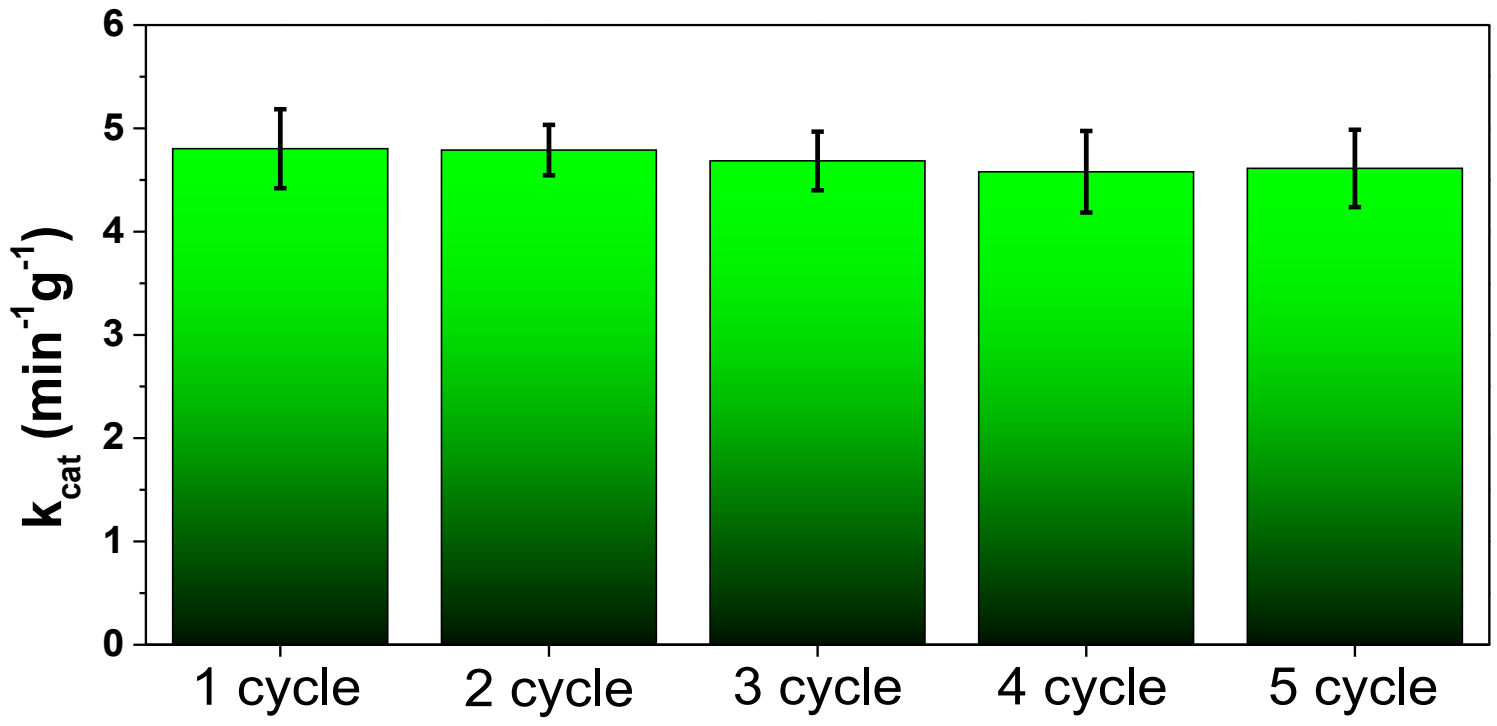

FIGURE S28– Recycling cycles of GOEDA1 with DEDNPP at p 9.0. The error bars were obtained from the pseudo-first order fitting.

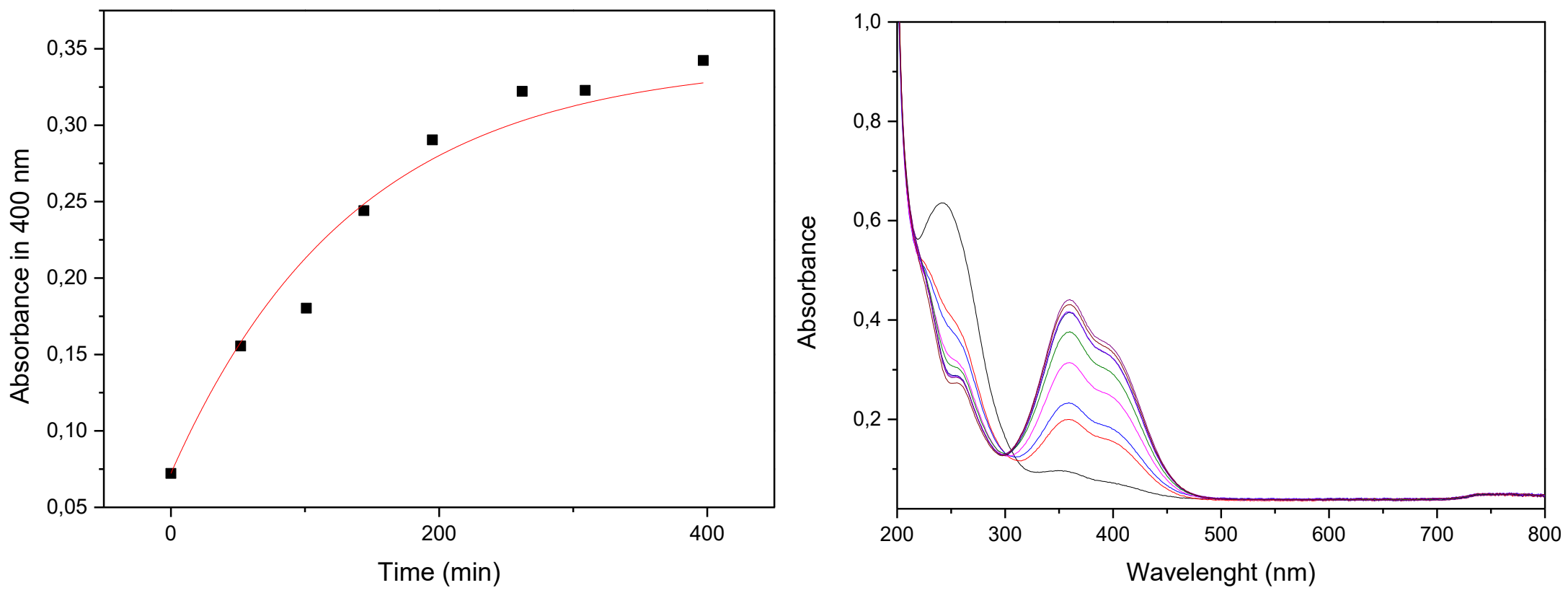

FIGURE S29– Spectra and kinetic profile obtained for GOEDA1 in the first cycle of use.

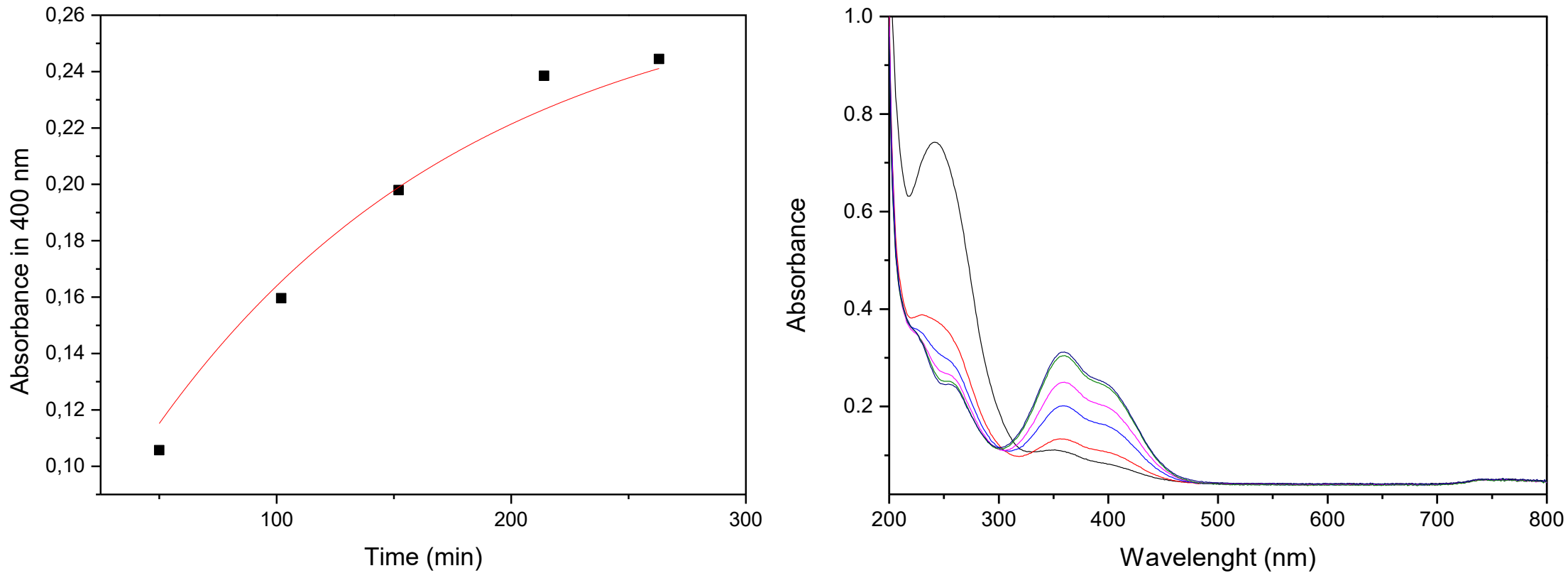

FIGURE S30- Spectra and kinetic profile obtained for GOEDA1 in the second cycle of use.

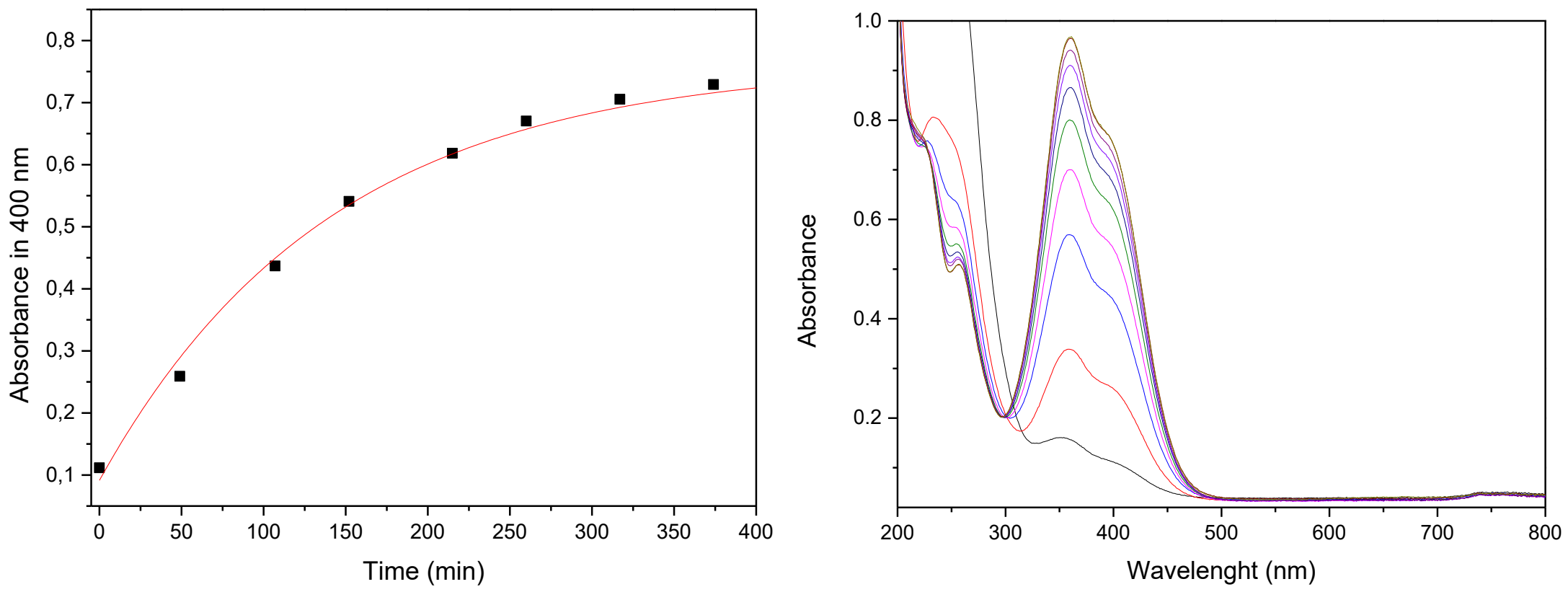

FIGURE S31- Spectra and kinetic profile obtained for GOEDA1 in the third cycle of use.

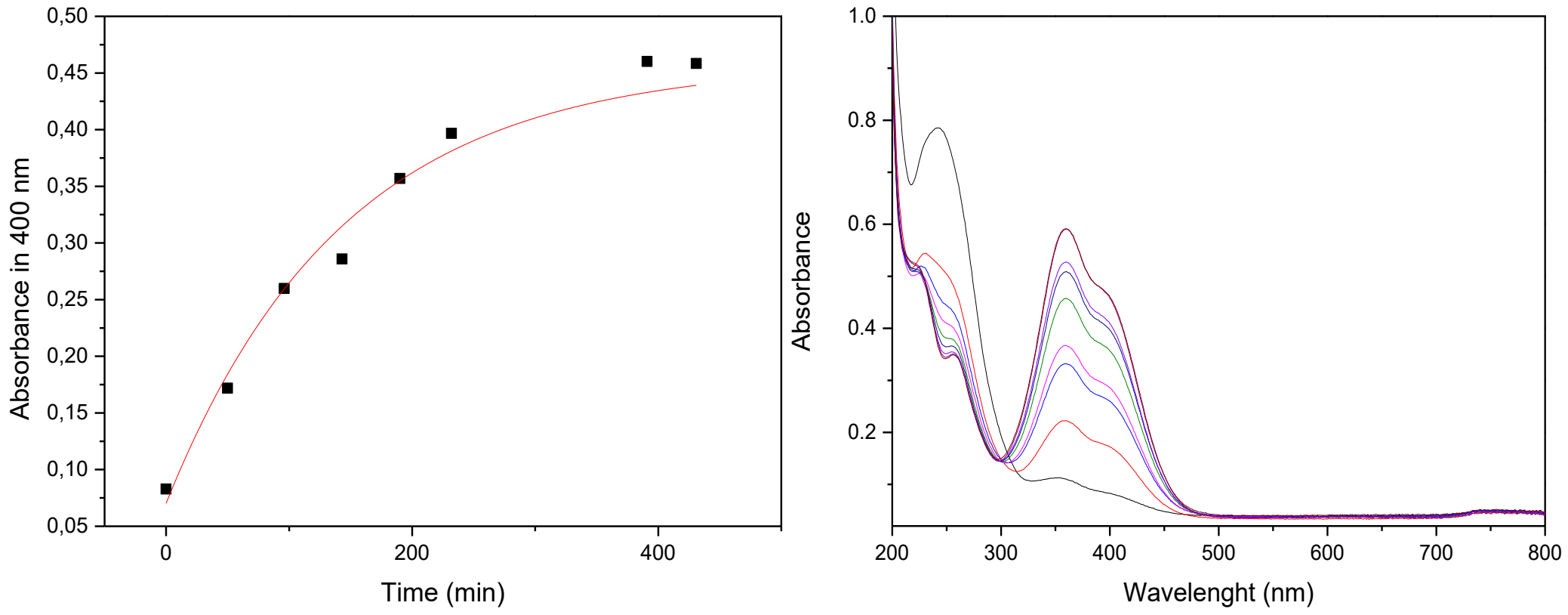

FIGURE S32- Spectra and kinetic profile obtained for GOEDA1 in the fourth cycle of use.

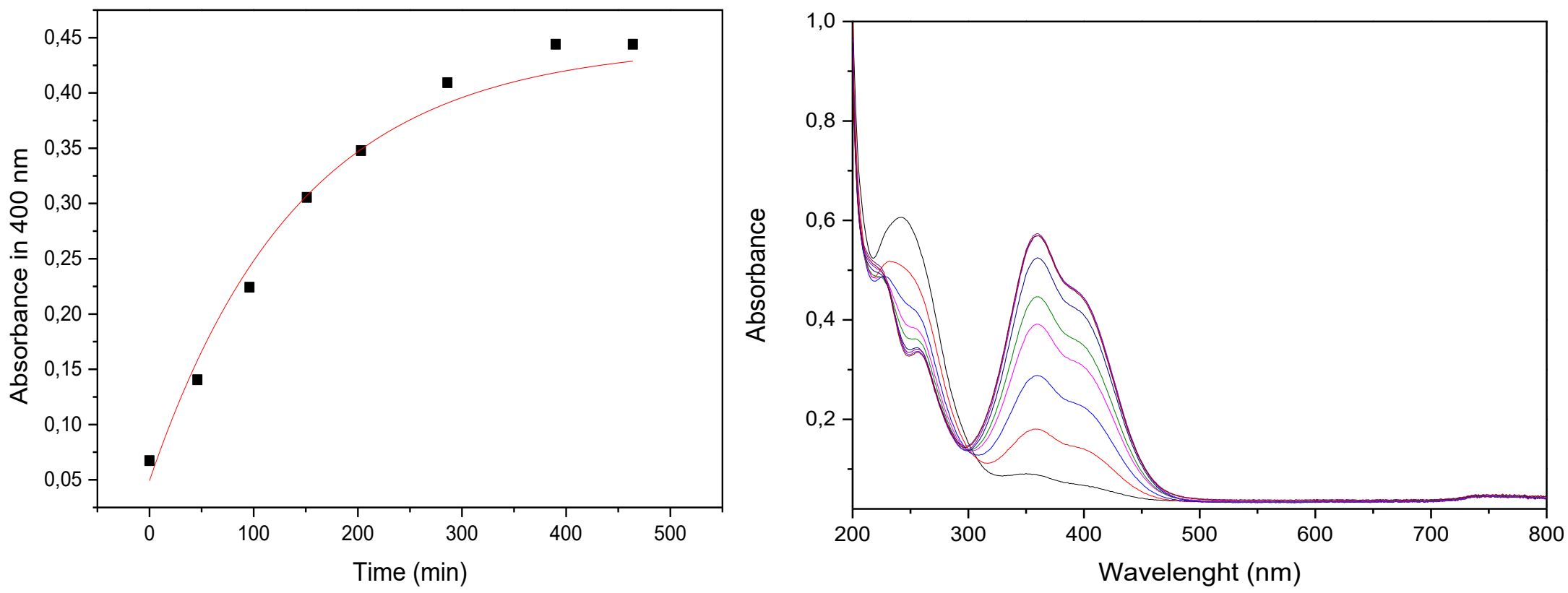

FIGURE S33- Spectra and kinetic profile obtained for GOEDA1 before and after the fifth cycle of use in the reaction with DEDNPP

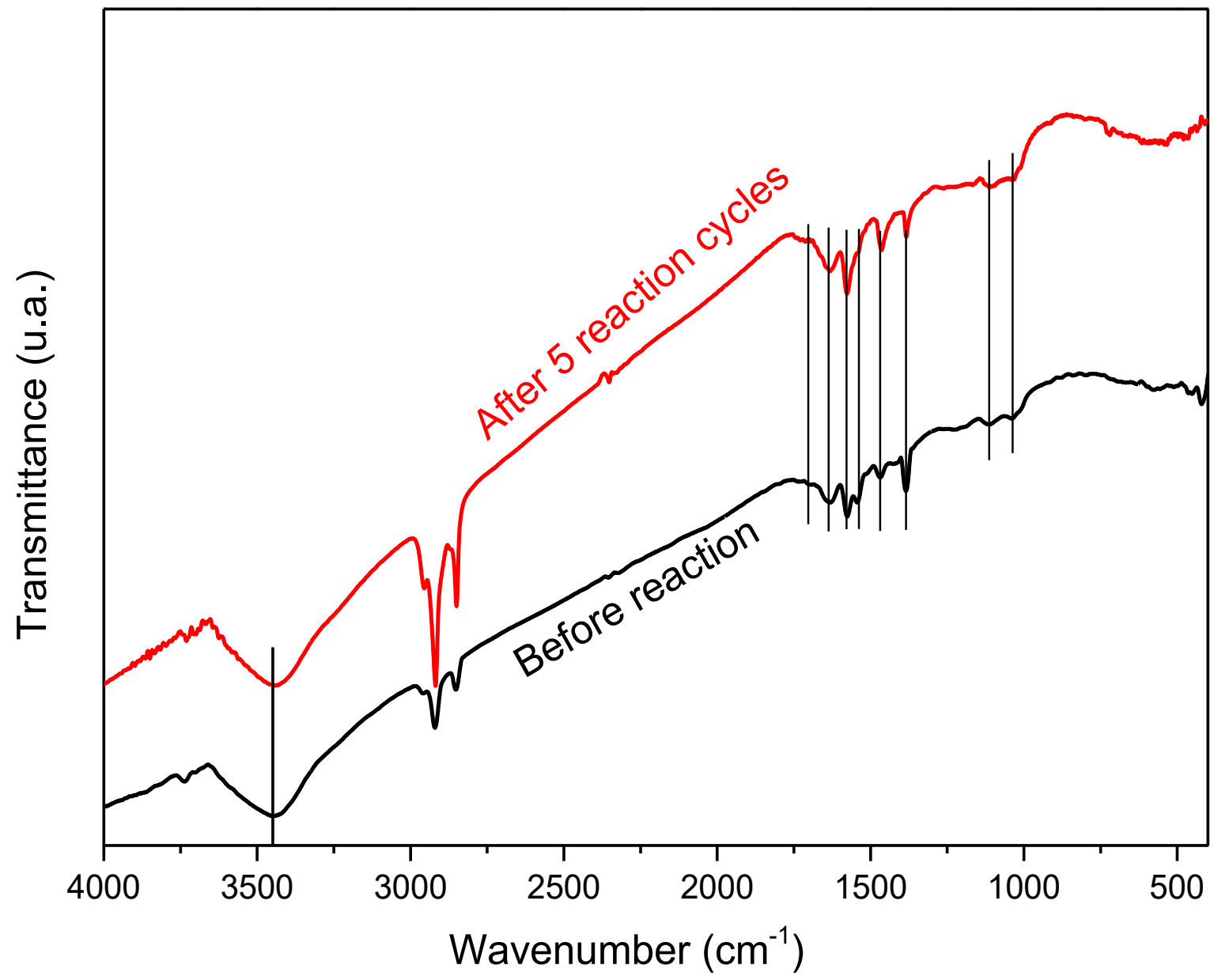

FIGURE S34 -. Infrared spectrum of GOEDA1 before and after the five cycles in the reaction with DEDNPP.

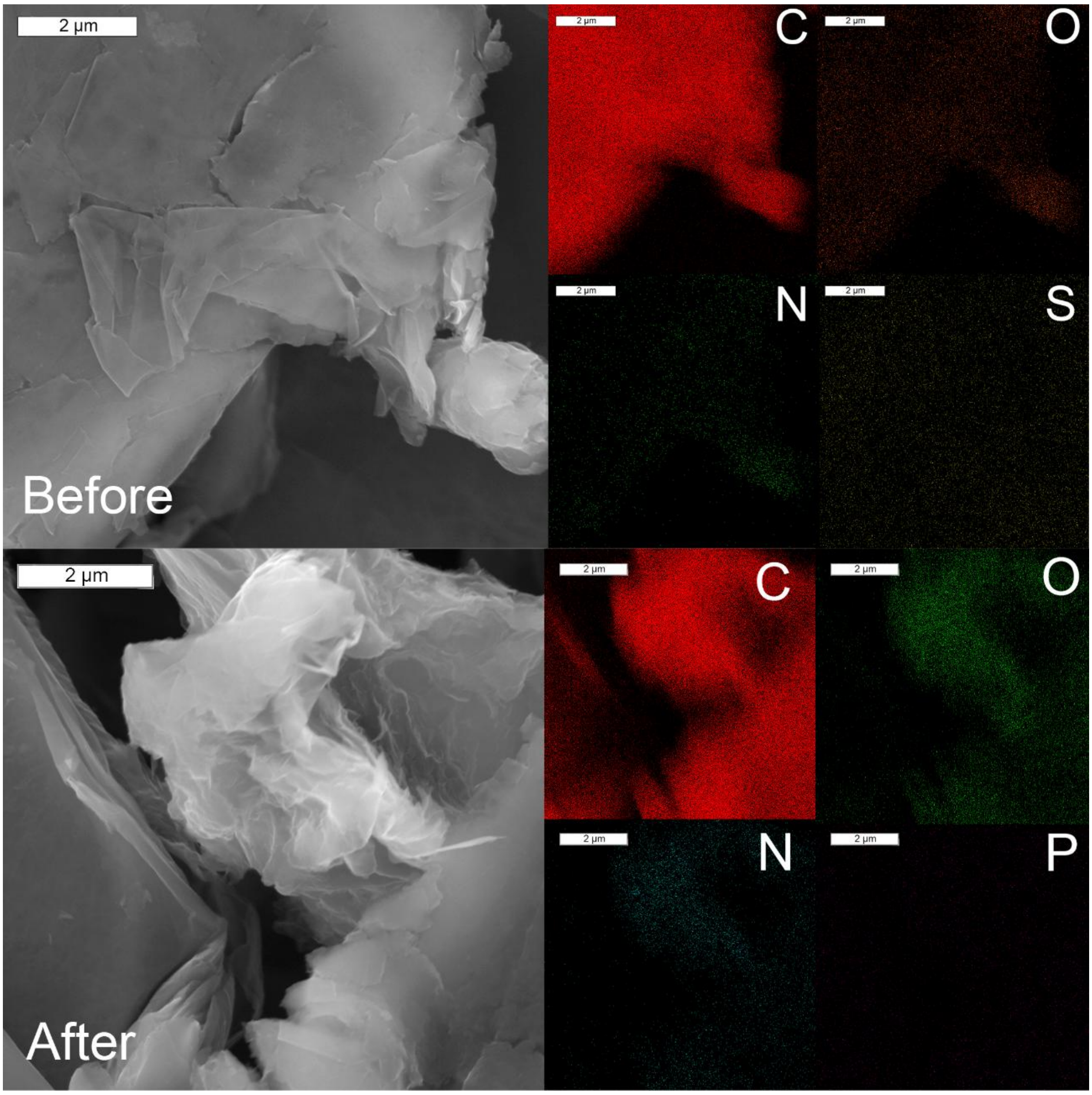

FIGURE S35 -. SEM and EDS images of GOEDA1 before and after the five cycles in the reaction with DEDNPP.

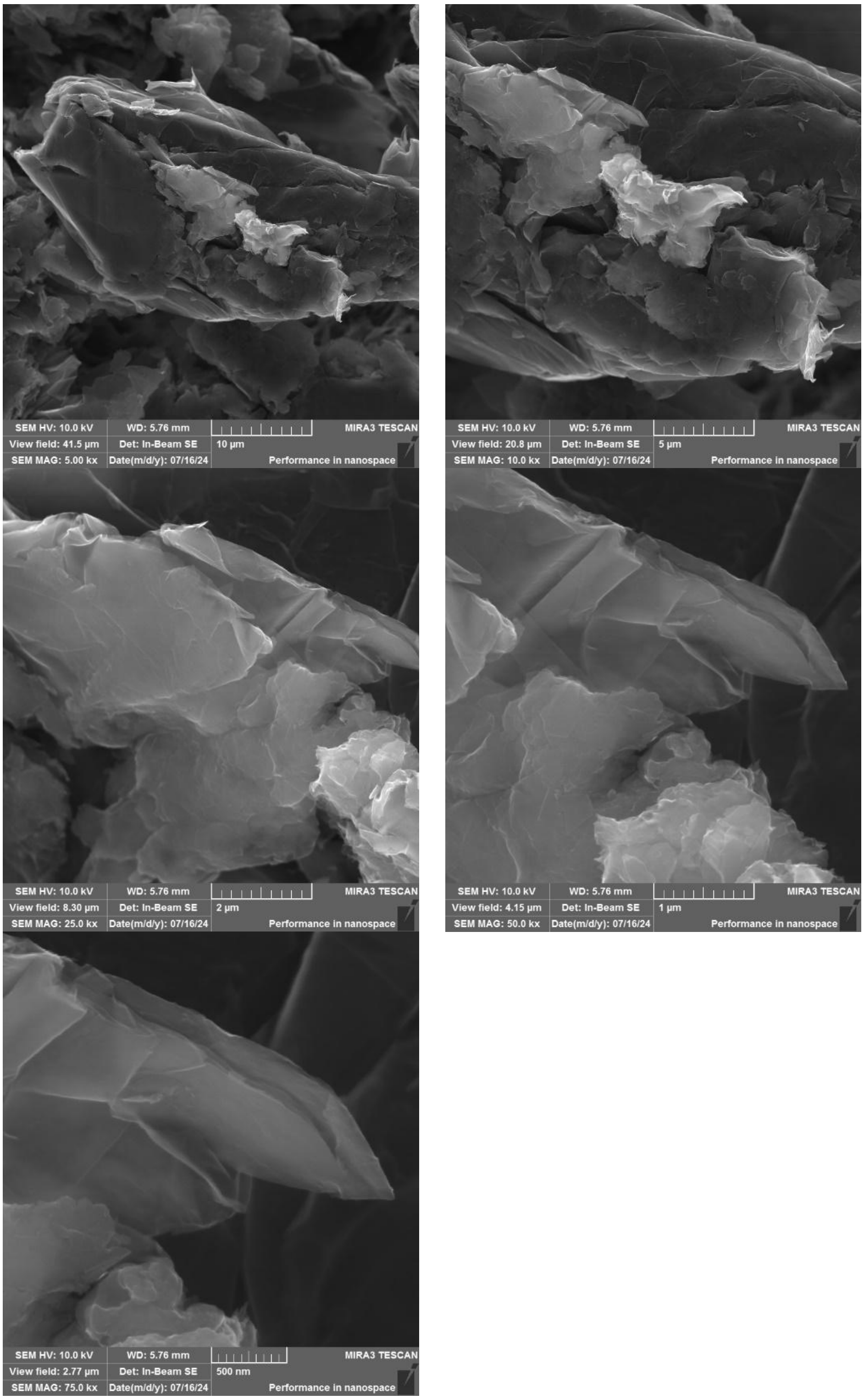

FIGURE S36 - SEM images of GEODA1 after five reaction cycles with DEDNPP at various amplifications

10. Selectivity studies

For the selectivity study, the reaction of GOEDA1 with 2,4-CNDB was studied. Figure S37 shows the possible pathways in this reaction. Its preferable to attack the phosphorus, since it effectively neutralizes the OP. Although another pathway is possible, with a nucleophilic attack at the aromatic carbon atom of DEDNPP, which can lead to several byproducts that can still be very toxic [10]. The reaction of GOEDA1 with CDNB is made to evaluate the attack towards the aromatic carbon. If this reaction happens, it can only occur through the nucleophilic attack on the aromatic carbon. Figure S38 shows consecutive spectra's, acquired through 72 h of reaction and it shows that there was no signal of the product 2,4-DNP, although there was a decrease in the DEDNPP signal (250 nm), that might indicate a slight absorption of the CDNB into GOEDA1. To study if it is relevant effect or not, the isolated product 2,4-DNP was added to an aliquot of GOEDA1 in pH 9, and after 72 h it was seen a decrease in the 400 nm band (FIGURE S39). But this adsorption was considered irrelevant, as the neutralization reaction of DEDNPP in pH 9.0 with GOEDA1 took around 6 h to finish.

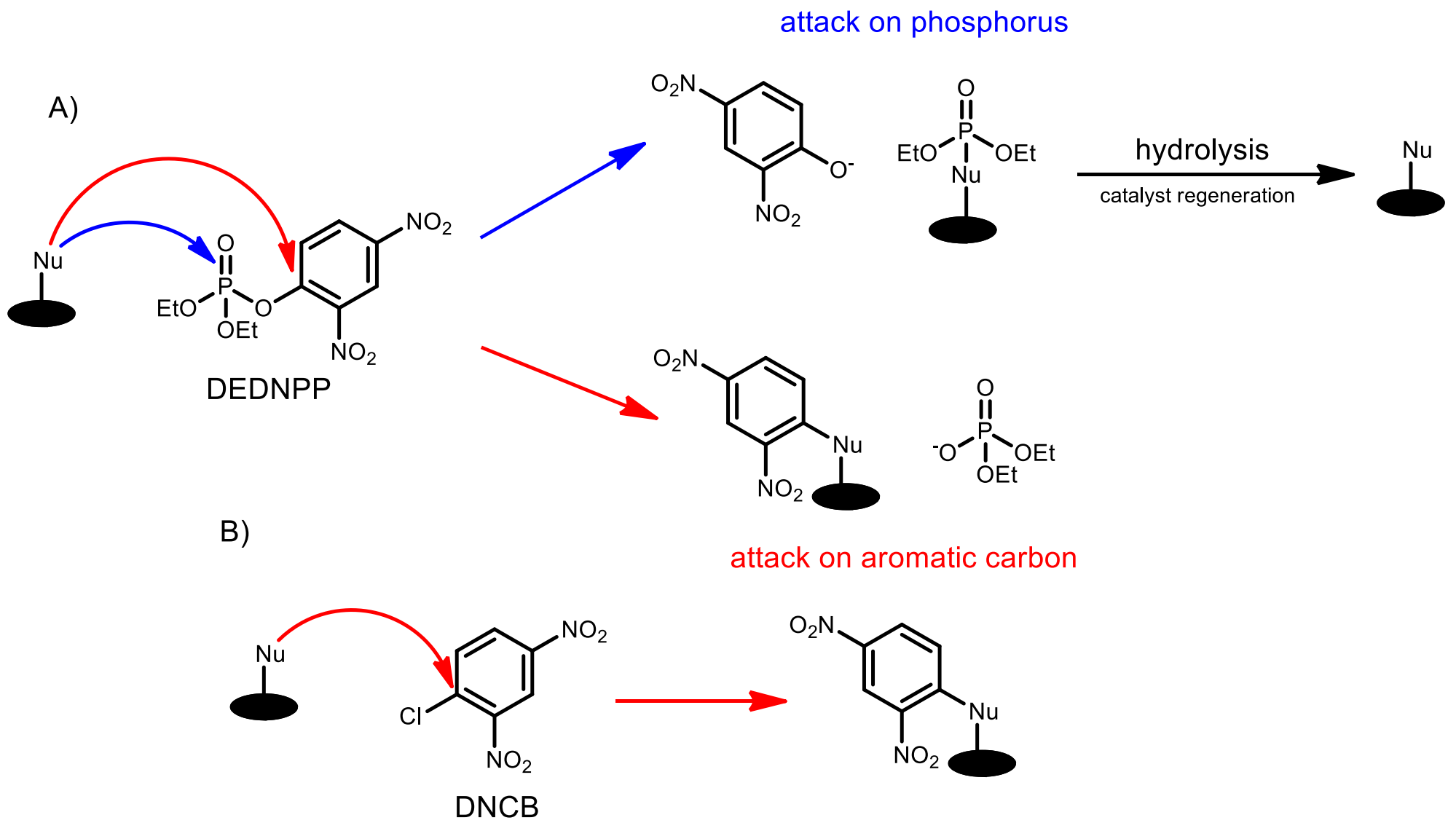

FIGURE S37 - Schematic representation of the possible routes studied. A) blue arrows show the nucleophilic attack on phosphorus. B) Red arrows represent the attack on the aromatic carbon.

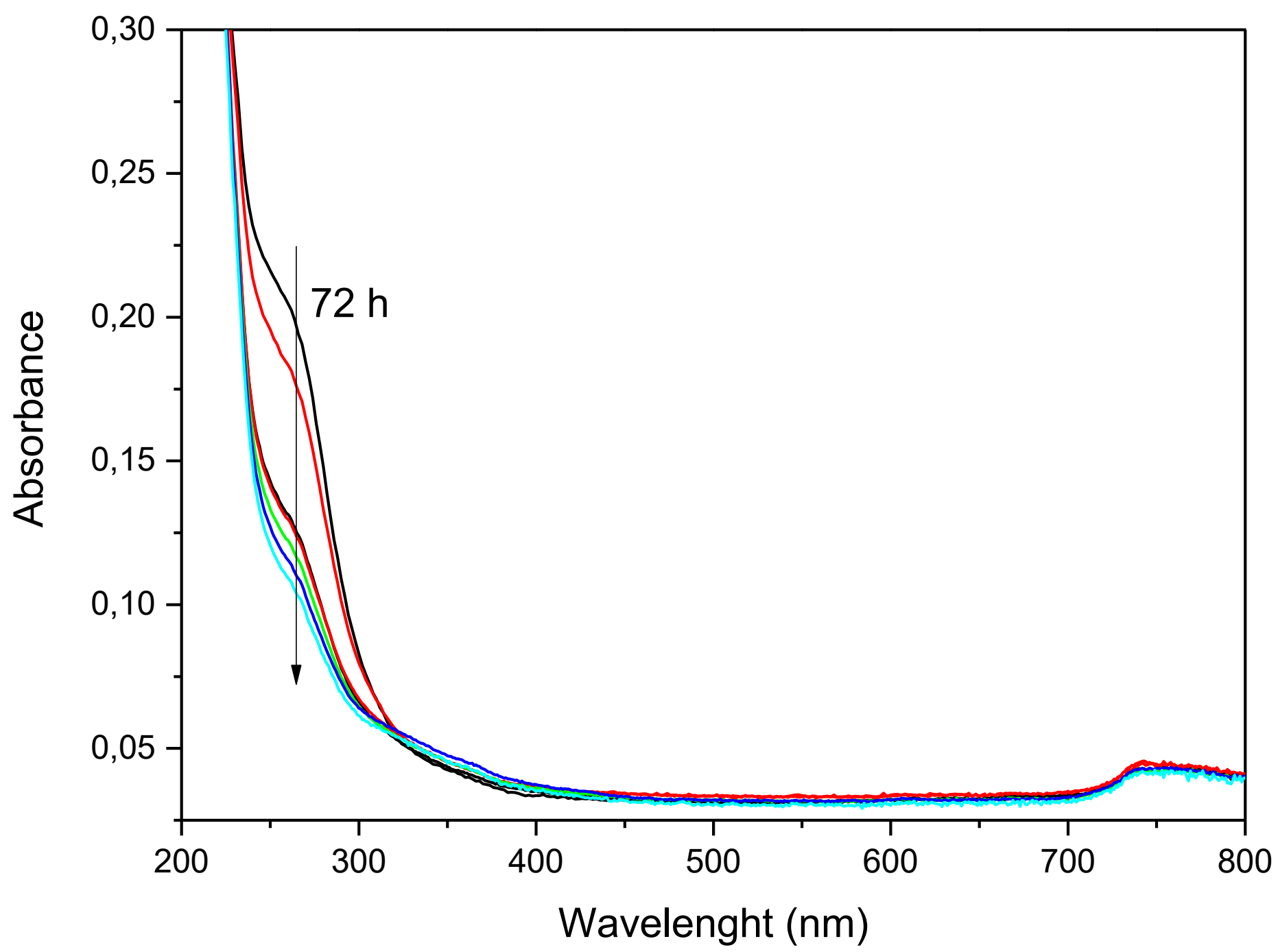

FIGURE S38 -Reaction of GOEDA1 with CNDB in pH 9.0.

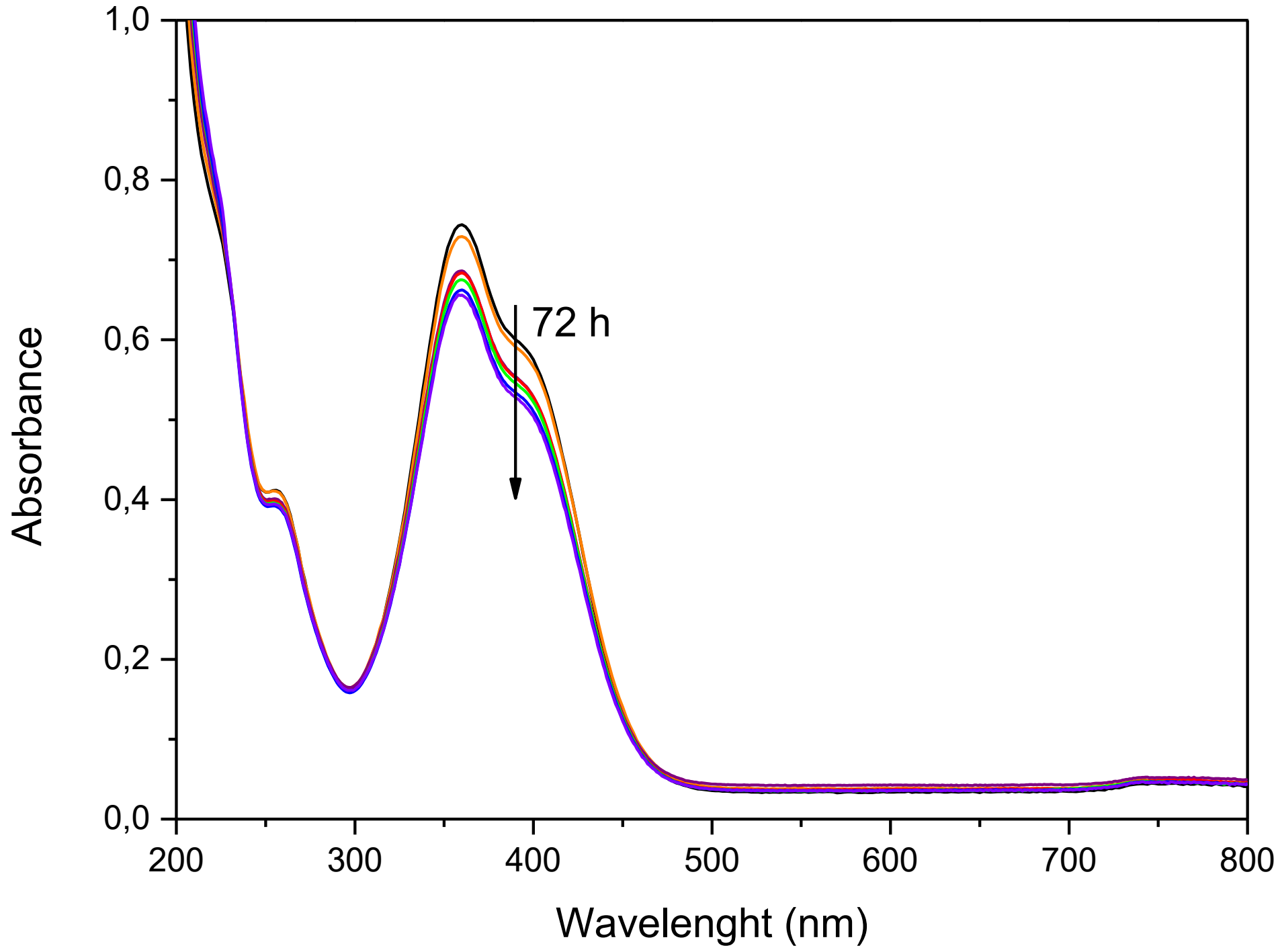

FIGURE S39 -GOEDA1 in the presence of 2,4-DNP in pH 9,0.

REFERENCES

[1] V. Ţucureanu, A. Matei, A.M. Avram, FTIR Spectroscopy for Carbon Family Study, Crit. Rev. Anal. Chem. 46 (2016) 502–520. https://doi.org/10.1080/10408347.2016.1157013.

[2] Y.H. Santos, A.H.G. Martinez, A.G. Veiga, M.L.M. Rocco, A.J.G. Zarbin, E.S. Orth, Neighboring Effects on the Selective Bifunctionalization of Graphene Oxide for Nanocatalytic Organophosphate Neutralization, ACS Appl. Nano Mater. 5 (2022) 6001–6012. https://doi.org/10.1021/acsanm.2c00832.

[3] Y.H. Santos, A.H.G. Martinez, A.G. Veiga, M.L.M. Rocco, A.J.G. Zarbin, E.S. Orth, Site-selective Mono- and Bifunctionalization of Graphene Oxide: Screening Nanocatalysts for Organophosphate Degradation, ChemCatChem 16 (2024). https://doi.org/10.1002/cctc.202301440.

[4] V. Brusko, A. Khannanov, A. Rakhmatullin, A.M. Dimiev, Unraveling the infrared spectrum of graphene oxide, Carbon N. Y. 229 (2024). https://doi.org/10.1016/j.carbon.2024.119507.

[5] J. Ederer, P. Janoš, P. Ecorchard, J. Tolasz, V. Štengl, H. Beneš, M. Perchacz, O. Pop-Georgievski, Determination of amino groups on functionalized graphene oxide for polyurethane nanomaterials: XPS quantitation vs. functional speciation, RSC Adv. 7 (2017) 12464–12473. https://doi.org/10.1039/c6ra28745j.

[6] E.S. Orth, J.G.L. Ferreira, J.E.S. Fonsaca, S.F. Blaskievicz, S.H. Domingues, A. Dasgupta, M. Terrones, A.J.G. Zarbin, PKa determination of graphene-like materials: Validating chemical functionalization, J. Colloid Interface Sci. 467 (2016) 239–244. https://doi.org/10.1016/j.jcis.2016.01.013.

[7] R.J. Motekaitis, A.E. Martell, Determination and Use of Stability Constants, 2nd Edition, VCH Publishers, New York, 1992.

[8] E.S. Orth, E.H. Wanderlind, M. Medeiros, P.S.M. Oliveira, B.G. Vaz, M.N. Eberlin, A.J. Kirby, F. Nome, Phosphorylimidazole Derivatives: Potentially Biosignaling Molecules, J. Org. Chem. 76 (2011) 8003–8008. https://doi.org/10.1021/jo2017394.

[9] A. Williams, J. Williams, Free Energy Relationships in Organic and Bio-organic Chemistry, The Royal Society of Chemistry, 2003. https://doi.org/10.1039/9781847550927.

[10] V.B. Silva, R.B. Campos, P. Pavez, M. Medeiros, E.S. Orth, Nucleophilic Neutralization of Organophosphates: Lack of Selectivity or Plenty of Versatility?, Chemical Record 21 (2021) 2638–2665. https://doi.org/10.1002/tcr.202100123.
